# Albumin-mediated alteration of plasma zinc speciation by fatty acids modulates blood clotting in type-2 diabetes†

**DOI:** 10.1039/d0sc06605b

**Published:** 2021-02-01

**Authors:** Amélie I. S. Sobczak, Kondwani G. H. Katundu, Fladia A. Phoenix, Siavash Khazaipoul, Ruitao Yu, Fanuel Lampiao, Fiona Stefanowicz, Claudia A. Blindauer, Samantha J. Pitt, Terry K. Smith, Ramzi A. Ajjan, Alan J. Stewart

**Affiliations:** School of Medicine, University of St Andrews Fife KY16 9TF St Andrews UK ajs21@st-andrews.ac.uk +44 (0)1334 463482 +44 (0)1334 463546; College of Medicine, University of Malawi Blantyre Malawi; Leeds Institute of Cardiovascular and Metabolic Medicine, University of Leeds Leeds UK; Key Laboratory of Tibetan Medicine Research, Northwest Plateau Institute of Biology, Chinese Academy of Sciences 23 Xinning Road Xining Qinghai 810001 China; Scottish Trace Element and Micronutrient Diagnostic and Research Laboratory, Department of Biochemistry NHS Greater Glasgow and Clyde Glasgow UK; Department of Chemistry, University of Warwick Coventry UK; School of Biology, Biomedical Sciences Research Complex, University of St Andrews St Andrews UK

## Abstract

Zn^2+^ is an essential regulator of coagulation and is released from activated platelets. In plasma, the free Zn^2+^ concentration is fine-tuned through buffering by human serum albumin (HSA). Importantly, the ability of HSA to bind/buffer Zn^2+^ is compromised by co-transported non-esterified fatty acids (NEFAs). Given the role of Zn^2+^ in blood clot formation, we hypothesise that Zn^2+^ displacement from HSA by NEFAs in certain conditions (such as type 2 diabetes mellitus, T2DM) impacts on the cellular and protein arms of coagulation. To test this hypothesis, we assessed the extent to which increasing concentrations of a range of medium- and long-chain NEFAs reduced Zn^2+^-binding ability of HSA. Amongst the NEFAs tested, palmitate (16 : 0) and stearate (18 : 0) were the most effective at suppressing zinc-binding, whilst the mono-unsaturated palmitoleate (16 : 1c9) was markedly less effective. Assessment of platelet aggregation and fibrin clotting parameters in purified systems and in pooled plasma suggested that the HSA-mediated impact of the model NEFA myristate on zinc speciation intensified the effects of Zn^2+^ alone. The effects of elevated Zn^2+^ alone on fibrin clot density and fibre thickness in a purified protein system were mirrored in samples from T2DM patients, who have derranged NEFA metabolism. Crucially, T2DM individuals had increased total plasma NEFAs compared to controls, with the concentrations of key saturated (myristate, palmitate, stearate) and mono-unsaturated (oleate, *cis*-vaccenate) NEFAs positively correlating with clot density. Collectively, these data strongly support the concept that elevated NEFA levels contribute to altered coagulation in T2DM through dysregulation of plasma zinc speciation.

## Abstract 2

Zn^2+^ is an essential regulator of coagulation. In plasma, Zn^2+^ availability is fine-tuned by human serum albumin (HSA). Here we show that elevated fatty acid levels contribute to altered coagulation in type-2 diabetes through Zn^2+^ mishandling by HSA.

## Graphical abstract

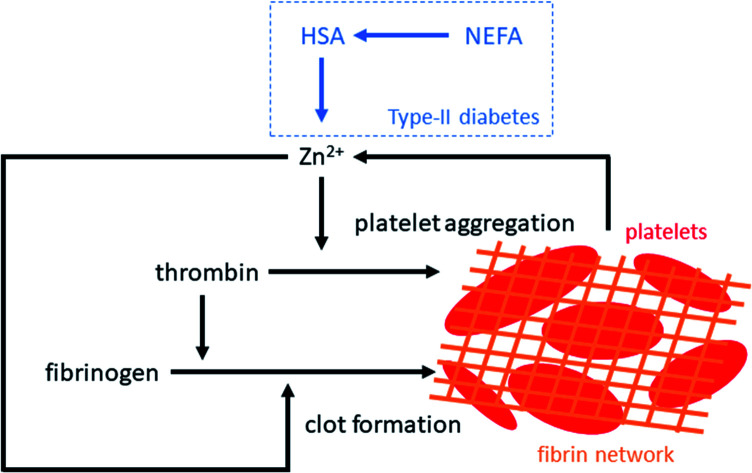

## 1. Introduction

Zinc is an essential modulator of coagulation controlling multiple aspects.^1^ Molecular regulation of coagulation by zinc is complex, as it binds numerous plasma proteins to influence their activities.^1^ More specifically, zinc has been shown to enhance platelet aggregation,^2^ accelerate clotting,^3^ and delay clot lysis.^4^ The fraction of zinc responsible for these effects is thought to be the “free” aquo ion of Zn^2+^, by binding to various biomolecules and their complexes. Low nanomolar free Zn^2+^ is cytotoxic;^5,6^ therefore, zinc is normally well-buffered in the extracellular space of mammals. In blood, this buffering role is mainly provided by human serum albumin (HSA). Typically, approximately 75% of the total 15–20 μM Zn^2+^ in plasma is bound to HSA,^7^ constituting >99% of the labile Zn^2+^ pool.^8^ Most of the remaining labile Zn^2+^ is bound to small molecules. The free Zn^2+^ concentration under resting conditions has been estimated as ∼1–3 nM,^9^ although more recently, the suitability of fluorescent dyes to accurately report free Zn^2+^ concentrations in the presence of high protein concentrations has been questioned.^10–12^ Irrespective of the actual true value, it is clear that labile and free Zn^2+^ concentrations in plasma are subject to highly dynamic spatial and temporal variations. Specifically during coagulation, they increase sharply around activated platelets that release Zn^2+^ from α-granule stores,^4,13^ with platelets accumulating in thrombi in numbers 50–100-fold higher than in circulating plasma.^3,4,14^ Zn^2+^ is also released by damaged epithelial cells, neutrophils, lymphocytes, erythrocytes and from ruptured atherosclerotic plaques.^15^ This results in a local increase in Zn^2+^ concentration which is difficult to estimate as this is a transient event: due to fast kinetics of binding, as soon as Zn^2+^ is released, it will bind to Zn^2+^-binding plasma proteins in its vicinity, including HSA and coagulation proteins.^15,16^

**Fig. 1:**
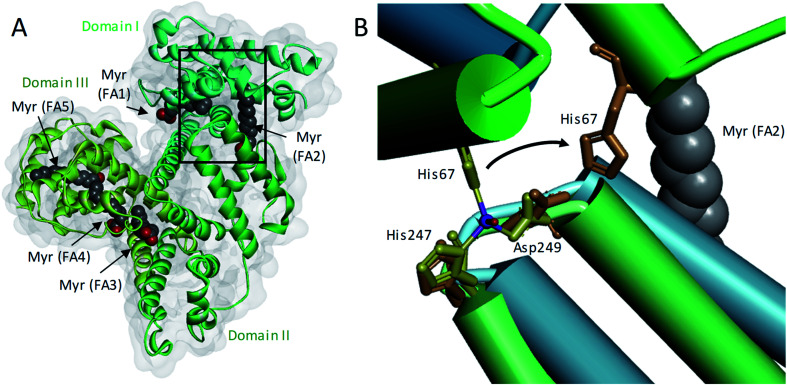
Effects of a NEFA binding at the FA2 site on the primary Zn^2+^-binding site of HSA. (A) Structure of HSA with 5 molecules of myristate bound at its medium (FA1 and 3) and high (FA2, 4 and 5) affinity fatty acid binding sites (PDB 1BJ5).^20^ The surface of the protein is shown in grey and the domains I, II and III are indicated with different green shades. (B) Overlay of the main Zn^2+^-binding site in myristate-bound HSA structure (in green, PDB 1BJ5) with a Zn^2+^-bound HSA structure (in blue, PDB 5IJF).^21^ The zinc ion is shown in purple and the oxygen from the water participating in Zn^2+^-coordination is in red. A structural change in the domain interface is induced by the binding of myristate to the FA2 binding site. This triggers the Zn^2+^-coordinating residue His67 to move ∼8 Å away from the other two Zn^2+^-coordinating residues.

In addition to binding/buffering Zn^2+^, HSA also transports non-esterified fatty acids (NEFAs) at 5 medium- to high-affinity binding sites (FA1-5), one of which (FA2) is located at the interface between domains I and II, close to the main Zn^2+^ binding site (site A).^17–19^ A secondary Zn^2+^ site with weaker affinity is also present on HSA, but is unlikely to contribute greatly to Zn^2+^ binding under normal conditions.^20^Fig. 1A shows the structure of HSA with myristate bound.^21^ When a NEFA molecule binds to FA2, the protein conformation changes to create a linear tunnel for the NEFA molecule that stretches across domains I and II; concomitantly, zinc site A is disrupted (the Zn^2+^-coordinating nitrogen of His67 (domain I) moves ∼8 Å relative to His247 and Asp249 (domain II; Fig. 1B)),^22^ dramatically reducing the Zn^2+^ affinity of HSA.^10,17–19^ Thus, when plasma NEFA levels are elevated, the Zn^2+^ buffering ability of HSA is reduced and plasma Zn^2+^ speciation is altered. Our recent work has shown that one consequence of this allosteric interaction between NEFAs and Zn^2+^ on HSA is the redistribution of Zn^2+^ among plasma proteins, including to histidine-rich glycoprotein, which is involved in coagulation control.^10,20^

Several disease states are associated with elevated plasma NEFA levels; these include cancer,^23^ obesity,^24^ non-alcoholic fatty liver disease,^25^ and type 2 diabetes mellitus (T2DM).^26^ These same disorders all associate with an increased risk of developing thrombotic complications (unwanted blood clots).^27,28^ These associations have been explained through the combinations of several different mechanisms, including NEFAs causing endothelial dysfunction, forming atherosclerotic plaques, hyperactivating platelets and directly dysregulating fibrin clot structure.^29–31^ We hypothesize that NEFA-mediated alterations to zinc dynamics in plasma also contribute to this pathology: the disruption of Zn^2+^ binding to HSA by elevated NEFAs leaves more Zn^2+^ to interact with coagulation proteins, which ultimately would be expected to enhance thrombotic risk.^27,28^ This hypothesis is supported by the observation that analbuminaemia (HSA deficiency) is associated with hypercoagulability, which is likely due to a combination of factors, including altered concentrations of coagulation proteins and altered metal ion speciation in plasma.^15,32^ In the present study, the ability of plasma NEFA levels to impact on Zn^2+^ handling and aspects of blood coagulability have been examined. To assess this, Zn^2+^ binding to HSA in the presence of various NEFAs was measured by isothermal titration calorimetry (ITC). Previously, such measurements have allowed us to quantify the effects of the model NEFA, myristate (C14 : 0) on Zn^2+^ binding to bovine serum albumin (BSA) and HSA.^18,20^ Myristate is a NEFA with low abundance in human blood; longer-chain NEFAs such as palmitate (C16 : 0) or stearate (C18 : 0) are much more abundant, and may bind with higher affinity.^33,34^ A few long-chain unsaturated NEFAs are also highly abundant, although high degrees of unsaturation are expected to lower their affinity to FA2.^35^ The efficiency with which a given NEFA is able to impede Zn^2+^ binding is expected to correlate with its affinity for site FA2, and hence is also dependent on chain length and degree of saturation. Therefore, the effects of several physiologically relevant saturated NEFAs, as well as some shorter-chain NEFAs and one unsaturated NEFA, on Zn^2+^ binding to HSA have been assessed in the present study.

To explore our central hypothesis that NEFAs impact blood clotting *via* an HSA-mediated effect on zinc speciation, platelet aggregation, and fibrin clot formation and lysis were assessed using carefully composed model systems containing HSA, Zn^2+^, and NEFAs that either affect (myristate) or do not affect (octanoate) Zn^2+^–HSA binding. Moreover, using a lipidomic approach, the relationship between plasma NEFA concentrations and coagulability in plasma samples taken from individuals with T2DM and controls (without diabetes) was investigated. Taken together, our results support the concept that elevated NEFA levels contribute to altered coagulation in T2DM through mishandling of plasma Zn^2+^.

## 2. Experimental

### 2.1. Ethical statement

Plasma samples from subjects with T2DM and controls were collected following approval by the National Research Ethics Service Committee Yorkshire & The Humber – Leeds East. Recruitment of healthy volunteers and blood sample collection for the platelet aggregation study was approved by the School of Medicine Ethics Committee, University of St Andrews. All blood samples were taken after obtaining written informed consent.

### 2.2. Isothermal titration calorimetry

Experiments were performed using a MicroCal iTC200 (Malvern Pananalytical, Malvern, UK), with HSA and Zn^2+^ both in a buffer containing 50 mM Tris (tris(hydroxymethyl)aminomethane), 140 mM NaCl, pH 7.4. Two sets of experiments were carried out: (1) titrating 1.5 mM ZnCl_2_ into 60 μM HSA in presence of 0–5 molar equivalents (mol. eq.) of octanoate, laurate, myristate or palmitate; (2) for less soluble NEFAs, 0.75 mM ZnCl_2_ were titrated into 25 μM HSA in presence of 0–5 mol. eq. of palmitate, palmitoleate or stearate. The NEFAs were diluted in either methanol or ethanol before being incubated with HSA in the reaction buffer for 2 h at 37 °C (1% final alcohol concentration). Heats of dilution were accounted for with blank titrations performed by injecting Zn^2+^ solution into reaction buffer and subtracting the averaged heat of dilution from the main experiments. Data fitting was performed using AFFINImeter software (Santiago de Compostela, Spain). Initial fitting was performed on the repeats of Zn^2+^/NEFA-free HSA titration and the average values obtained were used to fix K1, ΔH1 and N2 for the other titrations.^18,20^

### 2.3. Platelet aggregation assays

Platelets were isolated from whole blood collected in acid citrate dextrose or hirudin-coated collecting tubes from healthy donors. The blood was spun twice at 23 °C. The first spin was performed at 700 × *g* for 8 min to isolate platelet-rich-plasma, to which 0.32 U mL^−1^ apyrase and 100 μM acetylsalicylic acid were added to prevent premature platelet aggregation. The second spin was performed at 400 × *g* for 20 min to pellet the platelets. The platelets were washed and re-suspended in buffer solution (145 mM NaCl, 5 mM KCl, 1 mM MgCl_2_, 10 mM HEPES, 1 mM CaCl_2_, 10 mM d-glucose, pH 7.4) for washed-platelet experiments, or re-suspended in platelet-poor-plasma prepared from hirudin-coated tubes for platelet experiments requiring whole plasma. Hirudin-coated collection tubes were used to avoid chelation of Zn^2+^ by other agents (*e.g.* citrate or EDTA).

Platelet aggregation experiments were performed to assess the effect of Zn^2+^ and NEFAs (octanoate or myristate) on platelet aggregation. Solutions of ZnCl_2_ (10, 50 or 100 μM), sodium octanoate or sodium myristate (2 or 4 mol. eq.) and *N*,*N*,*N*′,*N*′-tetrakis(2-pyridinylmethyl)-1,2-ethanediamine (TPEN, 50 μM diluted in ethanol) were added to washed-platelets or platelets-in-plasma. Vehicle control experiments were also performed. Platelet aggregation was elicited with 2 μM of γ-thrombin (Merck, Watford, UK, final volume 200 μL). Absorbance was monitored at 430 nm every 55 s for 35 min using an Optima plate-reader (BMG Labtech, Ortenberg, Germany) while incubating the plate at 37 °C and shaking it in orbital mode. Data were recorded as a negative change in absorbance from baseline (0%) and expressed as a percentage of the maximum response (100%). Calibration of 100% aggregation was achieved using platelet-poor-plasma. From the recorded responses, a maximum aggregation response was obtained.

### 2.4. Clinical sample collection

A total of 54 patients with T2DM and 18 age-matched controls were recruited from Leeds Teaching Hospitals Trust. Inclusion criteria were: individuals with a known diagnosis of T2DM and aged between 18–75 years and already on aspirin therapy (given that aspirin may affect clot structure characteristics).^36^ Exclusion criteria included: any type of diabetes other than T2DM, any coagulation disorder, current or previous history of neoplastic disease, history of acute coronary syndrome or stroke within 3 months of enrolment, active history of transient ischemic attacks, history of deep venous thrombosis or pulmonary embolism, treatment with oral anticoagulant or non-steroidal anti-inflammatory drugs, abnormal liver function tests defined as alanine transferase >3 fold upper limit of normal, or previous or current history of gastrointestinal pathology. Baseline fasting blood samples were collected in trisodium citrate- or in lithium heparin-coated tubes. Plasma was separated within 2 h of collection by centrifugation at 2400 × *g* for 20 min at 4 °C, snap-frozen in liquid nitrogen and stored at −40 °C until analysis.

### 2.5. Turbidimetric fibrin clotting and lysis assays

Clot assays were performed as previously described,^37,38^ using a purified protein system, commercial pooled plasma from controls, as well as individual plasma samples from subjects with T2DM and age-matched controls.

For experiments in the purified system, sodium myristate (4 mol. eq.) was incubated for 15 min at 37 °C with HSA (in buffer). ZnCl_2_ was added to a final concentration of up to 100 μM Zn^2+^. Final concentrations in the purified proteins system were: 0.5 mg mL^−1^ fibrinogen (plasminogen-depleted, Merck), 100 μM HSA, 2.5 mM CaCl_2_, 0.05 U mL^−1^ thrombin, 39 ng mL^−1^ tissue plasminogen activator (tPA, Technoclone, Vienna, Austria) and 3.12 μg mL^−1^ plasminogen (Stratech, Ely, UK). The absorbance at 340 nm was read every 12 s at 37 °C using a Multiskan FC plate-reader (Thermo Scientific, Paisley, UK). Maximum absorbance, clot formation time (CFT; the time between the start of the assay and the time at which 50% of maximum clotting is reached) and lysis time (the time from maximum absorbance to the time at which the clotting has decreased to 50% of maximum) were calculated from the raw data.

For the experiments in commercial pooled plasma (First Link (UK) Ltd, Wolverhampton, UK), sodium myristate (4 mol. eq.) was incubated for 15 min at 37 °C with pooled plasma. ZnCl_2_ was added to Zn^2+^ concentrations up to 580 μM. Final concentrations were: plasma diluted 6-fold in buffer, 0–100 μM added Zn^2+^, 7.5 mM CaCl_2_, 0.03 U mL^−1^ thrombin and 20.8 ng mL^−1^ tPA. The absorbance at 340 nm was read every 12 s at 37 °C. Maximum absorbance, CFT and lysis time were calculated from the raw data.

For experiments in plasma from subjects with T2DM and controls, citrated plasma from those individuals was thawed and left for 15 min at 37°. ZnCl_2_ was added to a concentration of up to 580 μM Zn^2+^. For the clot formation assays, the final concentrations were: plasma diluted 3-fold in buffer, 40 or 200 μM added Zn^2+^, 7.5 mM CaCl_2_ and 0.03 U mL^−1^ thrombin. For the clot lysis assays, the final concentrations were: plasma diluted 6-fold in buffer, 20 or 100 μM added Zn^2+^, 3.75 mM CaCl_2_, 0.03 U mL^−1^ thrombin and 20.8 ng mL^−1^ tPA. The absorbance at 340 nm was read every 12 s at 37 °C. Maximum absorbance, CFT and lysis time were calculated from the raw data.

### 2.6. Speciation calculations

Zinc speciation models for the plasma used in platelet aggregation, and purified systems used in fibrin clotting assays included HSA sites A and B, and Tris; for pooled citrated plasma, the 1 : 1 and 1 : 2 citrate complexes of both Zn^2+^ and Ca^2+^ were also included. Stability constants (Table S1†) for the zinc complexes with sites A and B, determined in this work, were corrected for competition with 50 mM Tris at pH 7.4. Protonation constants and stability constants for complexes with citrate (1 : 1 and 1 : 2) and Tris were retrieved from the IUPAC Stability Constants Database (SCD),^39^ selecting studies with appropriate ionic strengths (0.1–0.15 M). The “Species” module distributed with the SCD was used for speciation modelling. Where appropriate, uncertainties are based on carrying out calculations at two different citrate concentrations that bracket the likely range present in citrated plasma. The concentrations used for the modelling are listed in Table S2.†

### 2.7. Scanning electron microscopy (SEM)

Clots were formed in duplicate from either: (1) 10 μM purified fibrinogen and 300 μM HSA in buffer in the presence and absence of 40 μM added Zn^2+^. (2) Pooled-plasma from 6 randomly chosen patients with T2DM and pooled-plasma from 6 age-matched controls in the presence and absence of 116 μM added Zn^2+^. Clots were prepared by diluting 1 : 1 in buffer either the purified system or the plasma and subsequently 45 μL of these diluted solutions were added to 5 μL of activation mix containing 2.5 U mL^−1^ thrombin and 25 mM CaCl_2_ in buffer. The clots were then processed by stepwise dehydration as previously described.^37^ All clots were viewed and photographed at ×5000, ×10 000, ×25 000 and ×30 000 magnification using a SU8230 scanning electron microscope (Hitachi, Maidenhead, UK) in 5 different areas. The diameter of 50 fibres in each image was measured using Adobe Photoshop (Adobe Systems, San Jose, CA). The mean diameter of the fibres in each image was used to compare each sample type.

### 2.8. Measurement of the plasma concentrations of total NEFA and other molecules

NEFAs were extracted from citrated plasma from subjects with T2DM and controls using Dole's protocol.^40^ NEFA concentrations were then measured using the Free Fatty Acid Assay Kit - Quantification (Abcam, Cambridge, UK). Zn^2+^ concentrations were measured in lithium-heparin plasma from subjects with T2DM and controls by inductively coupled plasma-mass spectrometry as described previously.^41^ HSA levels were measured in heparinized plasma with the bromocresol purple method using an automated analyser (Architect; Abbot Diagnosis, Maidenhead, UK). Plasma concentrations of fibrinogen, high density lipoprotein (HDL), low density lipoprotein (LDL), cholesterol, triglyceride, HbA1c, fasting glucose and platelets were measured with routine methods.

### 2.9. Measurement of the plasma concentrations of specific NEFA species by GC-MS

NEFAs were extracted with Dole's protocol,^40^ converted to fatty acid methyl esters (FAMEs), and characterized and quantified by gas chromatography-mass spectrometry (GC-MS). Citrated plasma from subjects with T2DM and controls was thawed and spiked with an internal standard fatty acid 17 : 0 (100 pmoles) to allow for normalization prior to extraction. The fatty acids were converted to FAMEs using 1500 μL of methanol, 200 μL of toluene and 300 μL of 8% HCl, followed by incubation for 5 hours at 45 °C. After cooling, samples were evaporated to dryness with nitrogen. The FAME products were extracted by partitioning between 500 μL of water and 500 μL of hexane and the samples were left to evaporate to dryness in a fume hood. The FAME products were dissolved in 30 μL dichloromethane and 1–2 μL was analysed by GC-MS on an Agilent Technologies (GC-6890N, MS detector-5973) with a ZB-5 column (30 m × 25 mm × 25 mm, Phenomenex), with a temperature program of: held at 70 °C for 10 min followed by a gradient to 220 °C at 5 °C min^−1^ and held at 220 °C for a further 15 min. Mass spectra were acquired from 50–500 amu and the identification of FAMEs was carried out by comparison of the retention times and fragmentation patterns with various FAME standard mixtures (Supelco, Poole, UK) as previously described.^42^

### 2.10. Data analysis and representation

Data are shown as mean ± standard deviation (SD). Graphs were generated and statistical analysis was performed using Prism 7.0 (GraphPad Software, La Jolla, CA). Differences between groups were analysed using multiple Student's *t*-tests or analysis of variance (ANOVA) with Dunnet's or Sidak's multiple comparisons tests. Correlations between linear variants were analysed with Pearson's correlation. Significance threshold: *p* ≤ 0.05.

## 3. Results and discussion

### 3.1. Influence of various NEFAs on Zn^2+^ binding to HSA

Using isothermal titration calorimetry (ITC), we previously demonstrated that myristate (14 : 0) impacts upon Zn^2+^ binding to HSA at the highest affinity Zn^2+^ site,^20^ whilst mutagenesis and X-ray crystallography studies confirmed this to be site A.^22^ We have also recently used offline size-exclusion-chromatography-ICP-MS to show that addition of 5 mol. eq. myristate reduced Zn^2+^-binding by BSA by 70%.^10^

However, myristate (C14 : 0) has a low abundance in plasma and on average a weaker affinity to serum albumins than the more abundant palmitate (C16 : 0) and stearate (C18 : 0),^33,43^ and therefore its quantitative effects may reflect the situation *in vivo* only in part. Although the overall affinities of NEFAs for HSA have been known for some time,^43–46^ it is in most cases unclear which binding constant refers to which of the 5 major binding sites, or whether different binding sites favour different NEFAs. It is known that site FA2 is one of the three high-affinity sites for palmitate,^47^ which together with stearate constitutes the bulk of saturated plasma NEFAs. Moreover, they are both elevated in T2DM.^29^ Owing to their physiological importance, direct assessment of the impact of these two NEFAs on Zn^2+^–HSA binding by competitive ITC experiments is required. In addition, we also included octanoate (8 : 0), laurate (12 : 0) and myristate (14 : 0) in the study to investigate the relevance of chain length. An X-ray structure in the presence of laurate is available (PDB 1E7F) showing that this NEFA can bind to site FA2 in a manner similar to other longer-chain NEFAs. However, structural studies on HSA^18^ as well as ITC experiments on BSA^10^ suggested that octanoate is too short to cause the conformational change necessary to disrupt Zn^2+^ binding, but this has not yet been assessed directly for HSA. Finally, palmitoleate (16 : 1-cis n9) was included in the experiment as a model mono-unsaturated NEFA. Mono- and polyunsaturated NEFAs may have beneficial effects in T2DM.^29^ Although oleate (18 : 1-cis n9) is more abundant, the shorter NEFA was chosen due to its better solubility.

**Fig. 2:**
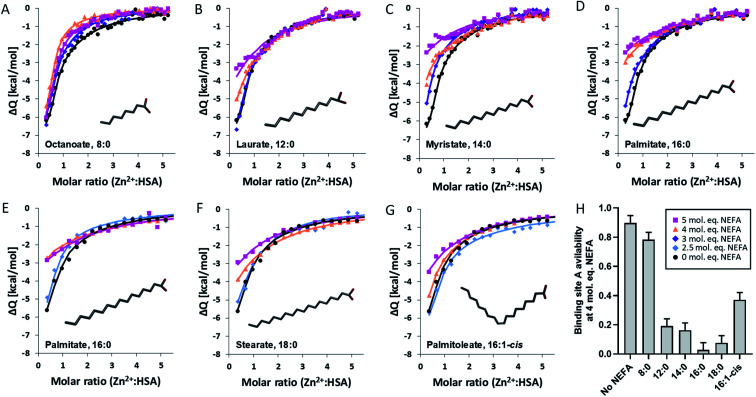
Binding of Zn^2+^ to HSA in presence of different NEFAs. ITC experiments were conducted in a buffer containing 50 mM Tris, 140 mM NaCl, pH 7.4. A first set of experiments was performed with 1.5 mM ZnCl_2_ titrated into 60 μM HSA, in the presence of either 0 (black), 2.5 (pale blue), 3 (dark blue), 4 (orange) or 5 (purple) mol. eq. of different NEFAs: (A) octanoate, (B) laurate, (C) myristate and (D) palmitate. The settings used were 25 °C, 39 injections (first injection was 0.4 μL, the remaining injections were 1 μL, initial delay 60 s and spacing 120 s. A second set of experiments was performed with 750 μM ZnCl_2_ titrated into 25 μM HSA, in the presence of (E) palmitate, (F) stearate and (G) palmitoleate. For those ITC experiments, the settings were adjusted to 25 °C, 19 injections (first injection was 0.4 μL, the remaining injections were 2 μL, initial delay 60 s and spacing 120 s. Each fit corresponds to a two-sets-of-sites model. (H) Bar chart representing the availability of binding site A in the presence of 4 mol. eq. of various NEFAs (the values for palmitate measured with 25 and 50 μM HSA were averaged) and the average for all values obtained in the absence of NEFA (both at 50 μM and 25 μM HSA). The data are represented as mean ± SD, with the SD obtained in the absence of NEFA used to estimate the other SD values. All NEFAs except octanoate perturbed Zn^2+^-binding to the protein, with the effect increasing with the concentration of NEFAs.

Building on our previous work,^18,20^ a “two-sets-of-sites” model was chosen to analyse all data. Fitted isotherms and a summary plot are shown in Fig. 2, raw data in Fig. S1–S26,† fitting parameters in Table S3† and fitting results in Tables S4 and S5.† In the absence of NEFA, Zn^2+^ bound to site A of HSA with a *K*_ITC_ of (3.1 ± 1.6) × 10^5^ M^−1^ (*n* = 4; Table S4†). This is in accord with our previous study which found a *K*_ITC_ of 1.35 × 10^5^ M^−1^.^20^ The titration curves acquired in presence of NEFAs were as previously fitted by fixing this binding constant as well as the associated enthalpy change (ΔH1) with the mean value calculated in the absence of NEFAs, whilst the respective stoichiometry was allowed to vary. This parameter reflects the NEFA-modulated zinc binding site availability. Binding constant and enthalpy for the second binding site were allowed to vary; these were not affected in a systematic manner by NEFAs (Table S5†). Addition of up to 5 mol. eq. of octanoate (Fig. 2A and H) had only a minor effect on Zn^2+^ binding to HSA. Octanoate is thought to be too short to elicit the switch,^10,18^ although no crystal structure of octanoate binding alone to HSA is available. Judging from the shapes of the curves, octanoate seems to modulate Zn^2+^ binding to HSA (Fig. 2A), but in a different manner to other NEFAs (Fig. 2B–G). Indeed, the quantitative effect on site A availability is small, and of the same magnitude as variations observed in the absence of NEFA (Fig. 2H). This result is broadly in accord with our previous size exclusion chromatography-ICP-MS study showing that it does not affect plasma zinc speciation.^10^

Much more dramatic changes were seen with laurate and longer chain saturated NEFAs, where the fitting results (Fig. 2 and Table S5†) suggested a reduction in the availability of site A with increasing NEFA concentration. A dependence on chain length was also evident: while 3 mol. eq. of laurate had little effect (Fig. 2B), 3 mol. eq. myristate or palmitate (Fig. 2C–E) reduced site A availability to about 50%. 2.5 mol. eq. of stearate had a similar effect (Fig. 2F). 4 mol. eq. NEFA reduced site A availability to 19% (laurate), 16% (myristate), or below 10% (palmitate and stearate). No Zn^2+^ binding was observed at site A in the presence of 5 mol. eq. of either of these four saturated NEFAs. Thus, our study of different NEFAs showed that their effect on Zn^2+^-binding by HSA is indeed broadly dependent on chain length, with only 2.5 mol. eq. of stearate sufficient to reduce site A availability below 50%. Most importantly, the physiologically more abundant longer-chain NEFAs palmitate and stearate are even more likely to alter plasma Zn^2+^ speciation and disrupt its buffering than previously predicted for myristate.^20^

The unsaturated NEFA palmitoleate (Fig. 2G) also led to reduced Zn^2+^ binding, but the effect was less severe than with palmitate at most concentrations examined, with the exception of 5 mol. eq. where no binding was detected for either NEFA. No X-ray crystallographic structures of palmitoleate bound to HSA are currently available to confirm that it binds at site FA2, but a structure of the closely related oleate (18 : 1 *cis*-n9; PDB 1GNI) shows that oleate binds in a similar extended conformation to stearate (18 : 0) at this site.^35^ In the same study, arachidonate (20 : 4) displayed reduced binding to FA2, hence it was concluded that unsaturated NEFAs bind HSA at the FA2 site as long as the degree of unsaturation is low, such as with oleate.^35^ Titrations with ^13^C-labelled oleate monitored by 2D NMR spectroscopy allowed inferring that FA2 remains one of the three high-affinity sites, at least for *cis*-n9 monounsaturated NEFAs.^48^ The reduced effect of palmitoleate (compared to that of palmitate) on zinc binding is consistent with the finding that unsaturated NEFAs display somewhat lower affinities than their saturated counterparts, an observation that has been partially attributed to differences in their solubility in water.^46^ Taking the trends for palmitate, stearate and palmitoleate together, we predict that the effect of oleate would be at least as pronounced as that of palmitoleate if not larger.

The differential effects of the various NEFAs studied are summarised in Fig. 2H, using site A availability values at 4 mol. eq. NEFA as an example. This illustrates that elevation of the physiologically highly abundant saturated NEFAs palmitate and stearate strongly impair Zn^2+^-binding to HSA, whilst the mono-unsaturated palmitoleate (and by inference the more abundant oleate) has a weaker effect. Although our ITC results concern single NEFAs, total NEFA concentrations in human plasma can reach or even exceed 4 mol. eq. with respect to HSA, especially in metabolic disorders such as T2DM.^26^ It would thus appear likely that the allosteric interaction of NEFAs and Zn^2+^ on HSA has the potential to impact on plasma Zn^2+^-handling *in vivo*. Effects on Zn^2+^-buffering may particularly manifest themselves in zinc-regulated processes such as coagulation. When platelets release Zn^2+^ during coagulation, HSA is likely to participate in buffering/controlling its action in the vicinity of injury sites; we hypothesise that the presence of excess saturated (and mono-unsaturated) NEFAs may hinder this regulatory action. Therefore, we looked next at the potential consequences of this change in zinc-buffering on the regulation of coagulation, using myristate as a model NEFA, as it is more soluble than palmitate or stearate while also offering a good approximation of their effects.

### 3.2. Effect of Zn^2+^ and NEFAs on γ-thrombin-induced platelet aggregation

**Fig. 3:**
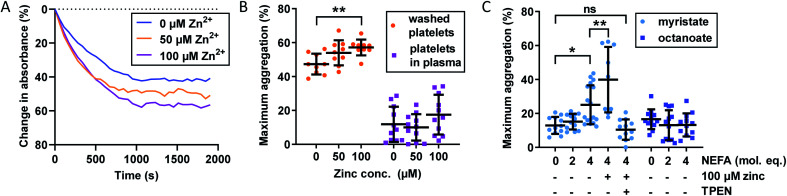
Effects of Zn^2+^ and NEFAs on maximum platelet aggregation in washed platelets and platelets re-suspended in plasma (containing the anticoagulant hirudin). (A) Representative raw data from platelet aggregation assays. Washed platelets, no myristate, 0–100 μM Zn^2+^. (B) Effect of Zn^2+^ on maximum platelet aggregation (*n* = 11). Maximum aggregation was higher in washed platelets than in platelets re-suspended in plasma. Addition of Zn^2+^ also increased maximum aggregation in washed platelets (*p* = 0.0080; one-way ANOVA) but not in platelets re-suspended in plasma. (C) Effect of NEFAs on platelet maximum aggregation in platelets-in-plasma (*n* = 12). Maximum aggregation increased with addition of myristate (*p* < 0.0001; one-way ANOVA) but not of octanoate. The presence of 4 mol. eq. myristate and 100 μM Zn^2+^ further increased maximum aggregation (*n* = 9, *p* = 0.0075; multiple comparison tests). The addition of the Zn^2+^ chelator TPEN reversed the effect of 4 molecular equivalents of myristate (*p* = 0.0032; multiple comparison tests compared to myristate alone). The data are represented as mean ± SD. Statistical significance is indicated by ns where *p* > 0.05, * where *p* < 0.05, ** where *p* < 0.01 and *** where *p* < 0.001.

The importance of Zn^2+^ for platelet aggregation is well established,^2^ with its effects on platelet behaviour exercised through both extracellular and intracellular interactions.^16^ We assessed the effect of Zn^2+^ and NEFAs on platelet aggregation in washed platelets and platelets-in-plasma. For the latter experiments, fibrin clotting was prevented during blood collection by the anticoagulant hirudin, as it has been shown to be the optimal anticoagulant to use during platelet aggregation studies.^49^ Aggregation was stimulated by addition of 2 μM γ-thrombin, which does not induce fibrin clotting (representative raw data in Fig. 3A and results in Fig. 3B and C). The effect of up to 100 μM total Zn^2+^ on platelet aggregation was studied. Although 100 μM Zn^2+^ may seem high (>6-times total basal exchangeable plasma Zn^2+^ concentration; *ca.* 15 μM (ref. 7)), it potentially reflects the concentration of Zn^2+^ in atherosclerotic plaques or in the vicinity of the platelet surface immediately after release,^16^ although it has remained difficult to establish accurate values for such transient elevations in Zn^2+^.

Addition of up to 100 μM total Zn^2+^ increased maximum aggregation in washed platelets in a concentration-dependent manner (*p* = 0.0080; one-way ANOVA). Remarkably though, the aggregation of platelets re-suspended in plasma was not enhanced by Zn^2+^ at this concentration, presumably due to the presence of Zn^2+^-binding proteins such as HSA. The plasma concentration of the latter (>600 μM) greatly exceeds that of our highest Zn^2+^ concentration and hence is sufficient to provide an efficient Zn^2+^ buffer that, according to speciation modelling (Table S2†), keeps non-HSA bound Zn^2+^ below 0.3 μM, apparently too low to enhance platelet aggregation. We also note that the maximum aggregation was generally higher in washed-platelet than in platelets-in-plasma, most likely due to the presence of other molecules that attenuate platelet aggregation in plasma. Next, the effect of NEFAs in plasma was investigated. Addition of up to 4 mol. eq. octanoate to platelets-in-plasma had no effect on maximum aggregation, suggesting that there is neither direct nor indirect effect of this NEFA. In contrast, addition of 4 mol. eq. of myristate alone led to a clear increase in aggregation (*p* < 0.0001; one-way ANOVA). Addition of 100 μM Zn^2+^ (0.17 mol. eq. compared to HSA) in combination with 4 mol. eq. myristate further increased maximum aggregation (*p* = 0.0075; multiple comparison tests) - notably in contrast to 4 mol. eq. myristate alone. Finally, the metal chelator *N*,*N*,*N*′,*N*′-tetrakis(2-pyridinylmethyl)-1,2-ethanediamine (TPEN) abolished the effects of and myristate + Zn^2+^, with maximum aggregation reverting to the same level as in the control. This significant observation is in full agreement with the notion that the effect of myristate in plasma was mediated by an increase in free Zn^2+^, as a consequence of the impairment of Zn^2+^ binding by HSA, as observed in the ITC experiments shown in Fig. 2. The lack of effect for octanoate – which can bind to HSA without markedly impairing Zn^2+^ binding – further emphasises that the NEFA effect was likely mediated through the allosteric switch on HSA.^21^ The fact that addition of myristate alone increased platelet aggregation indicates that the switch may operate already at Zn^2+^ levels intrinsically present in plasma (with 4 mol. eq. raising non-HSA-bound Zn^2+^ from an estimated 40 nM to a minimum of 0.49 μM), but is potentiated after addition of 100 μM total Zn^2+^, corresponding to an estimated ∼10 μM non-HSA bound Zn^2+^ (speciation modelling parameters in Table S2†). Next, we explored the impact of combined Zn^2+^ and NEFAs on fibrin-dependent clotting.

### 3.3. Effect of Zn^2+^ and NEFAs on fibrin clots

The effect of Zn^2+^ on fibrin clot characteristics was previously examined in two separate studies by Henderson *et al.* using either a purified protein system or dialysed plasma.^3,4^ Their studies found that addition of Zn^2+^ (up to 6 or 15 μM added “free” Zn^2+^, which required adding *ca.* 100-fold higher total Zn^2+^ due to chelation by tricine) accelerated fibrin polymerisation and clot formation, overall resulting in thicker fibrin fibres and both increased maximum absorbance and clot porosity. The altered clot structure also led to enhanced clot lysis by allowing increased flow of plasma components inside the clot. However, the same studies also showed that Zn^2+^ reduced plasminogen activation, which delays clot lysis. Both effects together resulted in prolonged lysis times. Thus, Zn^2+^ has several regulatory effects on fibrin clotting and lysis. Therefore, elevated NEFA concentrations might impact both fibrin clotting and lysis by diminishing Zn^2+^-binding to HSA.

To assess whether this is indeed the case, we utilised a validated turbidimetric assay employing plasma samples pooled from healthy individuals as well as a purified protein system (containing fibrinogen and HSA). Clot formation was initiated by addition of thrombin and Ca^2+^ (which together convert fibrinogen into fibrin), and clot lysis was induced by addition of plasminogen (only in the purified system, this was not added to plasma) and tPA (tissue plasminogen activator, which converts plasminogen into plasmin, which in turn lyses the fibrin network). The effect of Zn^2+^ on clot formation and on clot lysis was followed by measuring the absorbance at 340 nm (representative raw data are shown in Fig. S27†). Maximum absorbance, clot formation time (CFT; the time between the start of the assay and the time at which 50% of maximum clotting is reached) and lysis time (the time from maximum absorbance to the time at which clotting has decreased to 50% of maximum) were determined. Maximum absorbance reflects fibre thickness of clots made from purified proteins, while in plasma clots it gives information on both fibre thickness and clot density (denser plasma clots are difficult to break down and therefore more likely to cause vascular occlusion). Shorter CFTs indicate a higher tendency of plasma to clot, while prolonged lysis times are considered to be associated with higher pro-thrombotic risk as the clot stays longer in plasma and can move to occlude a blood vessel before it is degraded.

**Fig. 4:**
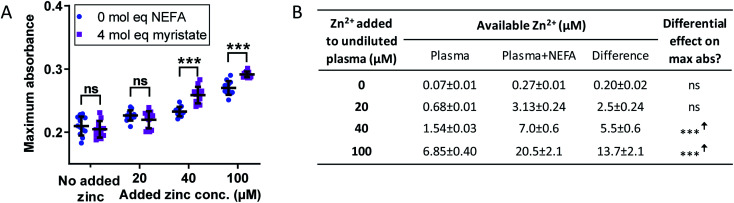
(A) Effects of Zn^2+^ and NEFAs on fibrin clot parameters in pooled plasma on maximum absorbance. Turbidimetric fibrin clotting and lysis assays were performed in pooled plasma diluted 6-fold in buffer (50 mM Tris, 100 mM NaCl, pH 7.4), with final concentrations of 7.5 mM CaCl_2_, 0.03 U mL^−1^ thrombin, 20.8 ng mL^−1^ tPA, while 0–100 μM added Zn^2+^, as well as either 0 or 4 mol. eq. myristate relative to HSA concentration (*n* = 12). Two-way ANOVA followed by Sidak's multiple comparisons tests was used to analyse the data. Maximum absorbance was significantly increased in pooled plasma in the presence of Zn^2+^ (*p* < 0.0001) and in the presence of 4 mol. eq. myristate (*p* = 0.034). (B) Estimates for available Zn^2+^ in pooled plasma in presence and absence of 4 mol. eq. added myristate. “Available Zn^2+^” was taken as the sum of free- and Tris-bound Zn^2+^. Citrate has been suggested to mask the effects of Zn^2+^ in coagulation;^1^ therefore it was assumed that citrate-bound zinc would not be available to influence clotting or clot lysis. The entries in columns 5 refer to the data plotted in Fig. S28E.† The data are represented as mean ± SD. Statistical significance is indicated by ns where *p* > 0.05, * where *p* < 0.05, ** where *p* < 0.01 and *** where *p* < 0.001.

The effect of Zn^2+^ and NEFAs was examined, separately and together, on fibrin clot properties in human plasma. In these experiments, citrated pooled plasma from healthy individuals was used. Contrary to previous studies performed in plasma, we chose not to dialyse the plasma to remove the citrate, as this can result in a loss of other small (yet important) molecules. We have also previously established that fluorescent dyes such as FluoZin3 cannot reliably detect available Zn^2+^ in systems containing albumin.^10–12^ Therefore, to account for the Zn^2+^-buffering capacity of citrate, a simple speciation model was set up incorporating the two highest zinc affinity sites on HSA, Tris and citrate (Fig. 4B and S28F†) to calculate non-HSA bound Zn^2+^, as well as how much Zn^2+^ would be liberated from site A of HSA following NEFA binding (using data from Fig. 2). These estimates indicated that addition of up to 100 μM Zn^2+^ to 6-fold diluted plasma corresponds to concentrations of up to 6.85 μM available Zn^2+^, well within limits of what is thought to become available in the proximity of activated platelets.^16^ In addition, the model showed that between 0.2 and 13.7 μM Zn^2+^ would additionally become available upon NEFA addition to HSA (Fig. 4B). These values are likely over-estimates, since the models did by necessity not include the buffering capacity of the plethora of other proteins with zinc-binding ability,^10^ as affinity data for these are not yet available. We then performed the turbidimetric clot assay (Fig. 4A and S28A and B†). In the absence of NEFA, addition of Zn^2+^ increased both maximum absorbance and CFT. Addition of 20 μM Zn^2+^ increased lysis time, whilst addition of 40 and 100 μM added Zn^2+^ decreased lysis time. Zn^2+^ significantly affected all three parameters (*p* < 0.0001 for all; two-way ANOVA).

We then assessed the effect of 4 mol. eq. of myristate alone in the plasma system. Addition of myristate alone decreased CFT (Fig. S28A†) and increased lysis time (Fig. S28B†) (*p* < 0.0001 and *p* = 0.0002 respectively; Sidak's multiple comparison tests), whilst maximum absorbance was not affected. *A priori*, the effects of myristate in plasma may either be mediated by acting on endogenous HSA-bound Zn^2+^, or correspond to a direct effect of myristate on clotting. In order to explore whether the presence of endogenous Zn^2+^ may explain the effects of NEFA addition alone, we carried out analogous experiments in a purified protein system. Our system contained 100 μM HSA, whilst total Zn^2+^ concentrations were varied between 0 and 100 μM. The same speciation model as previously was used (but not including citrate; Table S2 and Fig. S29G†). Non-HSA bound Zn^2+^ was estimated to range from 0.75 μM (20 μM added Zn^2+^) to 13 μM (100 μM added Zn^2+^) in the absence of NEFAs, and from 6.0 μM (20 μM added Zn^2+^) to 59.2 μM (100 mM added Zn^2+^), in the presence of 4 mol. eq. myristate. We note that the clots formed in this system were much less dense (lower maximum absorbance) than those formed in plasma, with faster clot formation and lysis times, likely due to the absence of numerous plasma proteins influencing coagulation. We confirmed that all three parameters were affected by Zn^2+^ also in this system (Fig. S29A–C†). Addition of 4 mol. eq. of myristate alone (with no Zn^2+^ added) also increased maximum absorbance and CFT (*p* = 0.0046 and *p* = 0.0060 respectively; Sidak's multiple comparison tests), suggesting a direct effect on these parameters, whereas lysis time was not affected. Similar direct effects of NEFAs on fibrin clotting have been previously reported for a purified system that did not include HSA: addition of <800 μM stearate increased maximum absorbance and CFT through modulation of thrombin activity and fibrin assembly, likely *via* a fibrin(ogen)-thrombin-NEFA complex.^30^ Apparently, this action may occur even in the presence of HSA. Thus, although we did not observe a corresponding effect on maximum absorbance in our pooled plasma system, we cannot rule out direct effects of myristate on clotting parameters.

Therefore, in order to separate the effect on fibrin clotting of myristate alone from the effect of myristate in conjunction with Zn^2+^ and HSA, the values of the fibrin clot parameters were calculated relative to “no Zn^2+^ added” (with and without NEFA; Fig. S28C–E and S29D–F†). In the plasma system, it was found that the effects of addition of Zn^2+^ on maximum absorbance, CFT and lysis time were overall more pronounced in the presence of myristate. Maximum absorbance was unchanged at 20 μM Zn^2+^ and increased by 2.5-fold at 40 μM Zn^2+^ and 1.5-fold at 100 μM Zn^2+^; CFT was increased by 31 s at 20 μM Zn^2+^, 74 s at 40 μM Zn^2+^ and 79 s at 100 μM Zn^2+^; lysis time was decreased by 537 s at 20 μM Zn^2+^, 868 s at 40 μM Zn^2+^ and 513 s at 100 μM Zn^2+^. When all data points from the differential plots (Fig. S28C–E†) are analysed together, addition of myristate significantly affected maximum absorbance, CFT and lysis time (*p* = 0.0013, *p* < 0.0001 and *p* < 0.0001, respectively; two-way ANOVA over all data points). The data summarised in Fig. 4B and S28F† indicate that myristate needs to “effectively displace” between 2.5 and 5.5 μM Zn^2+^ to significantly affect maximum absorbance, whereas CFT and lysis time were sensitive to smaller changes in available zinc.

In summary, although some trends were complex, both in the purified system and in plasma, the addition of Zn^2+^ in the presence of 4 mol. eq. myristate resulted in more pronounced changes in maximum absorbance, CFT and lysis time in pooled plasma. This shows that, independently from their direct effect, NEFAs influence buffering of Zn^2+^ by HSA to consequently alter Zn^2+^ speciation in plasma in a manner that influences fibrin clot parameters. It is important to note that the simultaneous presence of Zn^2+^ and NEFAs causes an increase in CFT, which was associated with a decrease in lysis time. This would potentially lead to an anti-thrombotic effect. Also, the presence of Zn^2+^ and NEFAs increases maximum absorbance. This could give rise to a pro-thrombotic effect (if due to denser clots) or an anti-thrombotic effect (if due to increased fibrin fibre thickness). The overall effect is difficult to assess but it is clear that the simultaneous presence of Zn^2+^ and NEFAs alter fibrin clotting.

### 3.4. Differences in clot formation in plasma from subjects with T2DM and controls

**Fig. 5:**
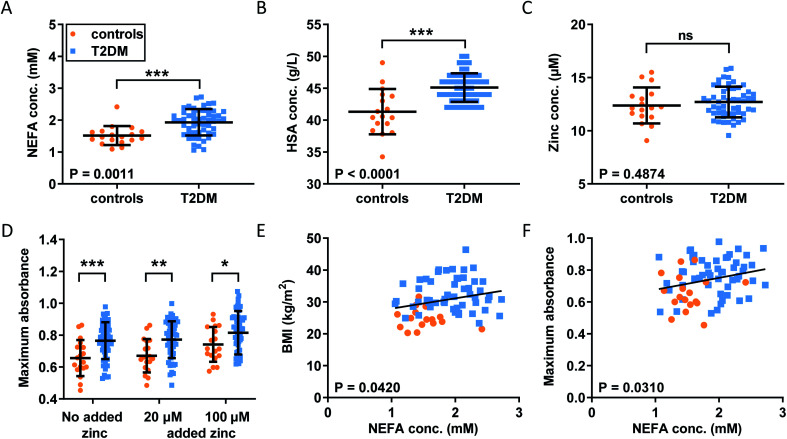
Comparison of the plasma concentrations of NEFA, HSA and zinc and of the clot formation and lysis parameters in plasma samples from patient with T2DM and from controls. Comparison of (A) plasma NEFA concentration, (B) HSA concentration and (C) total plasma zinc concentration in the subjects with T2DM and the controls; the data were analysed with *t*-tests. Turbidimetric fibrin clotting and lysis assays were performed in plasma samples with final concentrations of plasma diluted 3-fold in buffer, 7.5 mM CaCl_2_, 0.03 U mL^−1^ thrombin and 0, 20 or 100 μM added ZnCl_2_ (*n* = 54 for diabetes subjects and *n* = 18 for controls). (D) Maximum absorbance was obtained from these experiments. The data are represented as mean ± SD. Two-way ANOVA followed by Sidak's multiple comparison tests was used to analyse the data. Maximum absorbance increased significantly in T2DM subjects compared to controls (*p* = 0.0007), as well as in the presence of Zn^2+^ (*p* < 0.0001). Relationships between fibrin clot parameters and NEFA concentration were assessed with Pearson's correlation: (E) positive correlation between BMI and NEFA levels (*p* = 0.0420); (F) positive correlation between maximum absorbance and NEFA levels (*p* = 0.0310). Statistical significance is indicated with ns where *p* > 0.05, * where *p* < 0.05, ** where *p* < 0.01 and *** where *p* < 0.001.

Our observations in model systems prompted us to investigate whether the NEFA-induced alteration of zinc speciation might have clinical relevance. Plasma levels of NEFAs are elevated in T2DM,^26,50^ and thrombotic complications are a frequent co-morbidity of this disease.^51,52^ In addition, elevated serum NEFA levels in individuals taking part in the Cardiovascular Health Study have been associated with a higher risk of incident hospitalisation for all causes and for diabetes in particular.^53^ We analysed plasma samples taken from 54 individuals with T2DM and 18 controls after fasting (as is common practice for measuring lipids, as food intake affects lipid metabolism).^29^ In each sample, NEFA, total zinc and HSA concentrations were measured (Fig. 5A–C). Demographic information and plasma concentrations of lipids and glucose for the two groups are presented in Table S6.† The groups were matched for age but not sex (no sex difference in NEFA, zinc, HSA, fibrinogen concentration or in the platelet count was found, Fig. S30†). The T2DM group had higher BMI (*p* < 0.0001; all significance testing in the following were done by *t*-tests if not stated otherwise), higher concentrations of HbA1c (*p* < 0.0001), plasma glucose (*p* < 0.0001) and triglycerides (*p* = 0.0313), and a higher cholesterol/LDL ratio (*p* = 0.0198), but had lower concentrations of cholesterol, HDL and LDL (*p* < 0.0001 for all). As previously found, the T2DM group had significantly higher total plasma NEFA concentrations (*p* = 0.0011) than controls (Fig. 5A), and this correlated with BMI (Fig. 5E; *p* = 0.0420; Pearson). The total NEFA concentrations in plasma from subjects with T2DM averaged 1.9 mM (3.2 mol. eq. to HSA) and were as high as 2.7 mM (4.5 mol. eq. to HSA) in some cases, whereas those from controls were significantly lower (average 1.5 mM, 2.5 mol. eq. to HSA). Although HSA concentrations were also significantly higher in T2DM patients (683 μM *vs.* 626 μM; *p* < 0.0001), 1.9 mM amounts to *ca.* 3 mol. eq. of total NEFA relative to HSA concentration, and 2.7 mM corresponds to *ca*. 4 mol. eq. Our ITC experiments showed a reduction in the Zn^2+^-binding capacity of HSA of around 50% for 3 mol. eq. of palmitate, and reductions of at least 90% for 4 mol. eq. of stearate or palmitate (Fig. 2H). Thus, strong effects on the Zn^2+^-binding capacity of HSA can be expected from the concentrations found in T2DM patients. The difference in HSA concentrations between groups may be due to a slight dehydration in T2DM patients. We measured urea concentrations as a proxy for dehydration but did not find a difference, however this is probably due to the size of our cohort (Table S6†). Fibrinogen concentrations and the platelet counts were comparable between groups, and no difference in total zinc concentration was observed between patients and controls, contrary to some previous reports documenting a small decrease in total plasma zinc in individuals with T2DM.^54^ Even when separating the samples by sexes, no difference could be seen between male or female patients and their respective same-sex controls (Fig. S30†). Nevertheless, the lack of difference in zinc concentrations is probably due to the size of our cohort.

With this basic information in hand, the clotting parameters of plasma for each individual were assessed as before. Turbidimetric assays were performed on all samples with and without 20 or 100 μM added Zn^2+^ (final concentrations), as shown in Fig. 5D and S31.† Maximum absorbance was higher in the T2DM group compared to controls, regardless of the presence or absence of additional Zn^2+^ (Fig. 5D; *p* = 0.0007; two-way ANOVA). In addition, maximum absorbance correlated with total NEFA concentration (Fig. 5F; *p* = 0.03010; Pearson). Globally, lysis time was prolonged in T2DM subjects compared to controls (*p* = 0.0258; two-way ANOVA), but multiple comparison tests showed that comparisons between controls and T2DM at “no added zinc”, “20 μM added zinc” or “100 μM added zinc” individually were not significant (Sidak's multiple comparison tests). No clear trends were seen for CFT, and neither CFT nor lysis time was correlated with NEFA concentration. Since a previous study had found that maximum absorbance is higher in females with T2DM, we assessed whether we had any sexual difference in fibrin clot parameters in our cohort. Comparisons of maximum absorbance, CFT and lysis time between sexes revealed no significant differences (Fig. S32†).^55^ To summarise, a higher maximum absorbance in T2DM and its correlation with NEFA concentration parallel the trend observed upon addition of myristate in pooled plasma (Fig. 4). Although the increases in lysis time were not statistically significant, elevated clot densities found in T2DM likely increase the risk of thrombosis. Those correlations with NEFAs are likely partly due to an effect of NEFA alone on clotting, but our previous clotting experiments suggest that the effect of NEFAs on Zn^2+^-buffering by HSA is also likely involved.

### 3.5. Differences in fibrin clot fibre thickness between subjects with T2DM and controls

The main parameter influencing maximum absorbance is clot density, however it can also be affected by a difference in clot ultrastructure, such as fibrin fibre thickness or clot porosity. Thin fibres have been associated with a decreased rate of conversion of plasminogen into plasmin by tPA, which results in slower fibrin clot lysis and would thus have a pro-thrombotic effect.^56^ Lower fibrin fibre density and a higher clot porosity promote clot lysis by allowing an increased flow of plasma components inside the clot. NEFAs also have a direct effect on clot ultrastructure, although this depends on the type of NEFA: in a purified protein system with fibrinogen but no HSA, 50–500 μM stearate has been shown to increase fibre thickness, while 40–400 μM oleate reduced it.^30^ In addition to affecting the kinetics of fibrin clot formation and clot density, Zn^2+^ also alters clot ultrastructure in ways that can make clots more or less pro-thrombotic. Previous studies by Henderson *et al.* carried out in a purified system without HSA showed that addition of Zn^2+^ increased fibrin fibre thickness and clot porosity.^3^ Those are anti-thrombotic effects, yet those studies have also shown that – because Zn^2+^ simultaneously reduces plasminogen activation – the net result was a prolonged lysis time and thus pro-thrombotic.

**Fig. 6:**
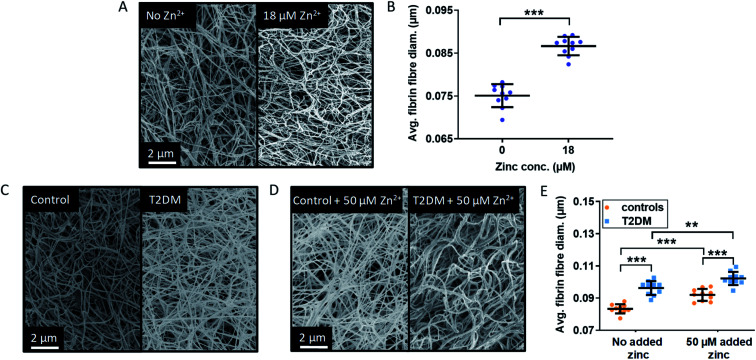
Effects of Zn^2+^ on fibrin fibre thickness in a purified system and comparison of the fibrin fibre thickness in plasma samples from patient with T2DM and controls. (A) Represented images of fibrin fibres from SEM experiments using a purified system with final concentrations of 4.5 μM fibrinogen, 135 μM HSA, 2.5 mM CaCl_2_, 0.25 U mL^−1^ thrombin and either 0 or 18 μM ZnCl_2_ (duplicates of clot, 5 images per samples, 50 fibres measured per image). (B) Comparison of the fibrin fibre diameter in clots formed from a purified system. Addition of Zn^2+^ significantly increased fibrin fibre diameter (*p* < 0.0001). (C) and (D) SEM images of clots produced from pooled-plasma samples (*n* = 6 each, 22.5 μL plasma diluted in buffer to 50 μL) from subjects with T2DM and controls with final concentrations of: 2.5 mM CaCl_2_, 0.25 U mL^−1^ thrombin and either 0 or 50 μM added ZnCl_2_ (duplicates of clot, 5 images per sample, 50 fibres measured per image). Representative pictures taken (C) in the absence of Zn^2+^ and (D) in the presence of 50 μM added Zn^2+^. (E) Comparison of fibrin fibre diameter in clots formed from plasma taken from subjects with T2DM and controls. Two-way ANOVA followed by Sidak's multiple comparison tests was used to analyse the data. Addition of Zn^2+^ significantly increased fibrin fibre diameter and this effect was exacerbated in the presence of T2DM (*p* < 0.0001 for both). The data are represented as mean ± SD. Statistical significance is indicated with ns where *p* > 0.05, * where *p* < 0.05, ** where *p* < 0.01 and *** where *p* < 0.001.

In order to assess whether the presence of HSA influences the effect of Zn^2+^ on clot ultrastructure in a purified protein system, fibrin fibre thickness was examined using scanning electron microscopy (SEM). Addition of 18 μM Zn^2+^ (40 μM Zn^2+^ added to the buffer system before dilution to form the clot) significantly increased fibrin fibre diameter (*p* < 0.0001; one-way ANOVA; Fig. 6A and B, S33 and S34†). This shows that the proportion of Zn^2+^ not bound to HSA in the absence of NEFA (estimated by speciation calculations to be *ca.* 0.47 μM in this system) is sufficient to alter fibrin fibre thickness.

SEM studies were then performed to examine fibrin fibre thickness in the T2DM group and the control group (Fig. 6C and D and S35–S38†); 6 plasma samples each from the control group and the T2DM group were randomly selected and pooled to form duplicate clots for each type of sample, in the absence and presence of 50 μM added Zn^2+^ (final concentration). For each clot, 5 images were taken, leading to 10 data points for each type of sample. Fibres were significantly thicker in the T2DM group compared to controls (*p* < 0.0001; two-way ANOVA). Besides clot density, this is likely to contribute to the higher maximum absorbance observed in T2DM (Fig. 5). Thicker fibres have previously been associated with increased clot porosity and faster lysis time, but the latter effect was not observed in our T2DM samples (Fig. S31†). Thus, the thicker fibrin fibres we observed may not be enough to compensate for the presumably higher density of fibrin clot, preventing an adequate circulation of lysis molecules inside the clot.

In our T2DM cohort, neither total fibrinogen nor total Zn^2+^ differed between groups. However, since isolating fibrinogen alone from T2DM patients produced different clots,^57^ it is difficult to say whether the thicker fibres we observe were caused by altered fibrinogen, a direct effect of NEFAs or higher levels of available Zn^2+^. Nevertheless, since experiments in purified systems (both ours and those from Henderson *et al.*)^3^ showed that Zn^2+^ alone makes fibres thicker, it is likely that the interference of NEFA on Zn^2+^-buffering by HSA contributes to this complex dynamic. This is qualitatively borne out by the observation that fibres from T2DM patients are at least as thick as fibres from controls with 50 μM added Zn^2+^.

### 3.6. Differences in plasma concentrations of specific NEFA species and associations with fibrin clot parameters

**Fig. 7:**
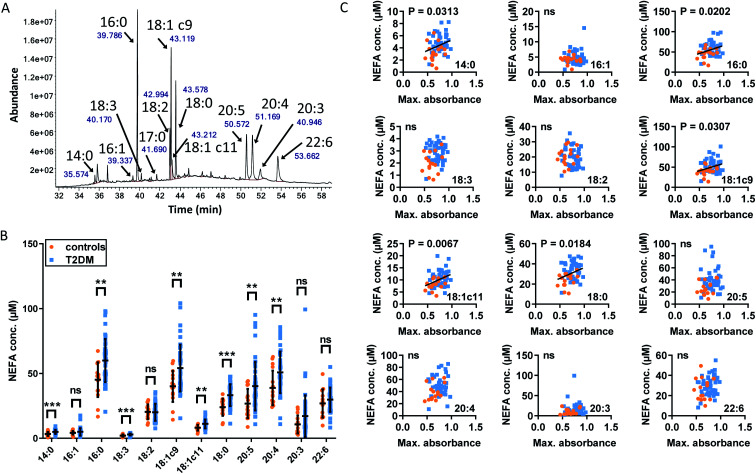
Comparison of the plasma concentrations of major NEFA species in samples from patients with T2DM and controls and associations with fibrin clot parameters. (A) Example of typical GC-MS chromatogram showing FAME separation. (B) Comparison of plasma concentrations of NEFAs between subjects with T2DM and controls. The data are represented as mean ± SD. Statistical significance is indicated with ns where *p* > 0.05, * where *p* < 0.05, ** where *p* < 0.01 and *** where *p* < 0.001. Plasma concentrations of myristate (14 : 0), palmitate (16 : 0), linolenate (18 : 3), oleate (18 : 1c9), *cis*-vaccenate (18 : c11), stearate (18 : 0), eicosapentaenoate (20 : 5) and arachidonate (20 : 4) were elevated in individuals with T2DM (*p* = 0.0010, *p* = 0.0023, *p* = 0.0003, *p* = 0.0054, *p* = 0.0029, *p* = 0.0002, *p* = 0.0092 and *p* = 0.0099 respectively). Concentrations of palmitoleate (16 : 1), linoleate (18 : 2), dihomo-γ-linoleate (20 : 3) and docosahexaenoate (22 : 6) were unchanged. (C) Relationships between maximum absorbance and the plasma concentrations of the major NEFA species. Plasma concentration of myristate, palmitate, oleate, *cis*-vaccenate and stearate positively correlated with maximum absorbance (*p* = 0.0313, *p* = 0.0202, *p* = 0.0307, *p* = 0.0067 and *p* = 0.0184 respectively, Pearson's).

So far, we have shown that Zn^2+^ affects several aspects of fibrin clotting in purified and complex systems, and that there are strong indications that elevated plasma NEFAs increase maximum absorbance of fibrin clots at least in part *via* a decrease in HSA-buffered Zn^2+^. Our ITC experiments looked at each NEFA in isolation while complex mixtures of different NEFAs are observed *in vivo*. It was therefore of interest to explore whether different NEFAs are associated with differences in fibrin clot maximum absorbance. To this end, the concentrations of major NEFA species in each T2DM and control plasma sample were measured using GC-MS (Fig. 7). The majority of NEFAs measured had an elevated concentration in subjects with T2DM compared to controls (*p* = 0.0010 for myristate, *p* = 0.0023 for palmitate, *p* = 0.0003 for linolenate (18 : 3), *p* = 0.0054 for oleate (18 : 1c9), *p* = 0.0029 for *cis*-vaccenate (18 : 1c11), *p* = 0.0002 for stearate, *p* = 0.0092 for eicosapentaenoate (20 : 5) and *p* = 0.0099 for arachidonate (20 : 4); *t*-tests), with the exception of palmitoleate (16 : 1), linoleate (18 : 2), dihomo-γ-linoleate (20 : 3) and docosahexaenoate (22 : 6). The association between plasma concentrations of those NEFAs and fibrin clot maximum absorbance was then examined across all samples. The concentrations of the saturated NEFAs myristate, palmitate and stearate and the mono-unsaturated NEFAs oleate and *cis*-vaccenate positively correlated with maximum absorbance (Fig. 7C). Although the concentrations of myristate (*ca*. 4.9 μM in T2DM, 3.3 μM in controls) and *cis*-vaccenate (*ca*. 11.1 μM in T2DM, 8.1 μM in controls) are low and do not change significantly when comparing across all individuals (Fig. 7B), it is remarkable that their levels showed a correlation with maximum absorbance. In turn, not all NEFAs that were significantly elevated in T2DM patients correlated with clot maximum absorbance, with the poly-unsaturated 18 : 3 (linolenate), C20 : 4 (arachidonate) and 20 : 5 (eicosapentaenoate) being notable exceptions.

What do the NEFAs that show an association with this clotting parameter have in common? Firstly, the three saturated and two mono-unsaturated NEFAs are all considered to be primary products of *de novo* lipogenesis.^58^ There are indications that plasma NEFAs resulting from this process are most strongly associated with diabetes incidence.^29^ Secondly, all five have either been shown to, or can be predicted to, bind to the NEFA binding site FA2 with high affinity. Our ITC experiments have demonstrated a direct effect on Zn^2+^ binding for the three saturated NEFAs, and although oleate and *cis*-vaccenate were not examined due to their very low solubility, it can be estimated based on published affinity data that their effects would be at least as pronounced as those of the shorter palmitoleate.^33,35^ In contrast, the poly-unsaturated NEFAs that were elevated in T2DM subjects did not show an association with clotting. Binding patterns on HSA for polyunsaturated NEFAs such as arachidonate are known to differ considerably from those of other NEFAs,^35,46^ and it may be suggested that their effect on Zn^2+^-buffering by HSA might therefore be smaller. This would be consistent with the absence of a correlation between their concentration and maximum absorbance in the clotting assay.

Due to the complexity of clinical samples, we can at this point not exclude some unknown factor or mechanism that would also explain the associations shown in Fig. 7, but we observe that they are all consistent with our original hypothesis, namely that particular plasma NEFAs induce changes in the speciation of plasma Zn^2+^ and hence may influence Zn^2+^-dependent clotting. It is interesting to see that there is a partial overlap between NEFAs with the strongest correlations with T2DM risk (saturated NEFAs) and those that are associated with abnormal clotting, although it would be premature to suggest that zinc mishandling could be one of the important physiological processes that link both observations.

## 4. Conclusions

**Fig. 8:**
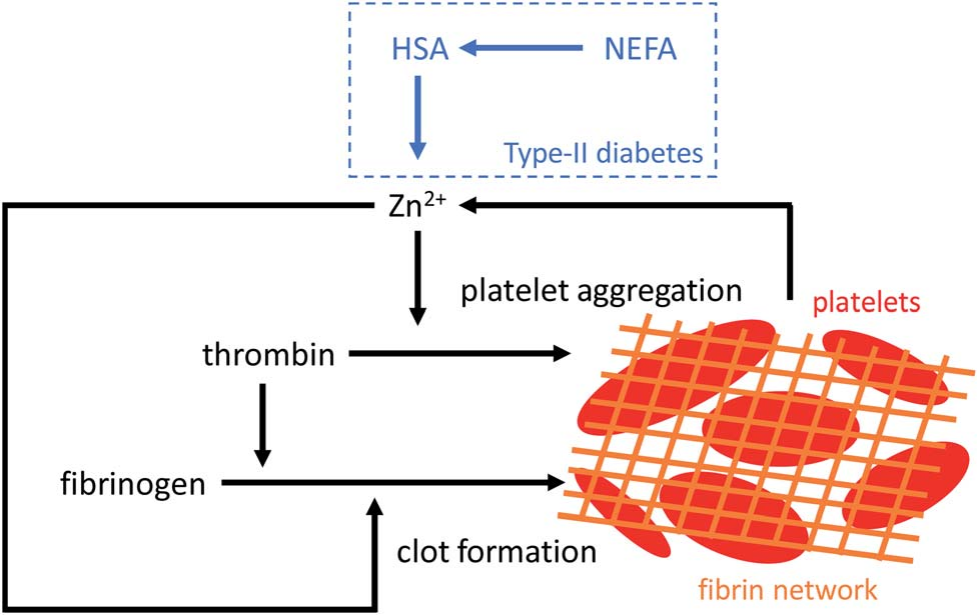
Zn^2+^ increases platelet aggregation and fibrin clot formation. Platelet activation during coagulation triggers the release of Zn^2+^. In addition, NEFA binding to HSA disrupts Zn^2+^ binding at site A. This will likely increase the available plasma Zn^2+^ concentration resulting in an increase in platelet aggregation and influencing fibrin clot formation.

The differences observed in our clinical cohort are likely to have substantial long-term consequences, leading to an altered coagulation process in individuals with T2DM. The effects of NEFAs and Zn^2+^ on coagulation demonstrated in this study are summarized in Fig. 8. Even a moderate presence of palmitate or stearate (2.5 mol. eq.; *ca.* 1.5 mM) markedly affected zinc site A availability (Fig. 2 and Table S5†), whilst 4 mol. eq. (*ca.* 2.4 mM; a level for total NEFA observed in some of the individuals included in this study) of either of these NEFAs reduced availability to below 10%. The fraction of Zn^2+^ not bound to HSA can be calculated by simple speciation modelling using our ITC results. The presence of 4 mol. eq. myristate will increase non-HSA-bound Zn^2+^ by no more than 0.5 μM in a non-coagulatory state. However, during coagulation platelets will release an unknown (but likely mid-to-high micromolar) concentration of Zn^2+^ at the injury site. We have estimated the concentration of Zn^2+^ that becomes available under those circumstances (Fig. S39†), showing that a rise in total local Zn^2+^ concentration of up to 300 μM at the surface of activated platelets would result in up to 14 times more (an additional 16 μM) Zn^2+^ becoming available for binding with other proteins, including those involved in coagulation. We have shown that concentrations well below this range are sufficient to increase fibrin clot maximum absorbance. Furthermore, we have shown that zinc and NEFAs synergistically promote platelet aggregation. It is thus likely that the elevated NEFA levels found in T2DM participate in the alterations of the coagulation process associated with this disease.

We have demonstrated in T2DM patients that plasma NEFA levels are sufficiently elevated such as to alter plasma zinc speciation and have shown the impact of additional non-HSA bound Zn^2+^ binding to coagulation proteins on clot processes. However, while our T2DM group had the same total plasma zinc concentration as our control group, in other studies individuals with T2DM have been reported to be mildly deficient in zinc.^54^ This could be due to chronically impaired binding and transport of zinc by HSA, resulting in altered zinc distribution, cellular absorption and/or partial renal clearance of the “excess” zinc. However, it is important to point out that a somewhat lower total plasma zinc concentration does not preclude binding of a higher proportion of Zn^2+^ to coagulation proteins leading to dysregulated coagulation in more than one Zn^2+^-mediated way.

In addition, zinc is closely linked with insulin regulation; it is important for insulin crystallisation, storage in the β-cells of the pancreas, and release into plasma.^59–61^ Zn^2+^ binding by HSA has also been shown to promote dissociation of Zn^2+^-stabilised hexameric insulin into monomers (the biologically active form of insulin) after exocytosis from β-cells.^62^ Thus, this is another pathway in which the interference of elevated NEFA levels with Zn^2+^-binding by HSA can negatively impact on T2DM.

Despite the negative effects of additional non-HSA bound zinc in plasma that we have shown, many individuals with T2DM take zinc supplementation because of their zinc deficiency, and this has been shown to have beneficial effects (improved insulin and glucose levels and decreased risk of developing T2DM).^63–65^ However, while supplementation may increase zinc availability throughout the body, it likely also increases zinc binding to other plasma proteins,^10^ including coagulation proteins, with further consequences for thrombotic risk in individuals with T2DM. Heparin, an important anticoagulant, has been shown to be increasingly neutralised in the presence of elevated Zn^2+^ levels,^15,66^ in particular by histidine-rich glycoprotein, and we have previously indicated that this is one of the proteins that can bind “excess” Zn^2+^ when NEFAs alter Zn^2+^-binding by HSA.^10,20^ Thus, the effect of high plasma levels of NEFA and altered Zn^2+^ speciation may need to be carefully considered when choosing an antithrombotic treatment for individuals with T2DM.

T2DM is not the only condition associated with high plasma NEFAs,^50^ and although correlations await to be established, it seems prudent to suggest that plasma NEFA levels need to be carefully controlled in all cardio-metabolic disorders. This could be achieved through several means, including: (1) Change in the saturated/unsaturated NEFA ratio in the diet, as the degree of saturation of the NEFAs appears to make a difference. (2) The use of statins or fenofibrate as, in addition to affecting cholesterol or triglyceride levels, such drugs have been shown to reduce plasma NEFA levels.^29,67,68^ (3) Targeting fatty acid synthase in order to reduce *de novo* synthesis of NEFAs – this could be particularly important in cancer-associated thrombosis, as expression of this enzyme is frequently increased in tumour cells.^69^ (4) Design and administration of a small molecule able to inhibit binding of NEFAs to the FA2 site on HSA, whilst not impairing Zn^2+^ binding to site A.

In conclusion, this study demonstrates that plasma NEFA levels correlate with increased clot density in T2DM. It is also revealed that the NEFA species that correlate with the maximum absorbance of fibrin clot all have elevated concentrations in T2DM and that those among them that have been studied with ITC have been shown to strongly perturb Zn^2+^-binding to HSA. Furthermore, addition of Zn^2+^ to plasma induces similar changes in clot density as is observed in T2DM. Thus, it appears likely that NEFAs influence available/labile plasma Zn^2+^ concentrations by a mechanism that involves the FA2 site on HSA, such as to ultimately exert a pro-coagulatory effect. Given that plasma NEFA levels can be controlled pharmacologically or *via* dietary intervention, we believe these findings will be useful for the treatment and management of T2DM and other disorders associated with elevated NEFA levels.

## Abbreviations

ANOVA: Analysis of variance
BSA: Bovine serum albumin
CFT: Clot formation time;
FAME: Fatty acid methyl esters
GC-MS: Gas chromatography-mass spectrometry
HDL: High density lipoprotein
HSA: Human serum albumin
ITC: Isothermal titration calorimetry
LDL: Low density lipoprotein
mol. eq.: Molar equivalents
NEFA: Non-esterified fatty acid
TPEN: *N*,*N*,*N*′,*N*′-Tetrakis(2-pyridinylmethyl)-1,2-ethanediamine;
SD: Standard deviation
SEM: Scanning electron microscopy
T2DM: Type-2 diabetes mellitus;
tPA: Tissue plasminogen activator
Tris: Tris(hydroxymethyl)aminomethane

## Author contributions

A. I. S. S., K. G. H. K., F. A. P., S. K., R. Y., and F. S. performed experiments; A. I. S. S., K. G. H. K., C. A. B., S. J. P., T. K. S., R. A. A. and A. J. S. analysed and interpreted the results; A. I. S. S., K. G. H. K., C. A. B., S. J. P., R. A. A. and A. J. S. wrote the paper; F. L., C. A. B., S. J. P., T. K. S., R. A. A. and A. J. S. designed the research.

## Conflicts of interest

There are no conflicts to declare.

## Supplementary Material

SC-012-D0SC06605B-s001
