# Supplementary material for: Albumin-mediated alteration of plasma zinc speciation by fatty acids modulates blood clotting in type-2 diabetes
Source: Chem Sci. 2021 Feb 1;12(11):4079–93. doi: 10.1039/d0sc06605b (PMC8179462; doi:10.1039/d0sc06605b)
Supplement: SC-012-D0SC06605B-s001 [file SC-012-D0SC06605B-s001.pdf]

Electronic Supplementary Material (ESI) for Chemical Science.
This journal is © The Royal Society of Chemistry 2021

Supplementary Information

Albumin-mediated alteration of plasma zinc speciation by fatty acids modulates blood clotting in type-2 diabetes

Fatty acids increase blood coagulability in type-2 diabetes through altered plasma zinc speciation

Amélie I. S. Sobczak, Kondwani G. H. Katundu, Fladia A. Phoenix, Siavash Khazaipoul, Ruitao Yu, Fanuel Lampiao, Fiona Stefanowicz, Claudia A. Blindauer, Samantha J. Pitt, Terry K. Smith, Ramzi A. Ajjan, and Alan J. Stewart

Table S1. (Step-wise) stability constants used in speciation modelling.

Table S2. Concentrations employed for speciation modelling.

Table S3. Fitting parameters used for the fitting of the ITC experiments.

Table S4. Fitting results from replicate ITC experiments carried out in the absence of NEFAS.

Table S5. Fitting results from ITC experiments.

Figure S1. ITC raw data. Full ITC data (including raw data) for the first replicate of Zn^{2+} binding to 60 μM HSA in the absence of NEFA.

Figure S2. ITC raw data. Full ITC data (including raw data) for the second replicate of Zn^{2+} binding to 60 μM HSA in the absence of NEFA.

Figure S3. ITC raw data. Full ITC data (including raw data) for the third replicate of Zn^{2+} binding to 60 μM HSA in the absence of NEFA.

Figure S4. ITC raw data. Full ITC data (including raw data) for the fourth replicate of Zn^{2+} binding to 60 μM HSA in the absence of NEFA.

Figure S5. ITC raw data. Full ITC data (including raw data) for Zn^{2+} binding to 60 μM HSA in the presence of 3 mol. eq. of octanoate.

Figure S6. ITC raw data. Full ITC data (including raw data) for Zn^{2+} binding to 60 μM HSA in the presence of 4 mol. eq. of octanoate.

Figure S7. ITC raw data. Full ITC data (including raw data) for Zn^{2+} binding to 60 μM HSA in the presence of 5 mol. eq. of octanoate.

Figure S8. ITC raw data. Full ITC data (including raw data) for Zn^{2+} binding to 60 μM HSA in the presence of 3 mol. eq. of laurate.

Figure S9. ITC raw data. Full ITC data (including raw data) for Zn^{2+} binding to 60 μM HSA in the presence of 4 mol. eq. of laurate.

Figure S10. ITC raw data. Full ITC data (including raw data) for Zn^{2+} binding to 60 μM HSA in the presence of 5 mol. eq. of laurate.

Figure S11. ITC raw data. Full ITC data (including raw data) for Zn^{2+} binding to 60 μM HSA in the presence of 3 mol. eq. of myristate.

Figure S12. ITC raw data. Full ITC data (including raw data) for Zn^{2+} binding to 60 µM HSA in the presence of 4 mol. eq. of myristate.

Figure S13. ITC raw data. Full ITC data (including raw data) for Zn^{2+} binding to 60 µM HSA in the presence of 5 mol. eq. of myristate.

Figure S14. ITC raw data. Full ITC data (including raw data) for Zn^{2+} binding to 60 µM HSA in the presence of 3 mol. eq. of palmitate.

Figure S15. ITC raw data. Full ITC data (including raw data) for Zn^{2+} binding to 60 µM HSA in the presence of 4 mol. eq. of palmitate.

Figure S16. ITC raw data. Full ITC data (including raw data) for Zn^{2+} binding to 60 µM HSA in the presence of 5 mol. eq. of palmitate.

Figure S17. ITC raw data. Full ITC data (including raw data) for Zn^{2+} binding to 25 µM HSA in the absence of NEFA.

Figure S18. ITC raw data. Full ITC data (including raw data) for Zn^{2+} binding to 25 µM HSA in the presence of 2.5 mol. eq. of palmitate.

Figure S19. ITC raw data. Full ITC data (including raw data) for Zn^{2+} binding to 25 µM HSA in the presence of 4 mol. eq. of palmitate.

Figure S20. ITC raw data. Full ITC data (including raw data) for Zn^{2+} binding to 25 µM HSA in the presence of 5 mol. eq. of palmitate.

Figure S21. ITC raw data. Full ITC data (including raw data) for Zn^{2+} binding to 25 µM HSA in the presence of 2.5 mol. eq. of stearate.

Figure S22. ITC raw data. Full ITC data (including raw data) for Zn^{2+} binding to 25 µM HSA in the presence of 4 mol. eq. of stearate.

Figure S23. ITC raw data. Full ITC data (including raw data) for Zn^{2+} binding to 25 µM HSA in the presence of 5 mol. eq. of stearate.

Figure S24. ITC raw data. Full ITC data (including raw data) for Zn^{2+} binding to 25 µM HSA in the presence of 2.5 mol. eq. of palmitoleate.

Figure S25. ITC raw data. Full ITC data (including raw data) for Zn^{2+} binding to 25 µM HSA in the presence of 4 mol. eq. of palmitoleate.

Figure S26. ITC raw data. Full ITC data (including raw data) for Zn^{2+} binding to 25 µM HSA in the presence of 5 mol. eq. of palmitoleate.

Figure S27. Representative raw data from turbidimetric fibrin clotting and lysis assays.

Figure S28. Effects of Zn^{2+} and NEFAs on fibrin clot parameters in pooled plasma and effects relative to the parameter values in the absence of Zn^{2+}.

Figure S29. Effects of Zn^{2+} and NEFAs on fibrin clot parameters in a purified system and effects relative to the parameter values in the absence of Zn^{2+}.

Table S6. Demographic information and the results from plasma analysis for the T2DM and control subjects.

Figure S30. Comparisons of NEFA, zinc, HSA and fibrinogen concentrations and of platelet count between sexes in plasma samples from individuals with T2DM and controls.

Figure S31. Comparison of the clot formation and lysis parameters in plasma samples from patient with T2DM and controls.

Figure S32. Comparison of clotting parameters between sexes in plasma samples from patients with T2DM and controls.

Figure S33. Representative image from SEM experiments. Purified system, no zinc.

Figure S34. Representative image from SEM experiments. Purified system, 18 μM zinc.

Figure S35. Representative image from SEM experiments. Controls, no zinc.

Figure S36. Representative image from SEM experiments. Controls, 50 μM zinc.

Figure S37. Representative image from SEM experiments. T2DM, no zinc.

Figure S38. Representative image from SEM experiments. T2DM, 50 μM zinc.

Figure S39. Minimum increase in available Zn^{2+} concentration upon addition of 4 mol. eq. of myristate to 600 μM HSA, in dependence on total Zn^{2+} concentration.

Table S1. (Step-wise) stability constants used in speciation modelling.

Species	log K/p*K*a	Reference
Zn(HSA site A)	5.89	This work
Zn(HSA site B)	4.16	
H(Cit)	5.51	S.R. Hurford, C.R. Moris, J.A. Vesey, D, R Williams, D. Cummins, P. I. Riley, G. L. Christie and J. R. Duffield, *J. Inorg. Biochem.*, 1991, **42**, 273-287.
H_2(Cit)	4.23	
Zn(Cit)	4.55	
$Zn(Cit)_2$	2.32	
Ca(Cit)	3.364	C. Blaquiere and G. Berthon, *Inorg. Chim. Acta*, 1987, **135**,179-189.
$Ca(Cit)_2$	1.601	
Zn(Tris)	2.271	L. Bologni, A. Sabatini and A. Vacca, *Inorg. Chim. Acta*, 1983, **69**, 71-75.
H(Tris)	8.11	

Table S2. Concentrations employed for speciation modelling. In μM unless stated otherwise.

System	Concentrations					
	Zn^{2+}	HSA site A	HSA site B	Citrate	Tris	Ca^{2+}
Platelets in plasma	15 or 115	600, 540 or 38 [a)]	600	n.a.	n.a.	n.a.
Citrated plasma (fibrin clotting)	2.5, 21.83, 41.17 or 99.17	100 or 16.3	100	2.07 or 2.45 mM	41.67 mM	7.5 mM
Purified system (fibrin clotting)	0, 20, 40, 100	100 or 16.3	100	n.a.	50 mM	n.a.
Purified system (Fibre thickness)	18	135	135	n.a.	50 mM	n.a.

[a)] The 540 and 38 μM concentrations are based on the assumption of presence of endogenous NEFAs that reduce site A availability by 10%.

Table S3. Fitting parameters used for the fitting of the ITC experiments. ITC data fitting approaches for ITC experiments examining Zn^{2+}-binding to HSA in the presence of different NEFAs. The values for K1' and ΔH1 in the fits for experiments conducted in the presence of NEFAs are derived from the fit "no NEFA" (see Table S4). All entries marked "v" signify parameters that were allowed to vary. Results for these varied parameters are given in Table S5.

Fit	Model	Fixed parameters					
		N1	*K*1' (M^{-1})	ΔH1 (kcal mol^{-1})	N2	*K*2' (M^{-1})	ΔH2 (kcal mol^{-1})
No NEFA, 60 µM HSA	Two sets of sites	v	v	v	v	v	v
Others	Two sets of sites	v	307313	-6490	1.00	v	v

Table S4. Fitting results from replicate ITC experiments carried out in the absence of NEFAS.

	N1	*K*1' (M^{-1})	ΔH1 (kcal mol^{-1})	N2	*K*2' (M^{-1})	ΔH2 (kcal mol^{-1})
Repeat 1	0.918	369280	-6407	1.00	9028	-11246
Repeat 2	0.961	84639	-7347	1.00	7244	-10576
Repeat 3	0.862	468020	-5717	1.00	6001	-12566
Mean	0.914	307313	-6490	1.00	7424	-11463
SD	0.040	162532	668	0	1242	827

Table S5. Fitting results from ITC experiments.

Fitted Parameter	Fit	0 NEFA	2.5 NEFA	3 NEFA	4 NEFA	5 NEFA
N1	No NEFA, 60 μM HSA	0.914				
K1' (M^{-1})	(average)	307313				
ΔH1 (kcal mol^{-1})		-6490				
N2		1.00				
K2' (M^{-1})		7424				
ΔH2 (kcal mol^{-1})		-11463				
N1	Octanoate			0.816	0.783	0.843
	Laurate			0.836	0.191	2e-14
	Myristate			0.468	0.163	0.000
	Palmitate, 60 μM HSA			0.438	0.000	1e-14
	No NEFA, 25 μM HSA	0.833				
	Palmitate, 25 μM HSA		0.764		0.054	8e-15
	Stearate		0.401		0.075	1e-19
	Palmitoleate		0.976		0.371	2e-14
K2' (M^{-1})	Octanoate			14390	11107	12933
	Laurate			9096	10750	7817
	Myristate			11864	7490	7284
	Palmitate, 60 μM HSA			12263	7557	5834
	No NEFA, 25 μM HSA	13839				
	Palmitate, 25 μM HSA		12540		7955	9658
	Stearate		25180		12386	13856
	Palmitoleate		7911		17700	8180
ΔH2 (kcal mol^{-1})	Octanoate			-6975	-4030	-5474
	Laurate			-11750	-15407	-16053
	Myristate			-9454	-14122	-10704
	Palmitate, 60 μM HSA			-11538	-12743	-12831
	No NEFA, 25 μM HSA	-12908				
	Palmitate, 25 μM HSA		-10551		-20352	-19791
	Stearate		-12926		-20565	-15890
	Palmitoleate		-20315		-14384	-15458

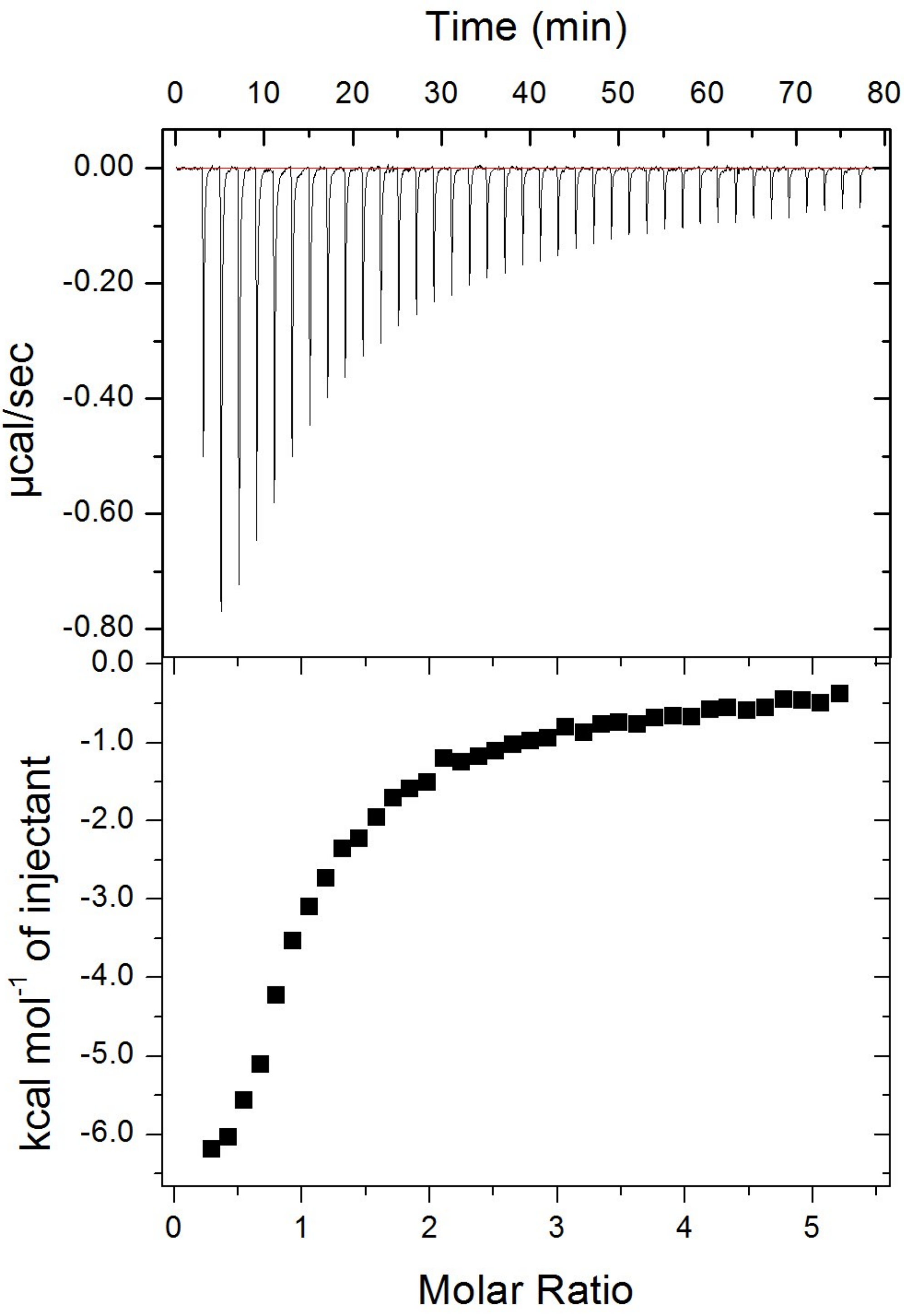

Figure S1. ITC raw data. Full ITC data (including raw data) for the first replicate of Zn^{2+} binding to 60 µM HSA in the absence of NEFA, corresponding to data shown in Figure 2A-D.

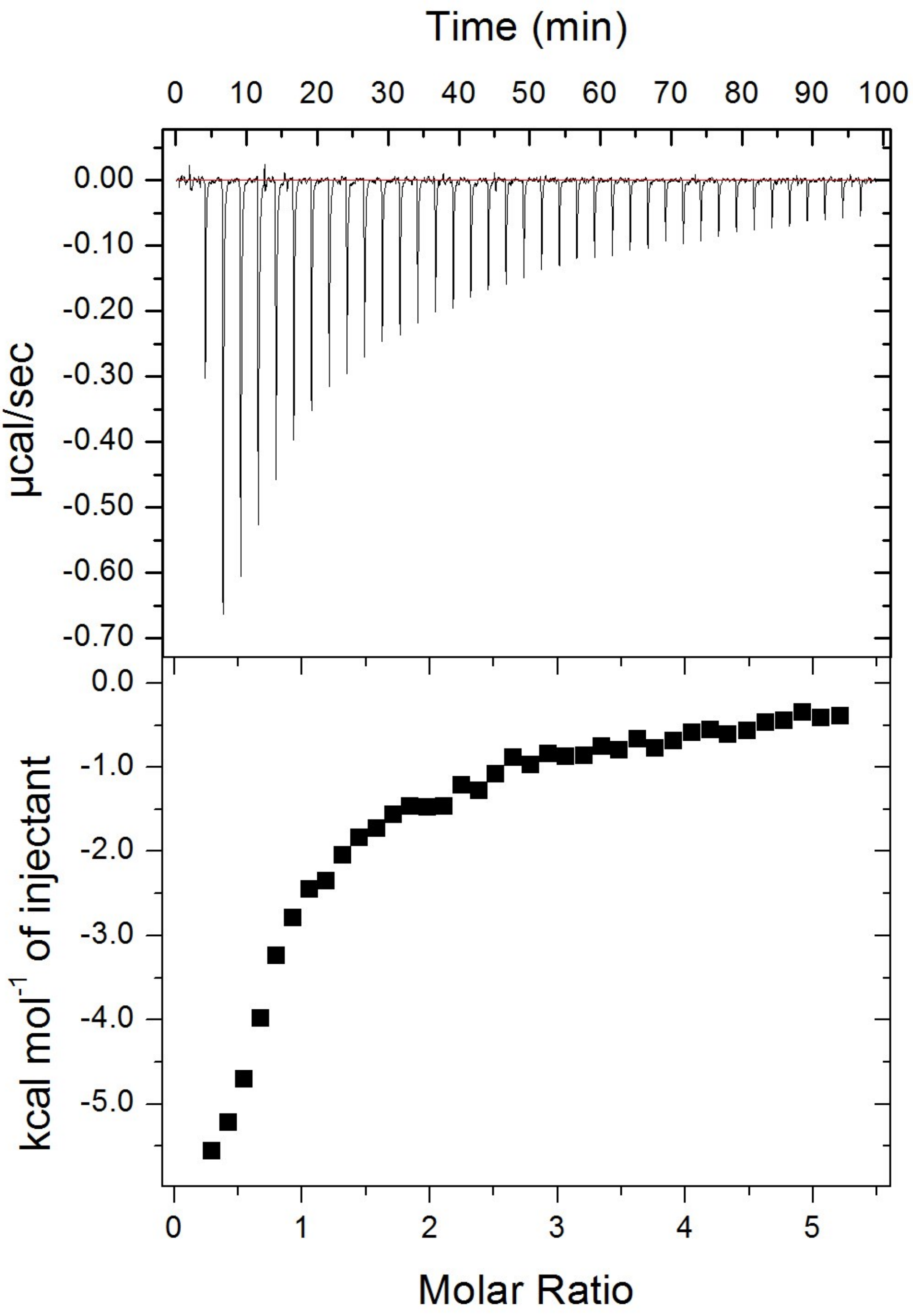

Figure S2. ITC raw data. Full ITC data (including raw data) for the second replicate of Zn^{2+} binding to 60 µM HSA in the absence of NEFA.

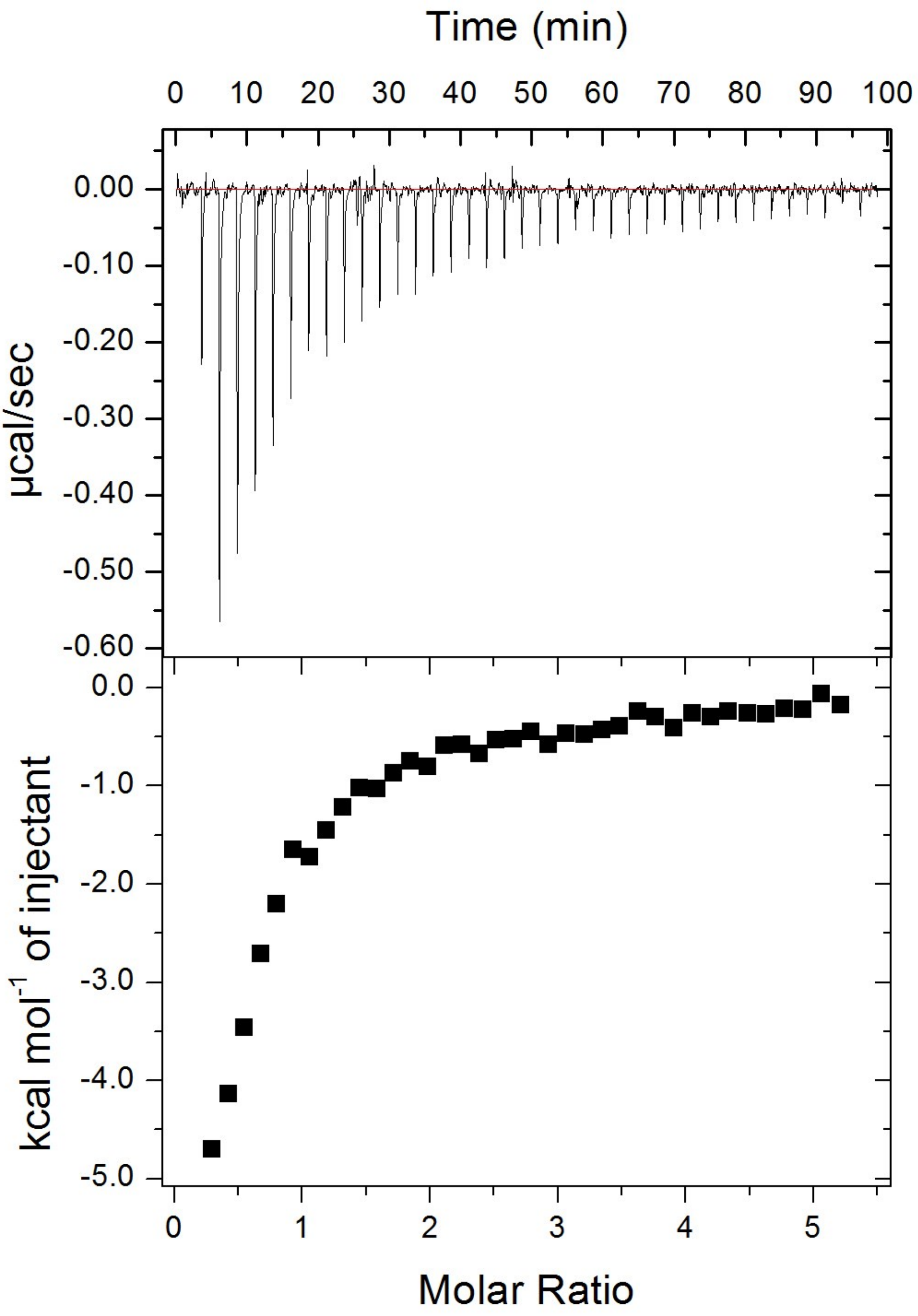

Figure S3. ITC raw data. Full ITC data (including raw data) for the third replicate of Zn^{2+} binding to 60 µM HSA in the absence of NEFA.

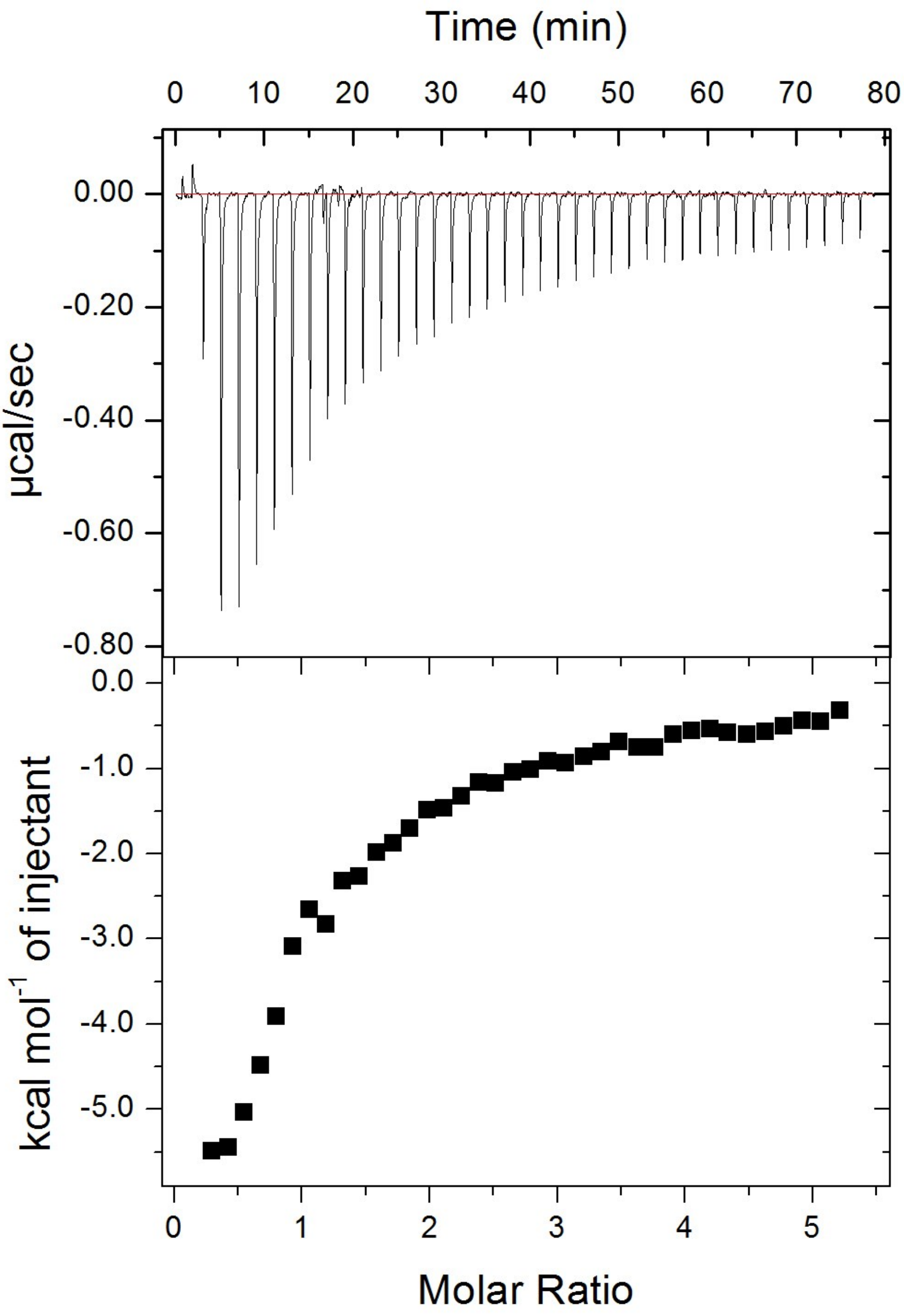

Figure S4. ITC raw data. Full ITC data (including raw data) for the fourth replicate of Zn^{2+} binding to 60 μM HSA in the absence of NEFA.

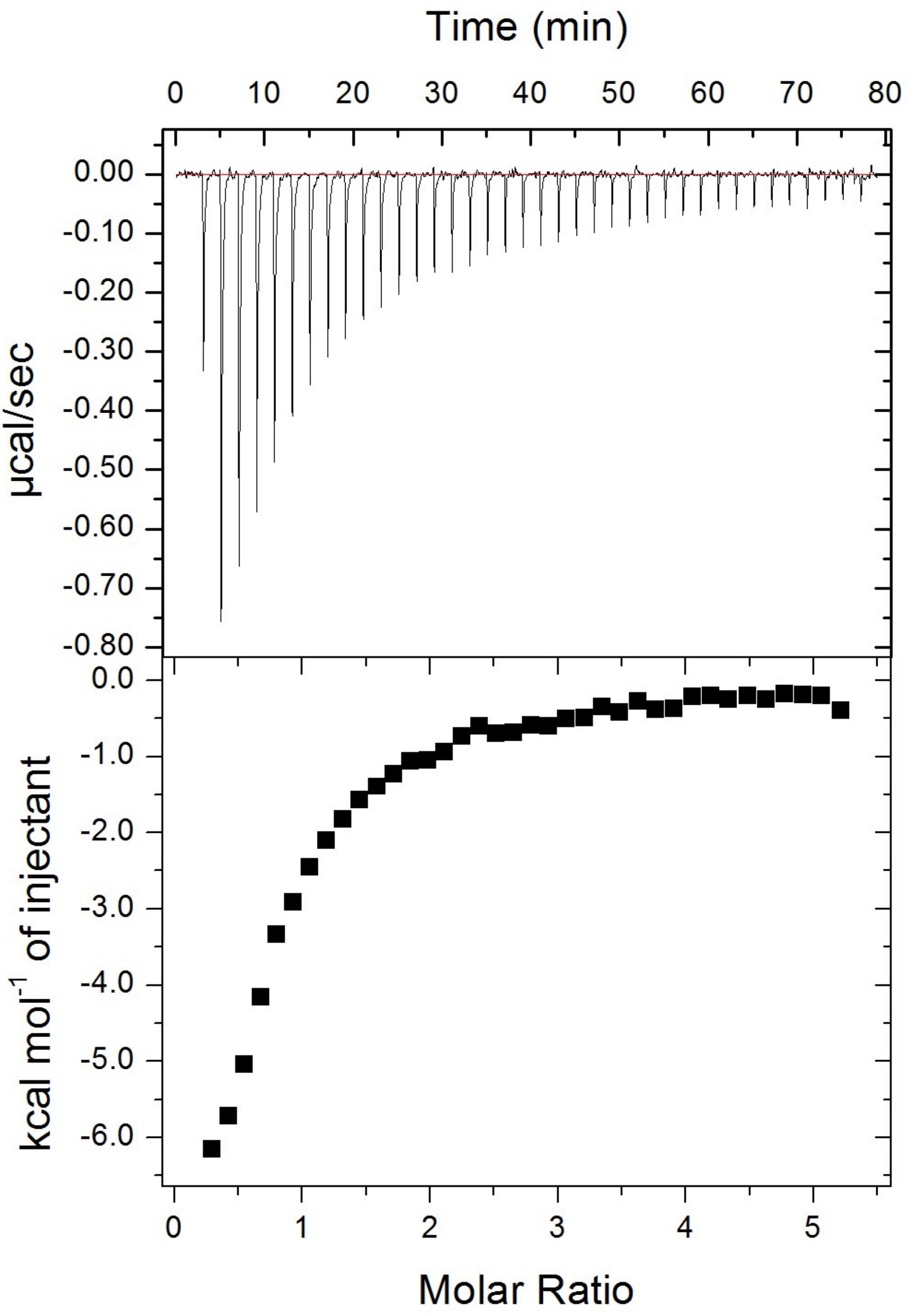

Figure S5. ITC raw data. Full ITC data (including raw data) for Zn^{2+} binding to 60 µM HSA in the presence of 3 mol. eq. of octanoate, corresponding to data shown in Figure 2A.

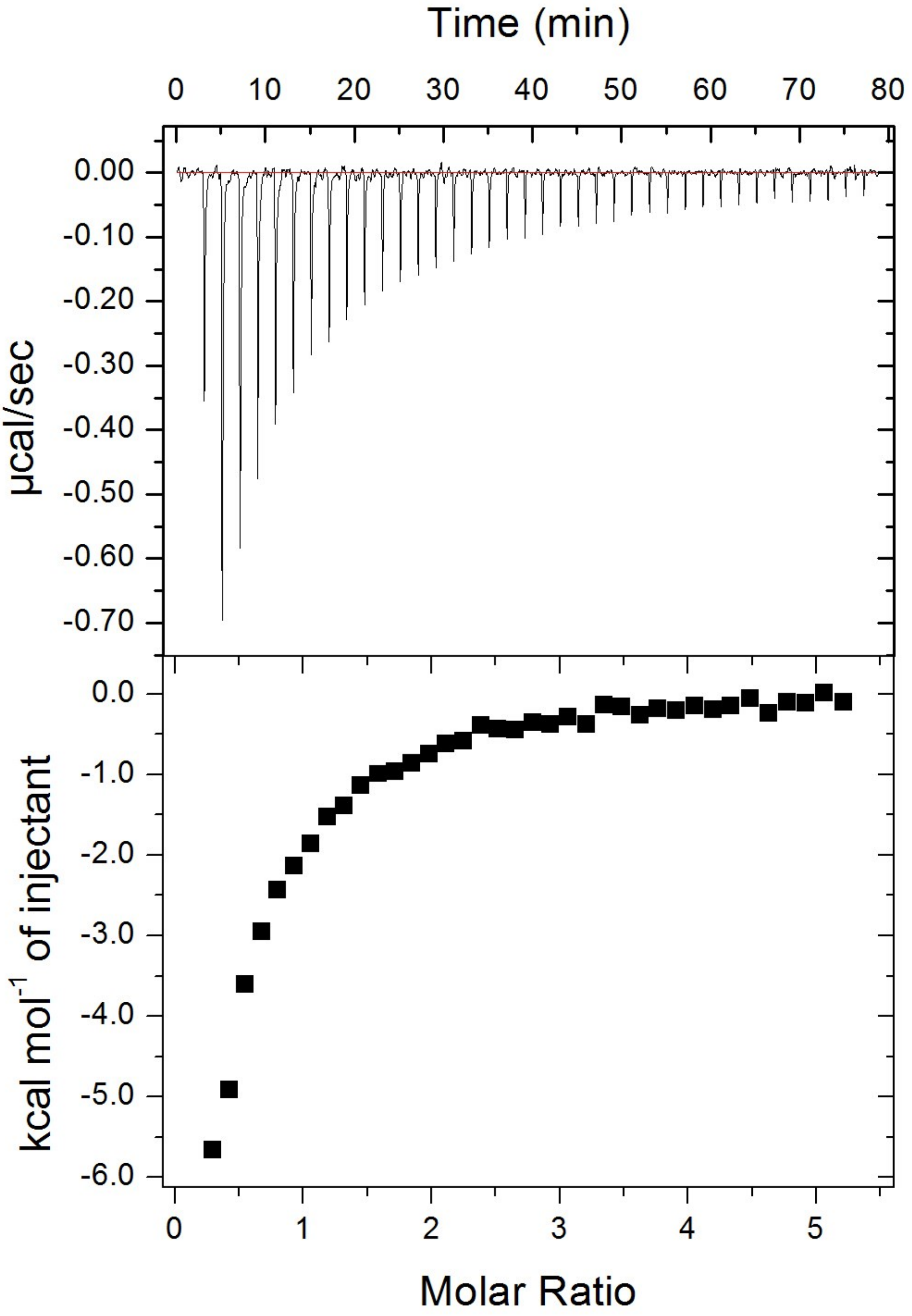

Figure S6. ITC raw data. Full ITC data (including raw data) for Zn^{2+} binding to 60 µM HSA in the presence of 4 mol. eq. of octanoate.

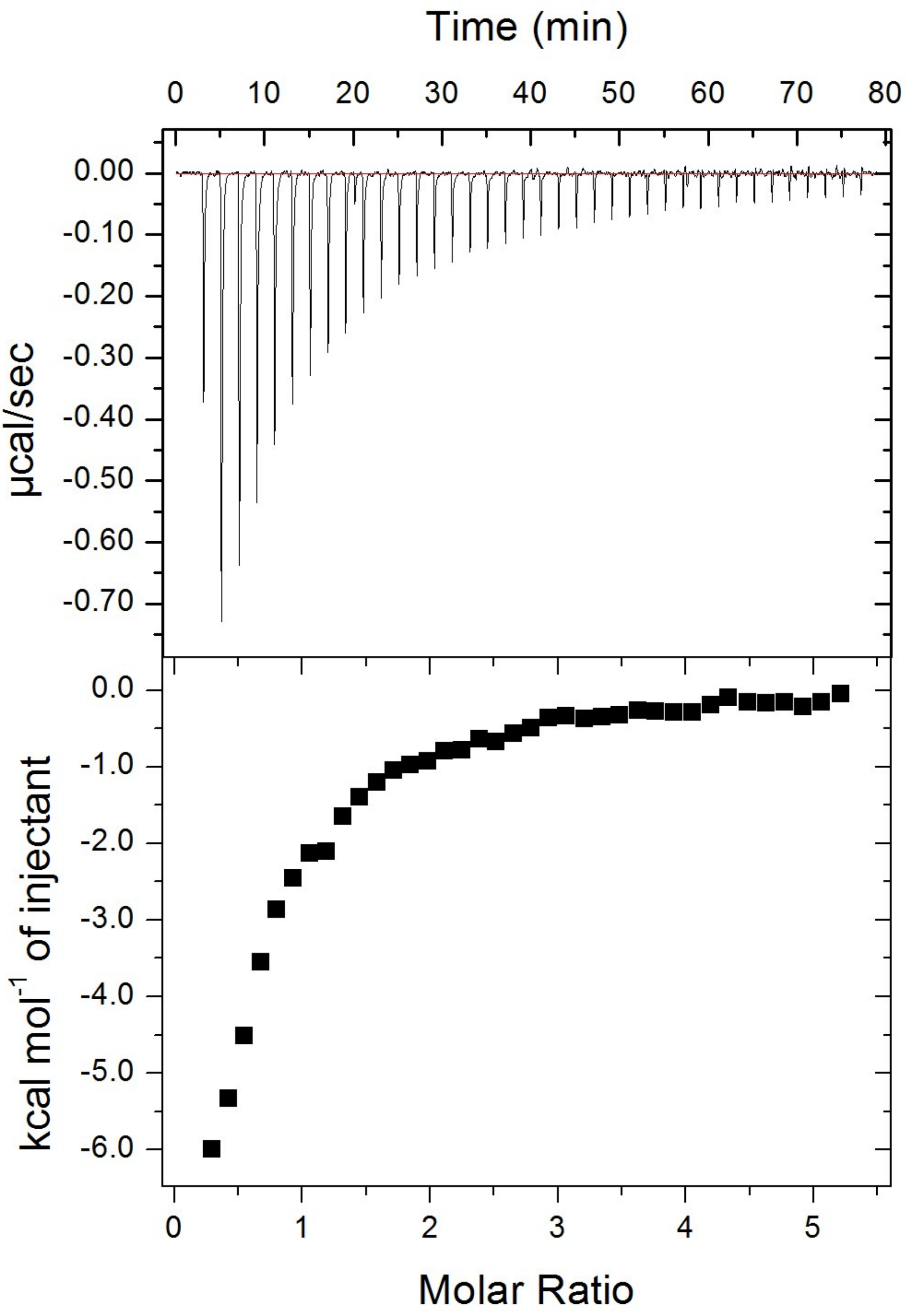

Figure S7. ITC raw data. Full ITC data (including raw data) for Zn^{2+} binding to 60 µM HSA in the presence of 5 mol. eq. of octanoate, corresponding to data shown in Figure 2A.

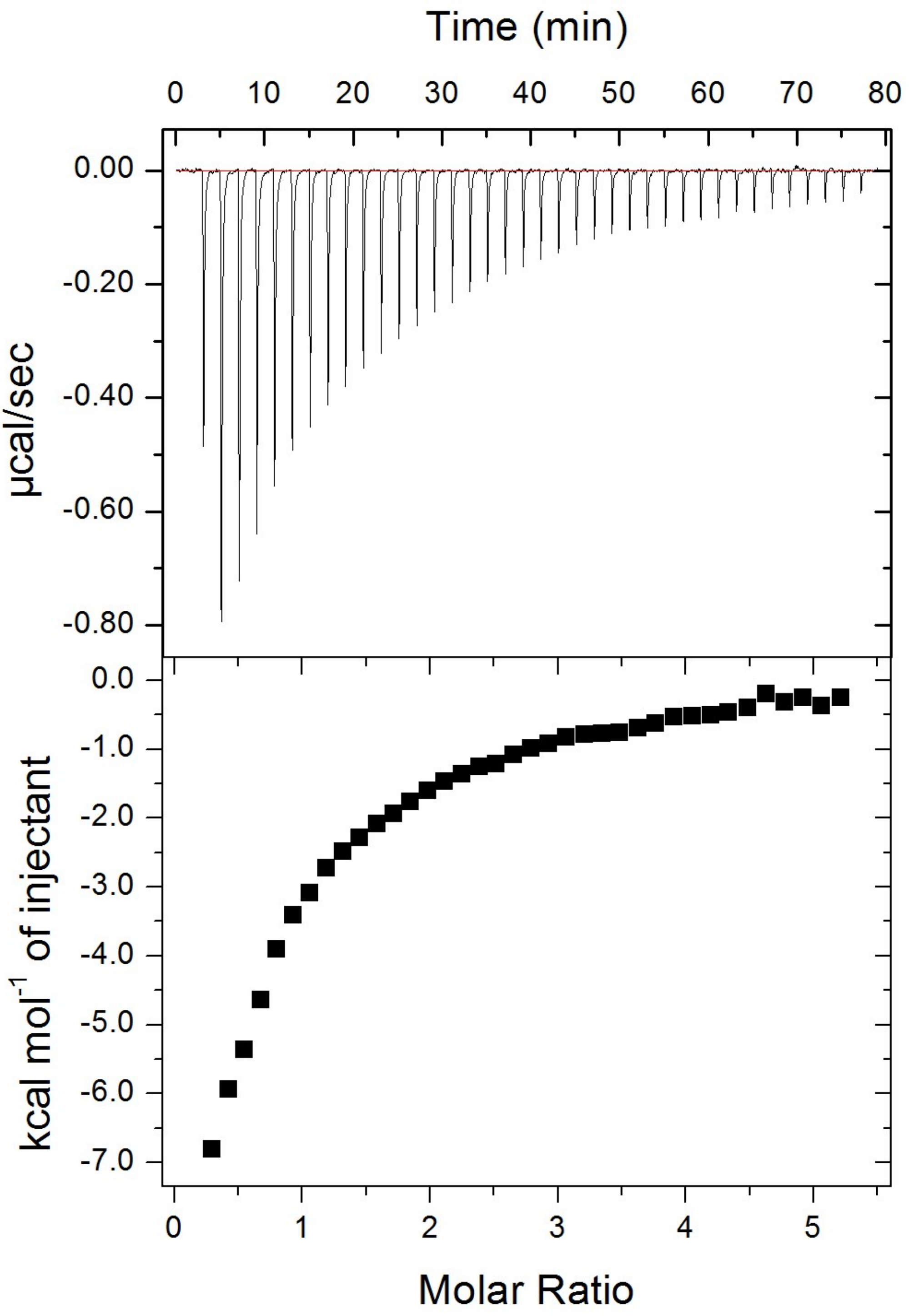

Figure S8. ITC raw data. Full ITC data (including raw data) for Zn^{2+} binding to 60 µM HSA in the presence of 3 mol. eq. of laurate, corresponding to data shown in Figure 2B.

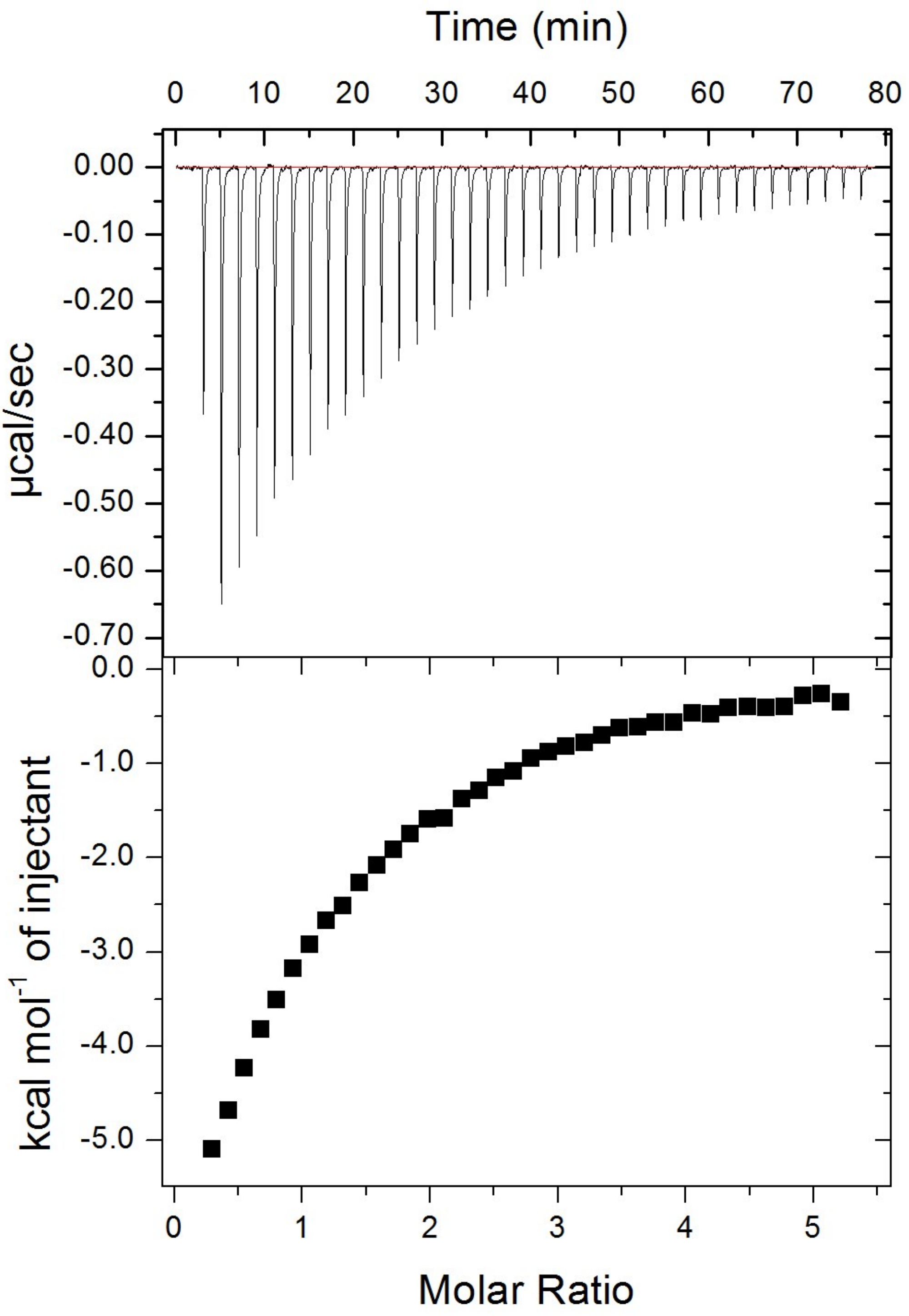

Figure S9. ITC raw data. Full ITC data (including raw data) for Zn^{2+} binding to 60 µM HSA in the presence of 4 mol. eq. of laurate, corresponding to data shown in Figure 2B.

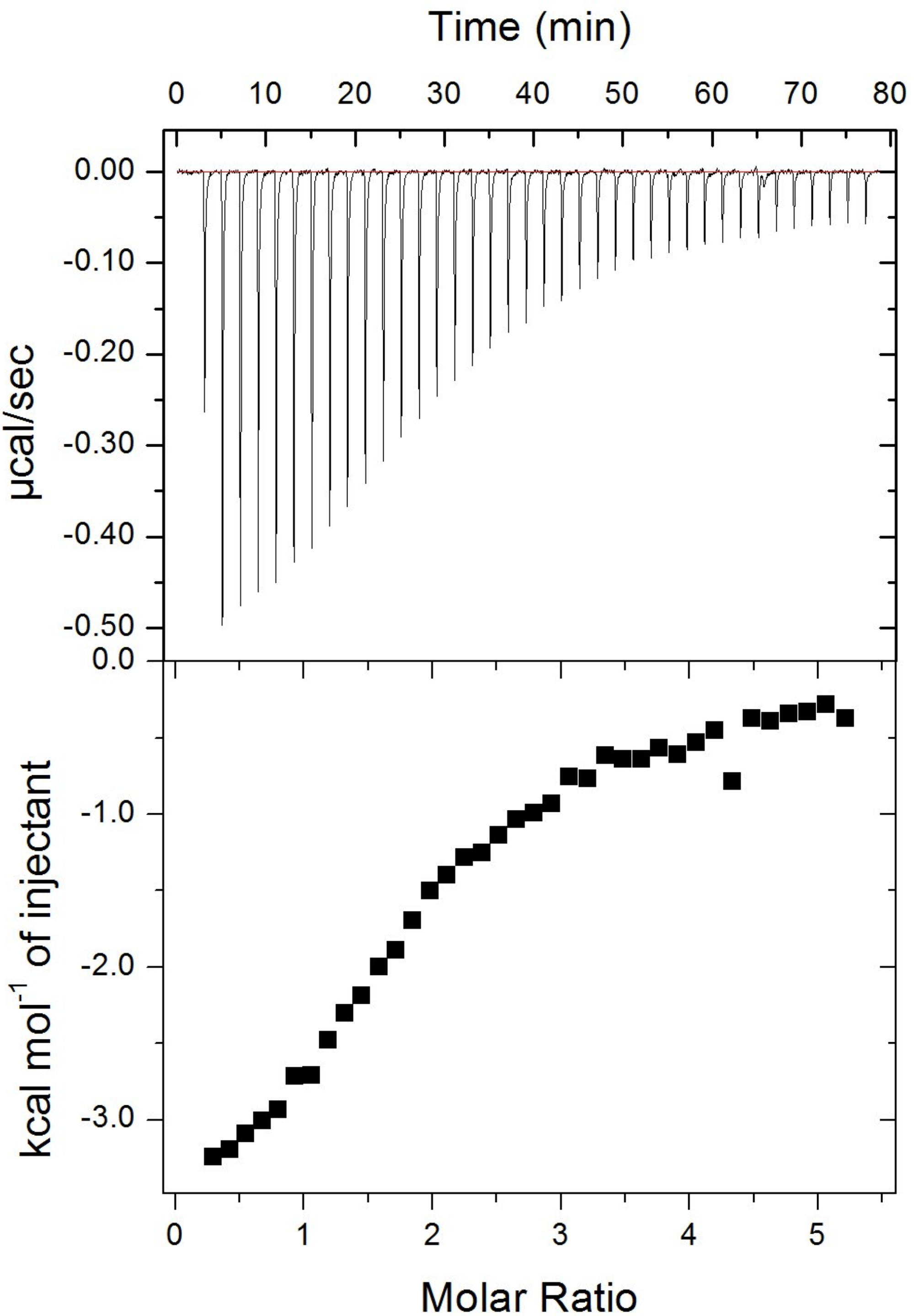

Figure S10. ITC raw data. Full ITC data (including raw data) for Zn^{2+} binding to 60 µM HSA in the presence of 5 mol. eq. of laurate, corresponding to data shown in Figure 2B.

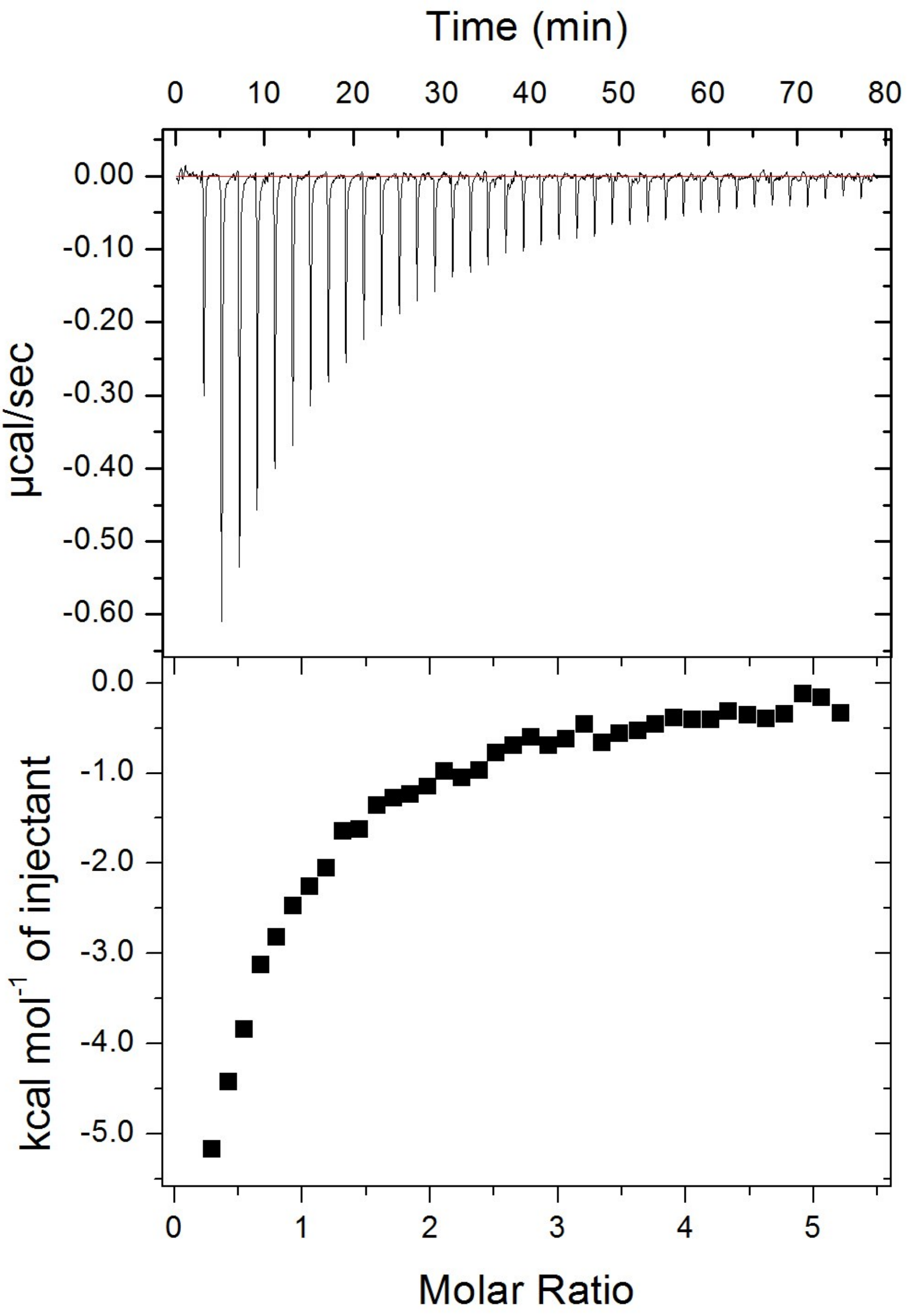

Figure S11. ITC raw data. Full ITC data (including raw data) for Zn^{2+} binding to 60 µM HSA in the presence of 3 mol. eq. of myristate, corresponding to data shown in Figure 2C.

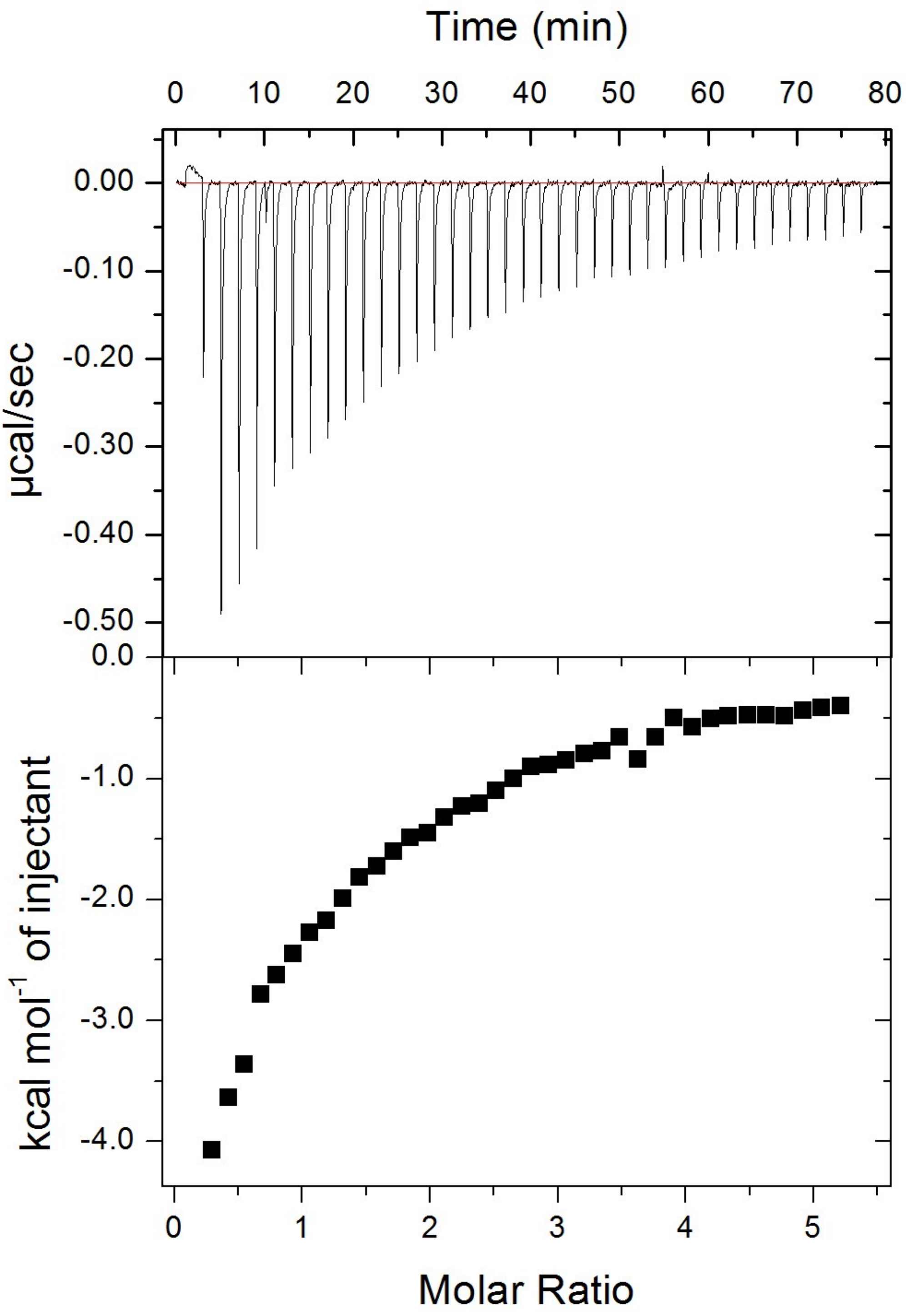

Figure S12. ITC raw data. Full ITC data (including raw data) for Zn^{2+} binding to 60 µM HSA in the presence of 4 mol. eq. of myristate, corresponding to data shown in Figure 2C.

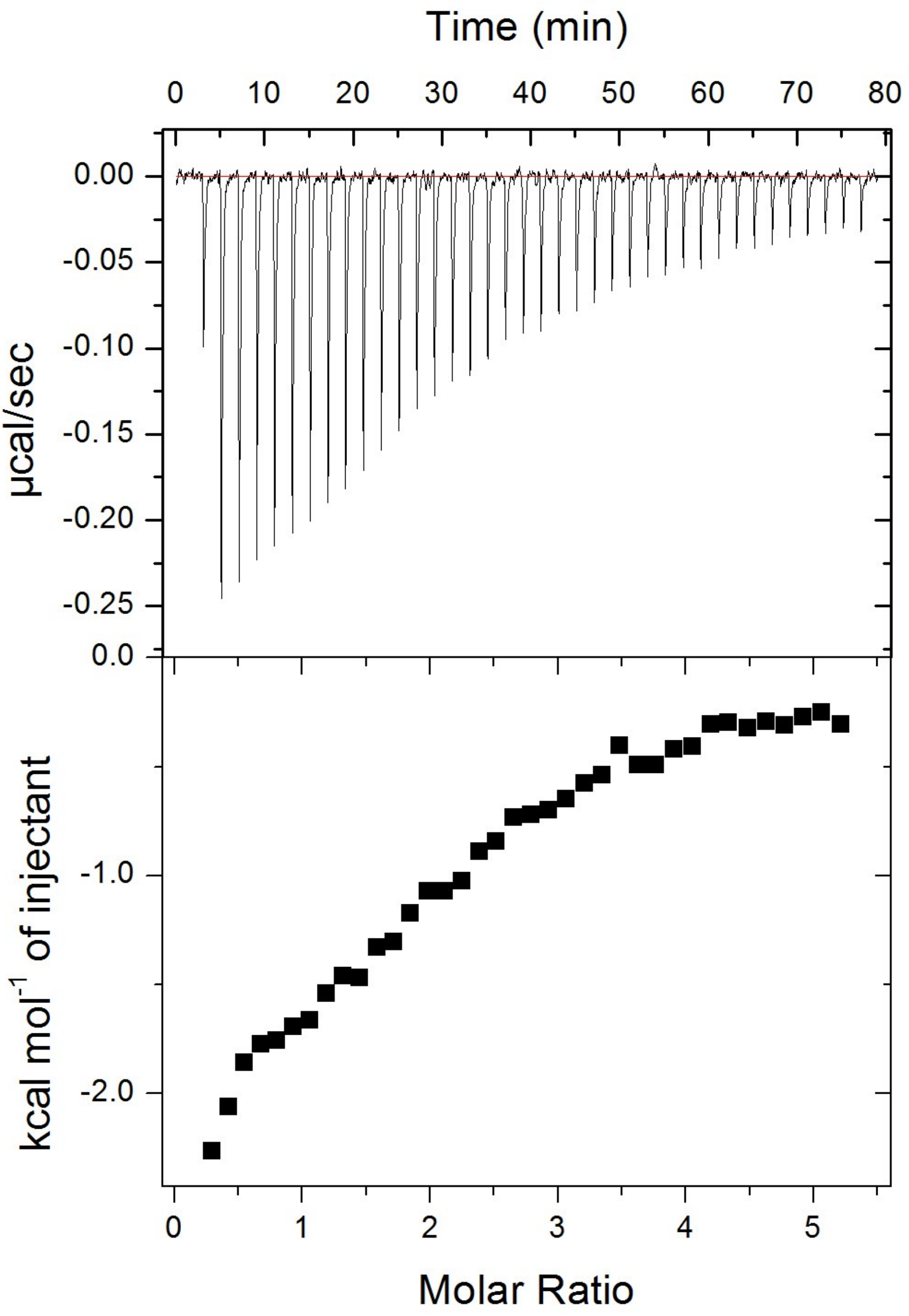

Figure S13. ITC raw data. Full ITC data (including raw data) for Zn^{2+} binding to 60 µM HSA in the presence of 5 mol. eq. of myristate, corresponding to data shown in Figure 2C.

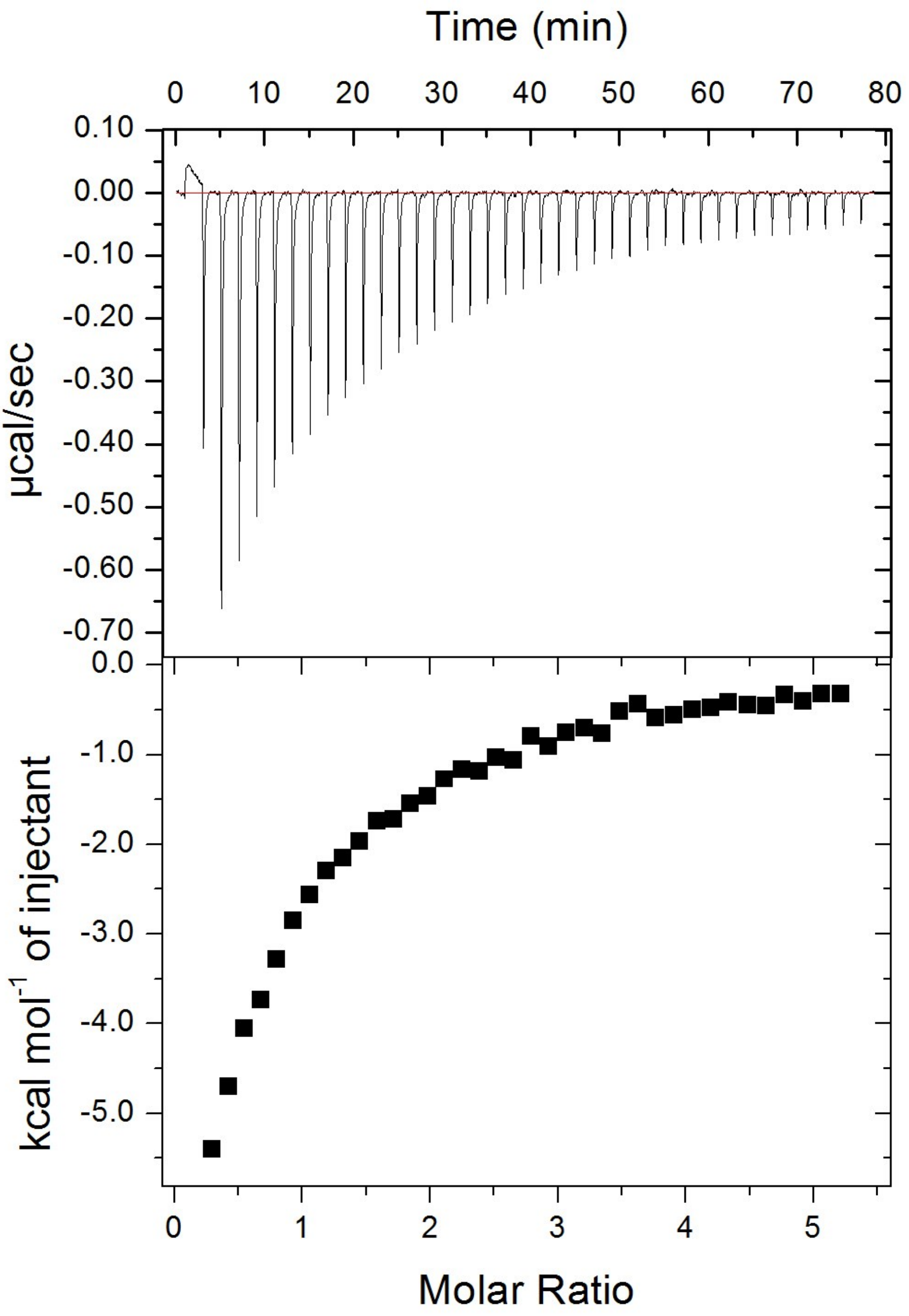

Figure S14. ITC raw data. Full ITC data (including raw data) for Zn^{2+} binding to 60 μM HSA in the presence of 3 mol. eq. of palmitate, corresponding to data shown in Figure 2D.

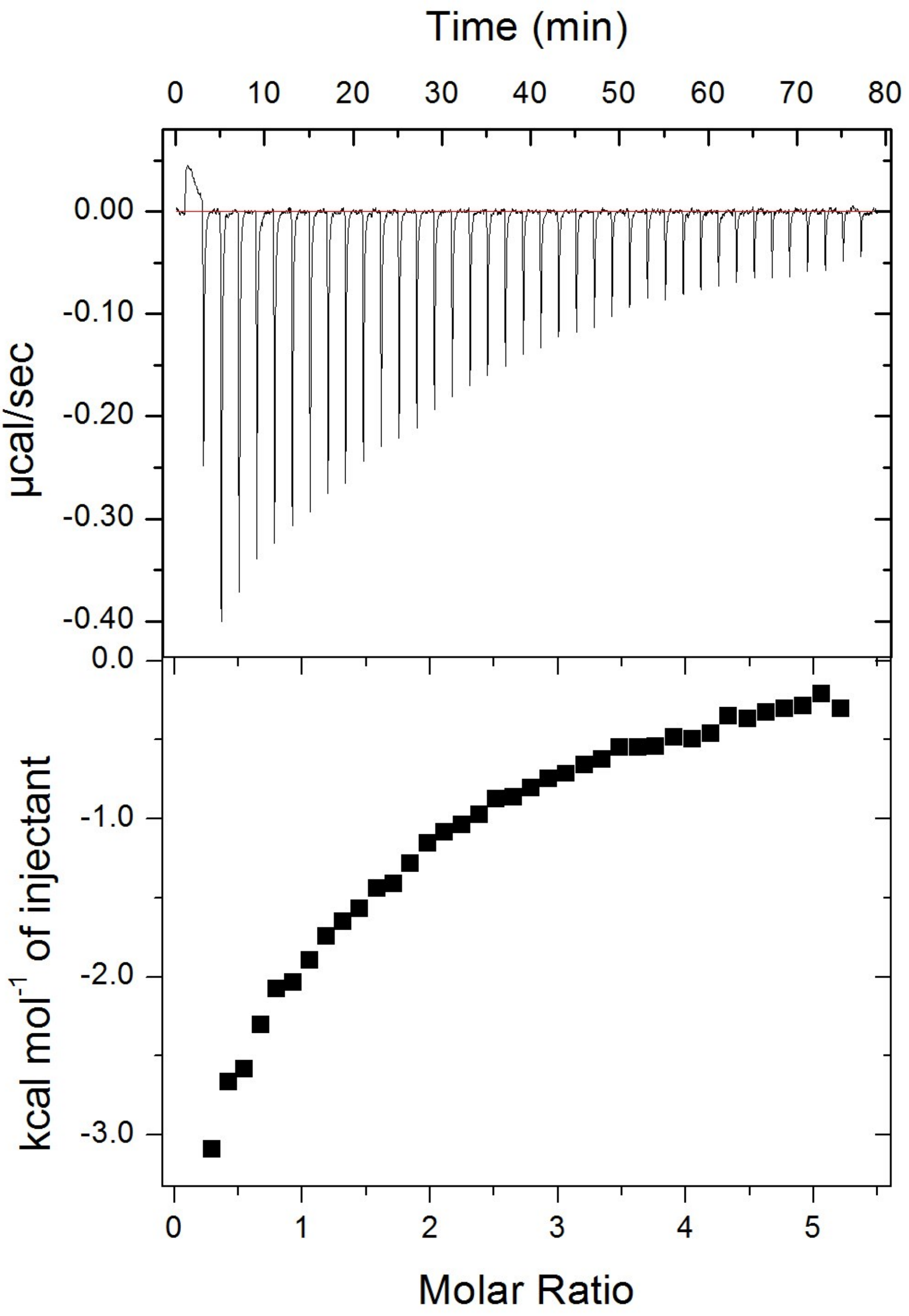

Figure S15. ITC raw data. Full ITC data (including raw data) for Zn^{2+} binding to 60 μM HSA in the presence of 4 mol. eq. of palmitate, corresponding to data shown in Figure 2D.

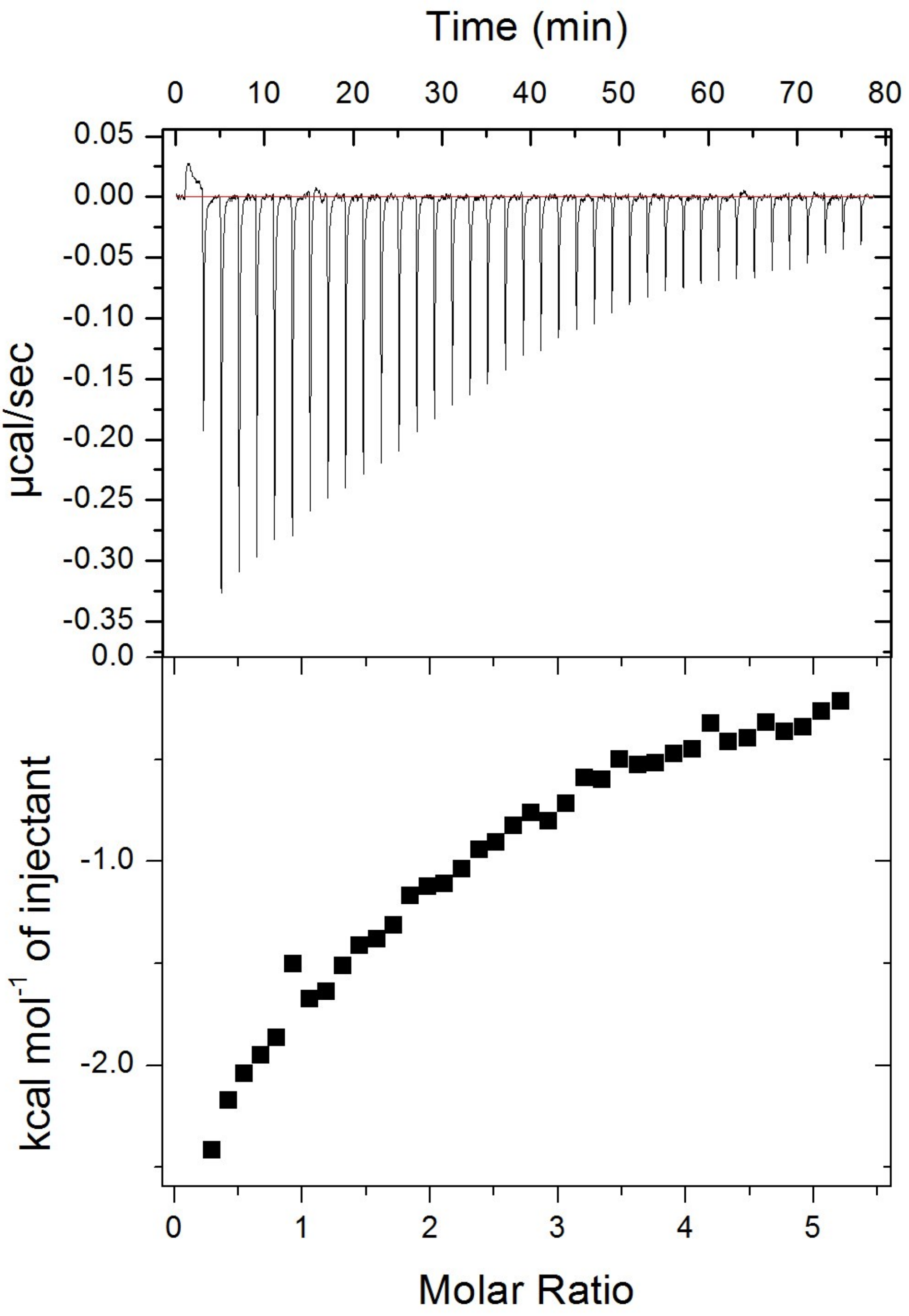

Figure S16. ITC raw data. Full ITC data (including raw data) for Zn^{2+} binding to 60 µM HSA in the presence of 5 mol. eq. of palmitate, corresponding to data shown in Figure 2D.

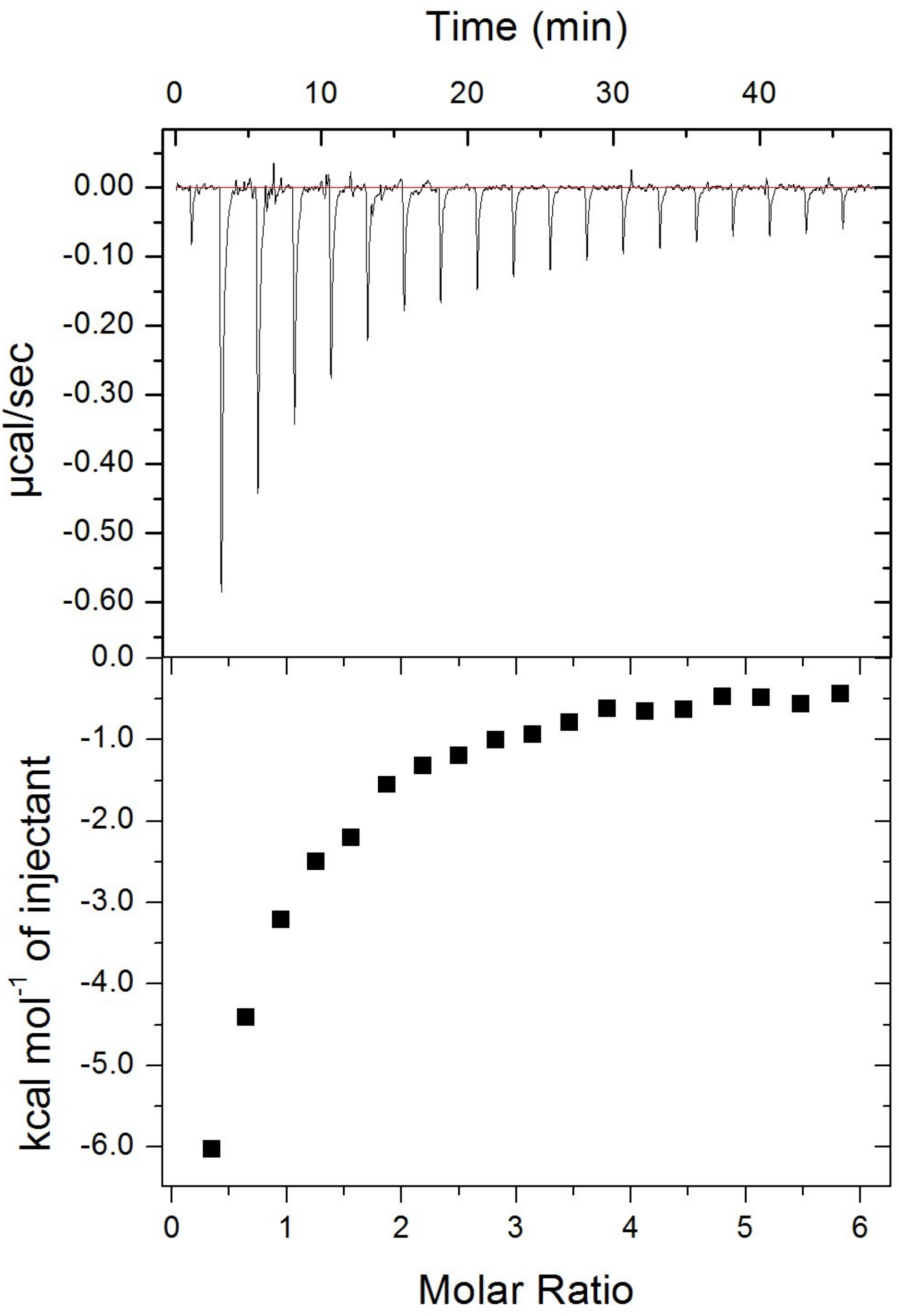

Figure S17. ITC raw data. Full ITC data (including raw data) for Zn^{2+} binding to 25 µM HSA in the absence of NEFA, corresponding to data shown in Figures 2E-H.

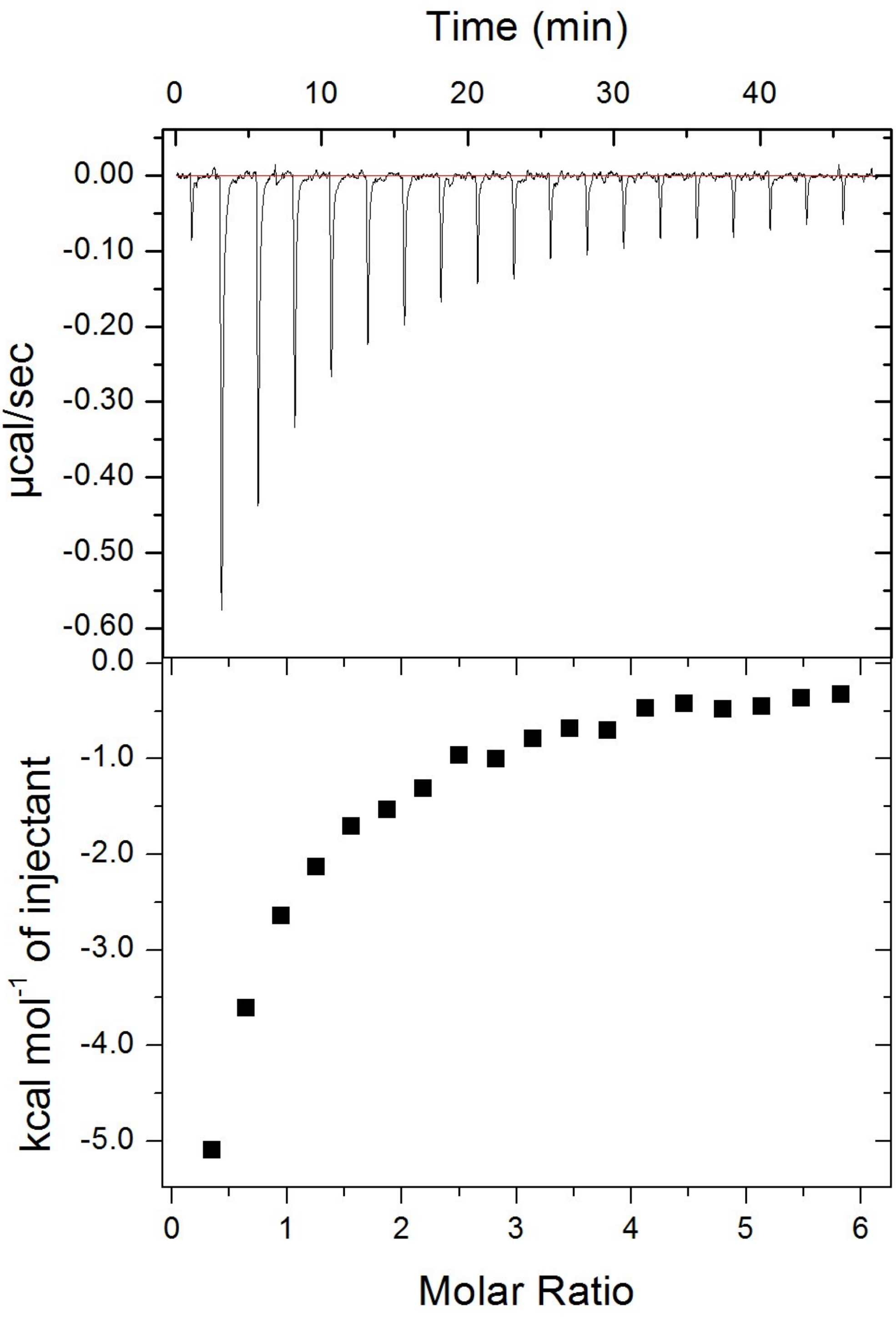

Figure S18. ITC raw data. Full ITC data (including raw data) for Zn^{2+} binding to 25 µM HSA in the presence of 2.5 mol. eq. of palmitate, corresponding to data shown in Figure 2E.

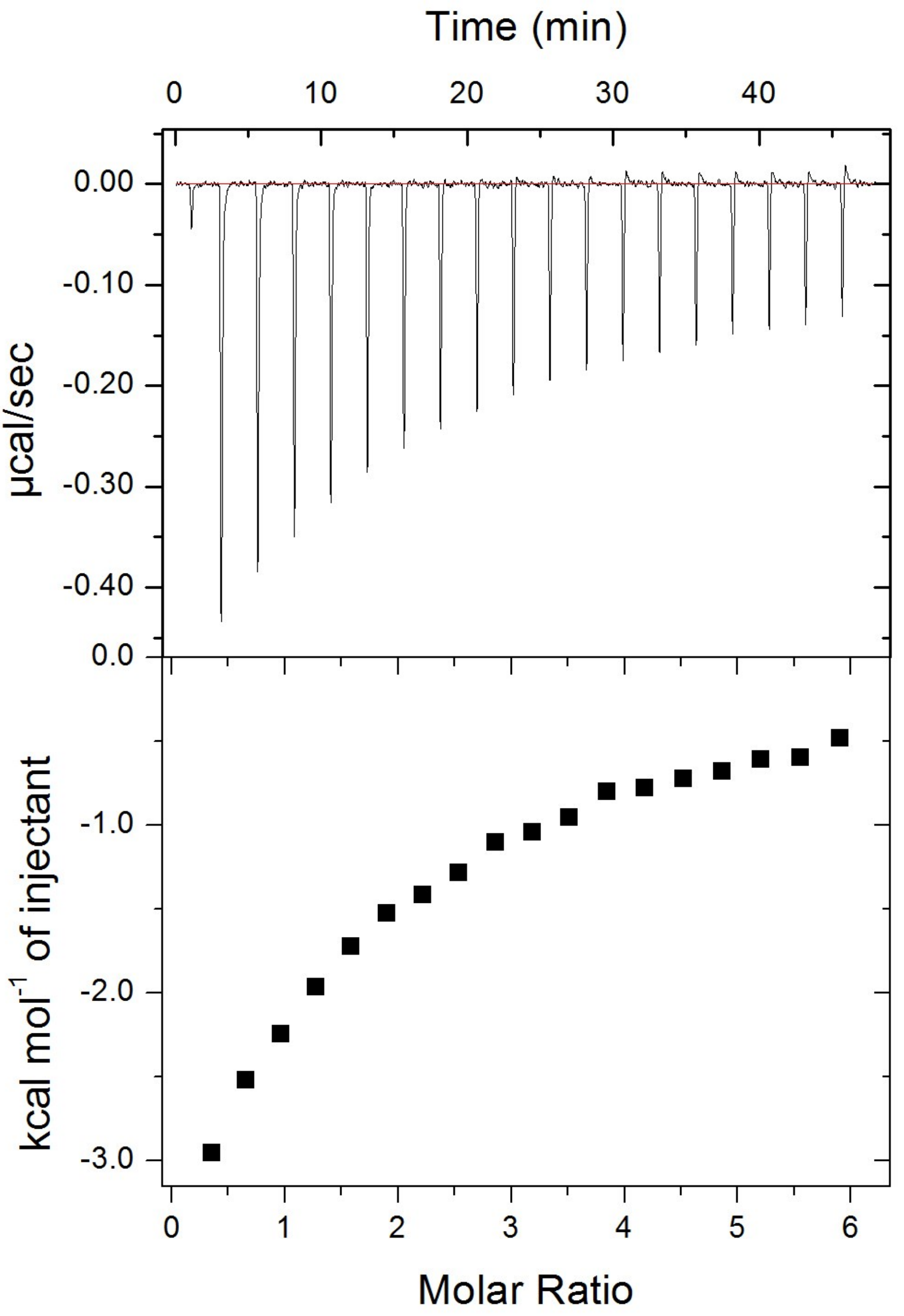

Figure S19. ITC raw data. Full ITC data (including raw data) for Zn^{2+} binding to 25 µM HSA in the presence of 4 mol. eq. of palmitate, corresponding to data shown in Figure 2E.

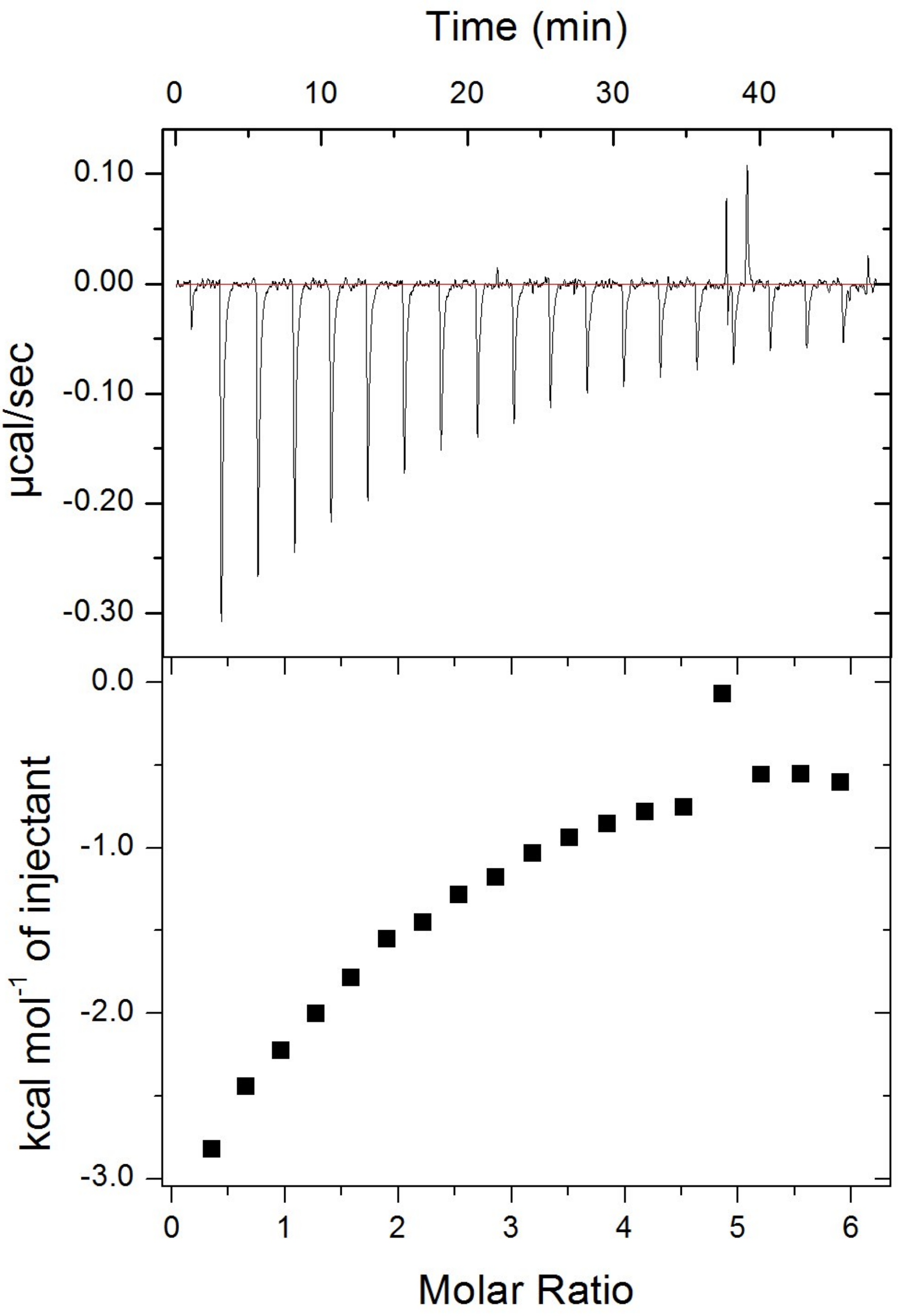

Figure S20. ITC raw data. Full ITC data (including raw data) for Zn^{2+} binding to 25 µM HSA in the presence of 5 mol. eq. of palmitate, corresponding to data shown in Figure 2E.

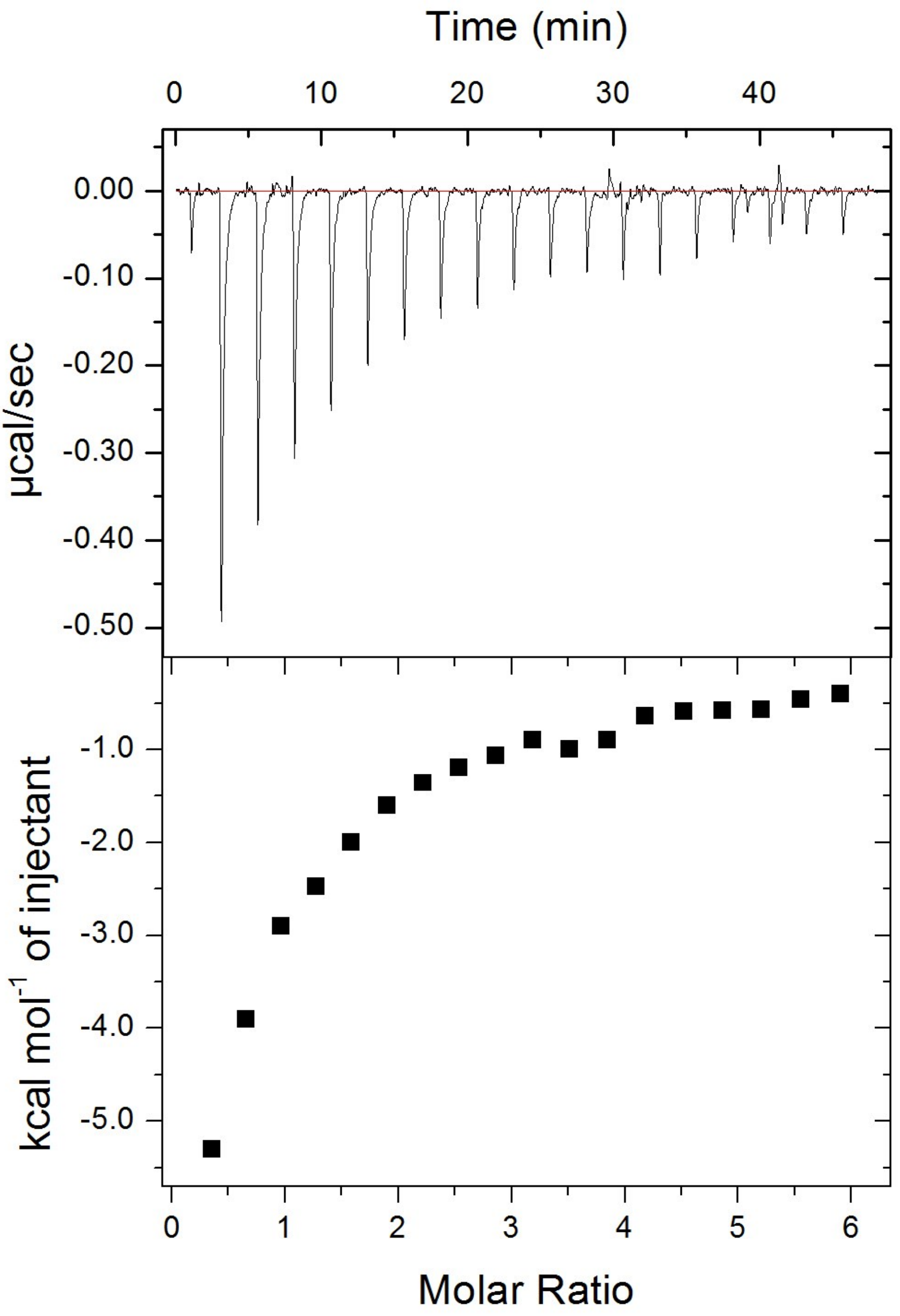

Figure S21. ITC raw data. Full ITC data (including raw data) for Zn^{2+} binding to 25 µM HSA in the presence of 2.5 mol. eq. of stearate, corresponding to data shown in Figure 2F.

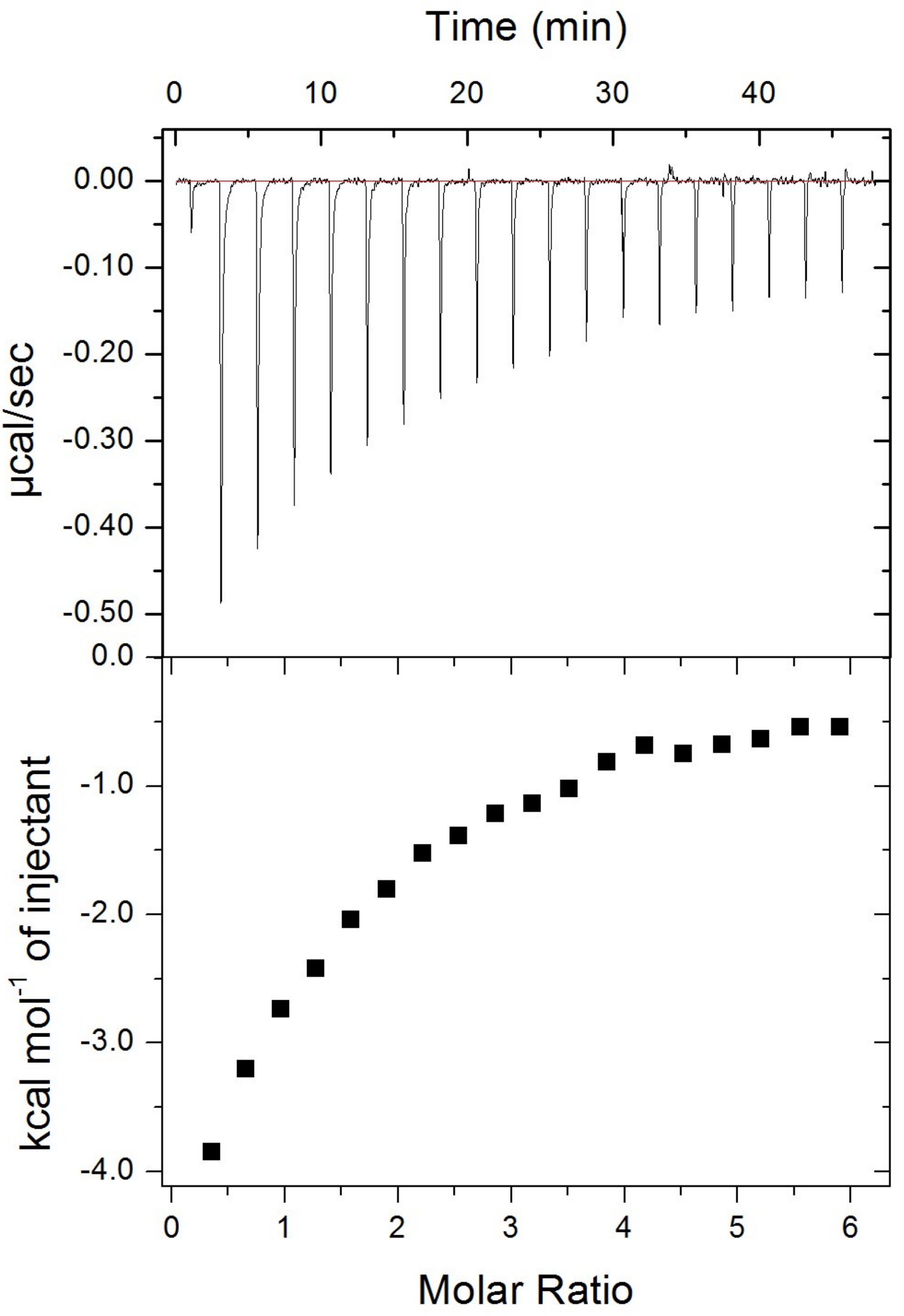

Figure S22. ITC raw data. Full ITC data (including raw data) for Zn^{2+} binding to 25 µM HSA in the presence of 4 mol. eq. of stearate, corresponding to data shown in Figure 2F.

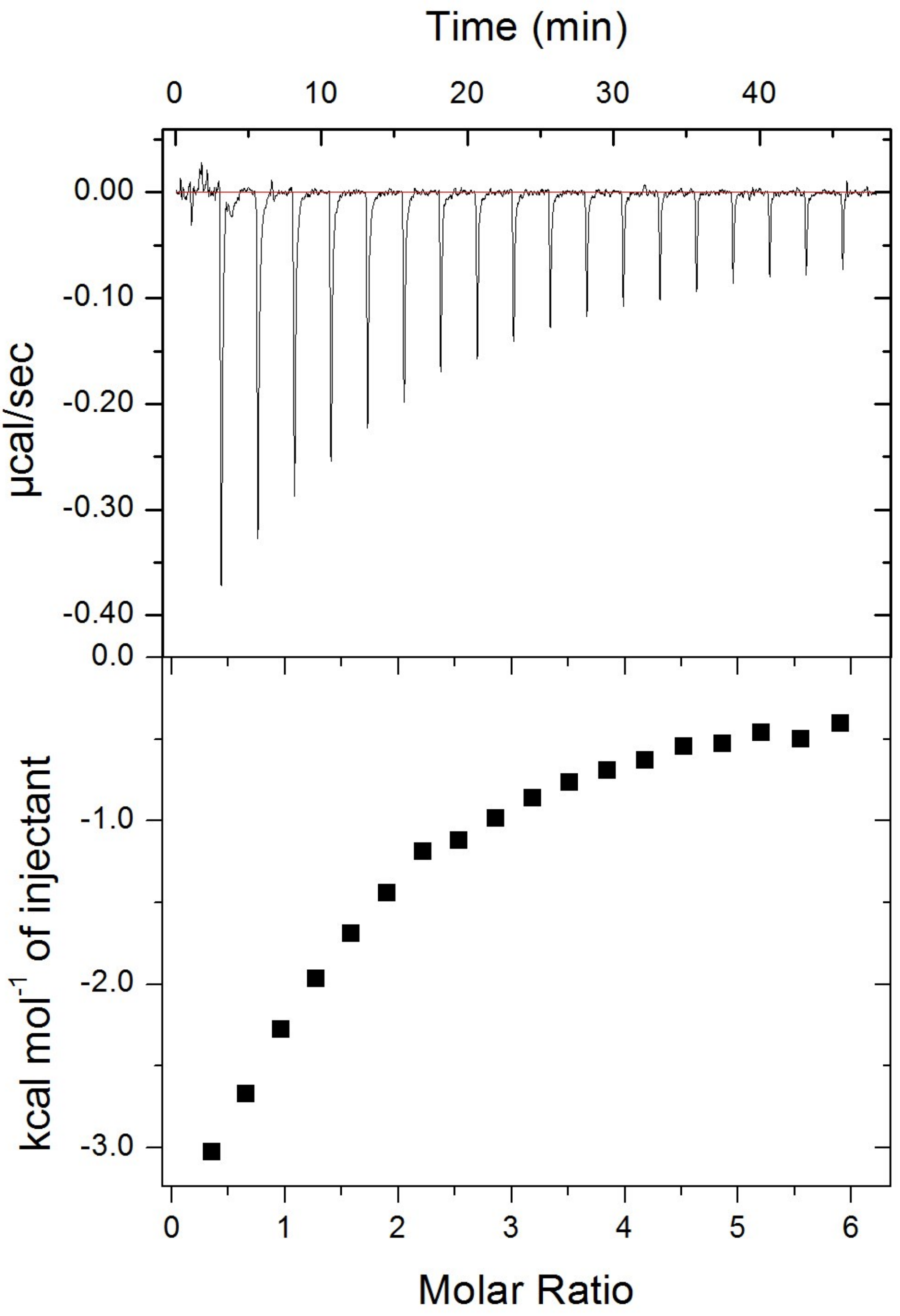

Figure S23. ITC raw data. Full ITC data (including raw data) for Zn^{2+} binding to 25 µM HSA in the presence of 5 mol. eq. of stearate, corresponding to data shown in Figure 2F.

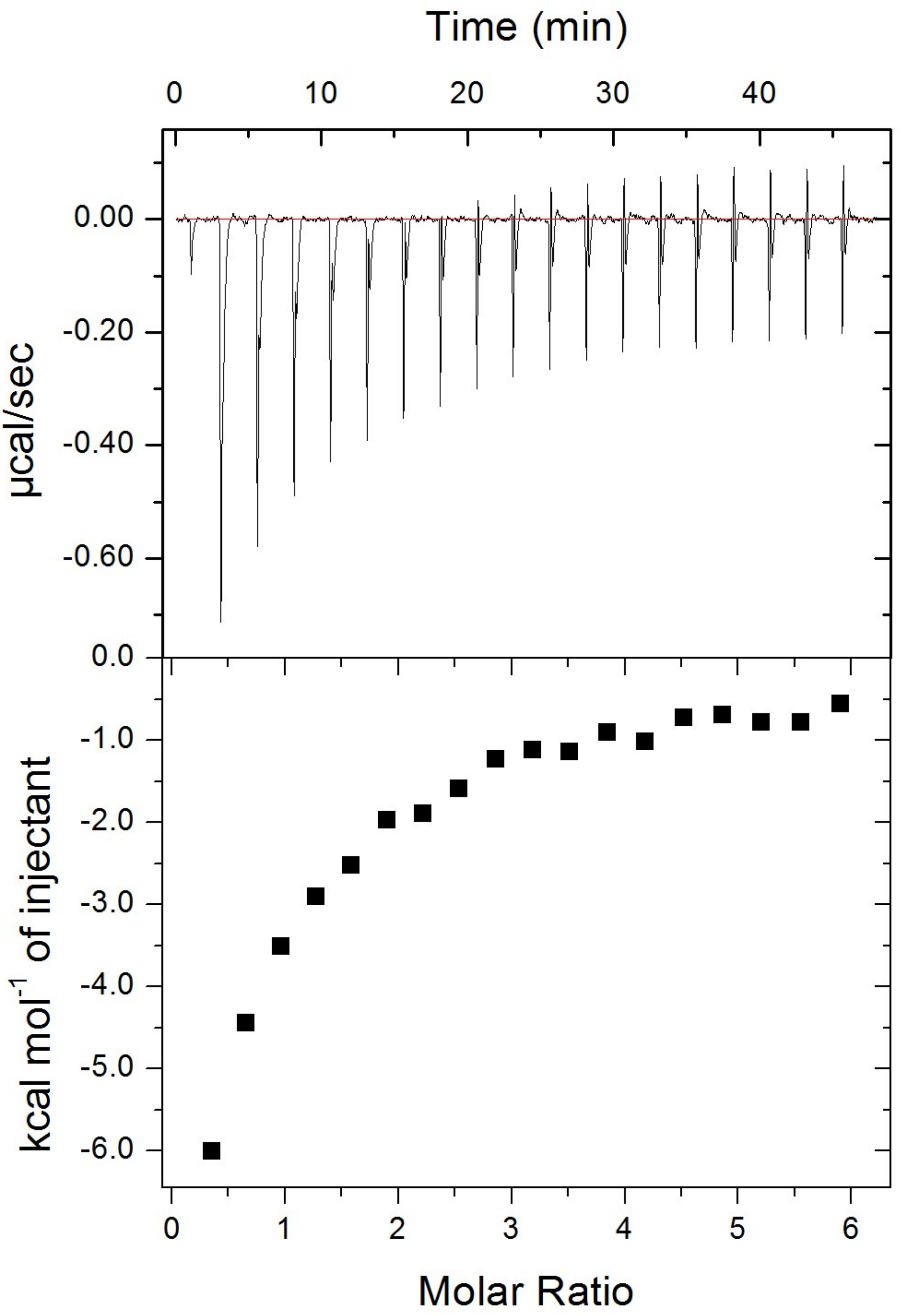

Figure S24. ITC raw data. Full ITC data (including raw data) for Zn^{2+} binding to 25 µM HSA in the presence of 2.5 mol. eq. of palmitoleate, corresponding to data shown in Figure 2G.

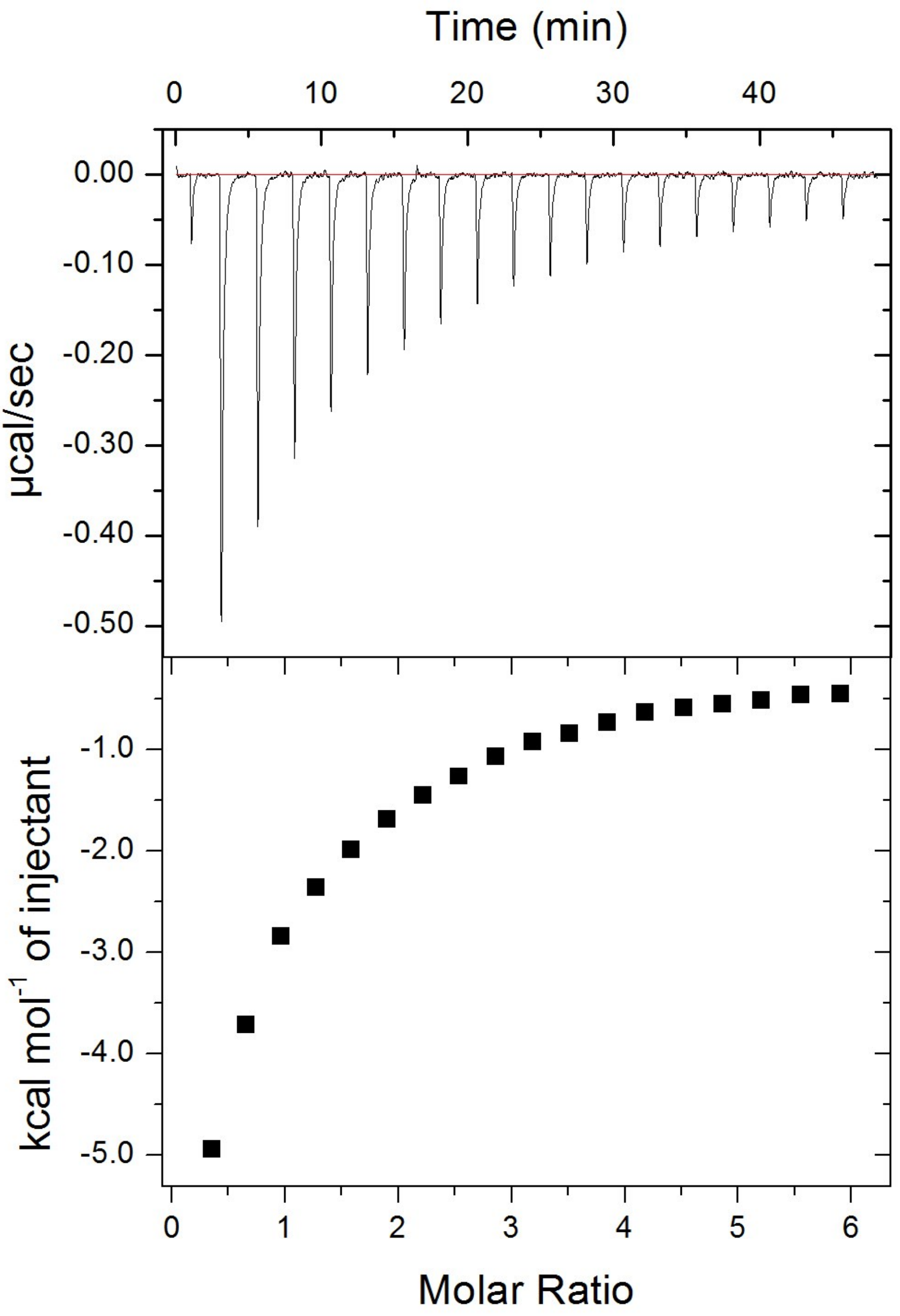

Figure S25. ITC raw data. Full ITC data (including raw data) for Zn^{2+} binding to 25 µM HSA in the presence of 4 mol. eq. of palmitoleate, corresponding to data shown in Figure 2G.

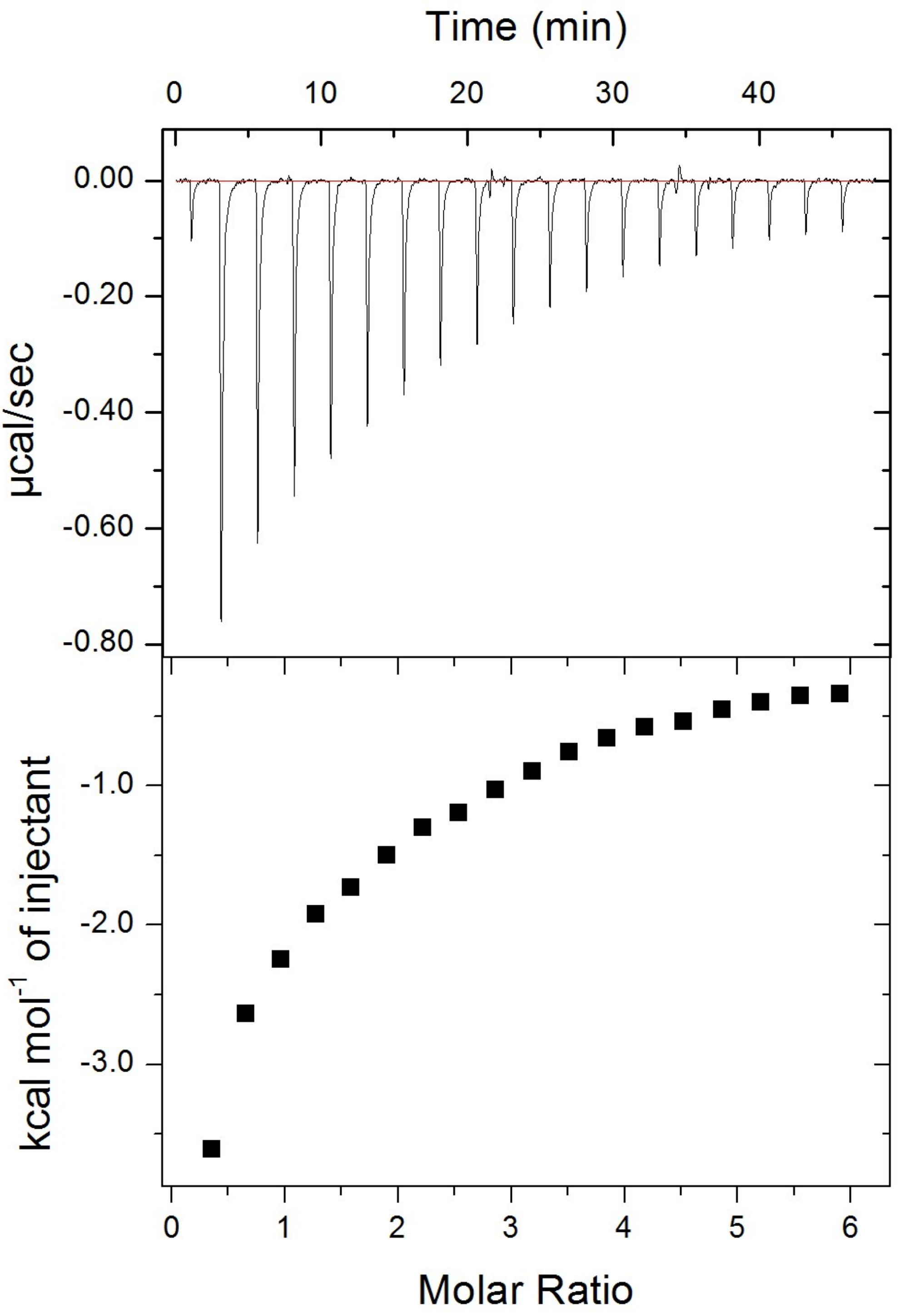

Figure S26. ITC raw data. Full ITC data (including raw data) for Zn^{2+} binding to 25 µM HSA in the presence of 5 mol. eq. of palmitoleate, corresponding to data shown in Figure 2G.

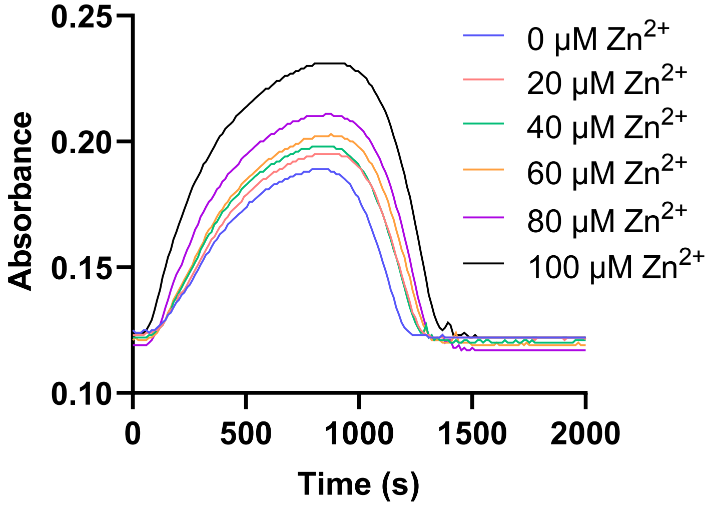

Figure S27. Representative raw data from turbidimetric fibrin clotting and lysis assays. Purified system, no myristate, 0-100 μM Zn^{2+}.

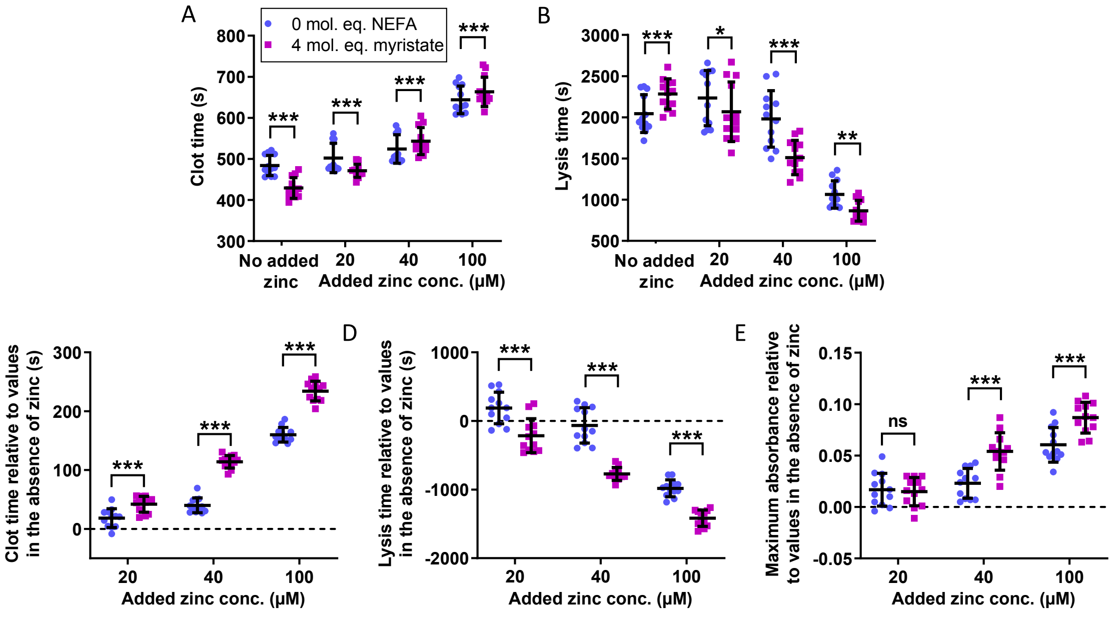

F

Zn^{2+} added to undiluted plasma (µM)	Available Zn^{2+} (µM)			Differential effect on clot time?	Differential effect on lysis time?
	Plasma	Plasma+NEFA	Difference		
**0**	0.07±0.01	0.27±0.01	0.20±0.02	***↑	***↓
**20**	0.68±0.01	3.13±0.24	2.5±0.24	***↑	***↓
**40**	1.54±0.03	7.0±0.6	5.5±0.6	***↑	***↓
**100**	6.85±0.40	20.5±2.1	13.7±2.1	***↑	***↓

Figure S28. Effects of Zn^{2+} and NEFAs on fibrin clot parameters in pooled plasma and effects relative to the parameter values in the absence of Zn^{2+}. Turbidimetric fibrin clotting and lysis assays were performed in pooled plasma diluted 6-fold in buffer (50 mM Tris, 100 mM NaCl, pH 7.4), with final concentrations of 7.5 mM $CaCl_2$, 0.03 U/ml thrombin, 20.8 ng/ml tPA, while 0-100 µM added $ZnCl_2$, as well as either 0 or 4 mol. eq. myristate relative to HSA concentration (n=12). Fibrin clot parameters were measured: (A) clot time and (B) lysis time and maximum absorbance (data shown in Figure 4). Two-way ANOVAs followed by Sidak's multiple comparisons tests were used to analyse the data. Both parameters showed a significant difference with the addition of Zn^{2+} ($p<0.0001$ for clot time and $p<0.0001$ for lysis time) as well as with 4 mol. eq. myristate ($p=0.0002$ for clot time $p<0.0001$ for lysis time). The parameter values relative to their values in the absence of Zn^{2+} (+/- myristate) were then calculated: (C) clot time, (D) lysis time and (E) maximum absorbance. Two-way ANOVAs followed by Sidak's multiple comparisons tests were used to analyse the data. All parameters showed a significant difference with the addition of Zn^{2+} ($p<0.0001$, for all three), as well as with 4 mol. eq. myristate ($p<0.0001$ and $p<0.0001$, $p=0.0013$ respectively). (F) Estimates for available Zn^{2+} in pooled plasma in presence and absence of 4 mol. eq. added myristate. "Available Zn^{2+}" was taken as the sum of free and Tris-bound Zn^{2+}. Citrate has been suggested to mask the effects of Zn^{2+} in coagulation; therefore, it was assumed that citrate-bound zinc would not be available to influence clotting or clot lysis. The entries in columns 5 and 6 refer to the data plotted in C and D). The data are represented as mean ± SD. Statistical significance is indicated by ns where $p>0.05$, * where $p<0.05$, ** where $p<0.01$ and *** where $p<0.001$.

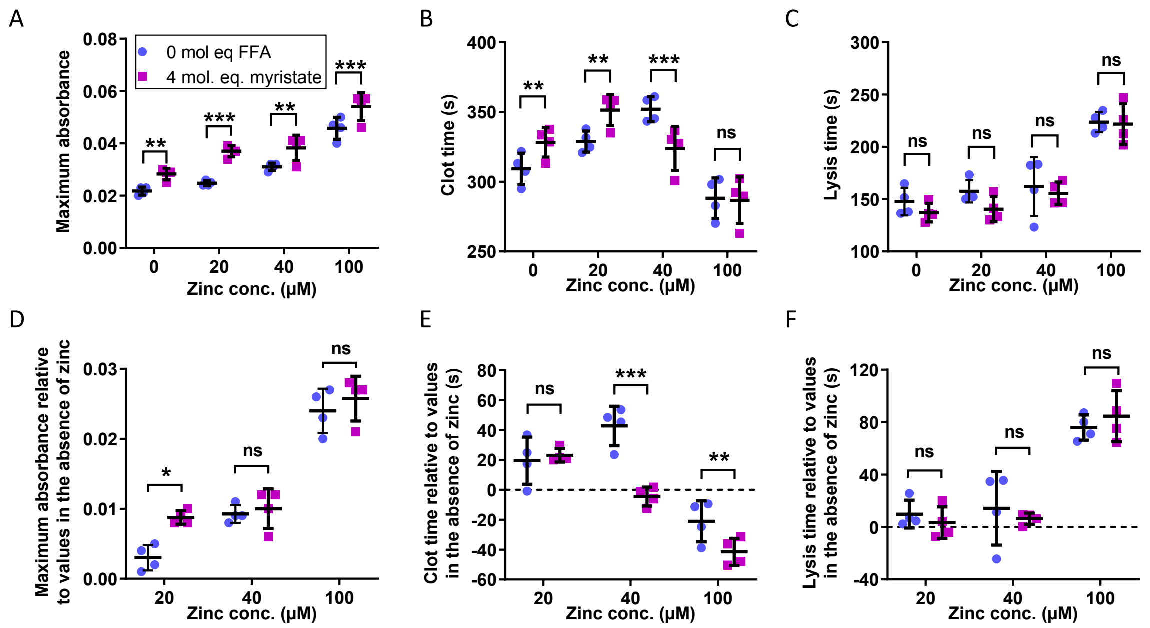

G

Added Zn (μM)	Available Zn^{2+} (μM)			Differential effect on max abs?	Differential effect on clot time?	Differential effect on lysis time?
	No NEFA	+NEFA	Difference			
**20**	0.75	6.0	5.3	* ↑	ns	ns
**40**	1.9	17.3	15.37	ns	*** ↓	ns
**100**	13.0	59.2	46.16	ns	** ↓	ns

Figure S29. Effects of Zn^{2+} and NEFAs on fibrin clot parameters in a purified system and effects relative to the parameter values in the absence of Zn^{2+}. Turbidimetric fibrin clotting and lysis assays were performed in buffer (50 mM Tris, 100 mM NaCl, pH 7.4) with a final concentration of 0.5 mg/mL (2.9 μM) fibrinogen, 100 μM HSA, 2.5 mM $CaCl_2$, 0.05 U/mL thrombin, 39 ng/mL tPA, 3.12 μg/mL plasminogen, 0-100 μM $ZnCl_2$, and either 0 or 4 mol. eq. myristate relative to HSA concentration (n=4). Fibrin clot parameters including (A) maximum absorbance, (B) clot time and (C) lysis time were measured. Two-way ANOVAs followed by Sidak's multiple comparisons tests were used to analyse the data. Maximum absorbance, clot time and lysis time significantly increased in the presence of Zn^{2+} ($p<0.0001$ for all). Clot time and lysis time were not significantly altered by the presence of 4 mol. eq. myristate, but maximum absorbance increased in the presence of myristate ($p=0.0276$). The parameter values relative to their values in the absence of Zn2+ (+/- myristate) were then calculated: (D) maximum absorbance, (E) clot time and (F) lysis time. Two-way ANOVAs followed by Sidak's multiple comparisons tests were used to analyse the data. Maximum absorbance, clot time and lysis time significantly increased in the presence of Zn^{2+} ($p<0.0001$ for all). Maximum absorbance and clot time increased in the presence of 4 mol. eq. myristate ($p= 0.0455$ and $p=0.0194$ respectively), while lysis time was not significantly altered. The data are represented as mean ± SD. On the abscise axes, "0 μM Zn^{2+}" refers to no added Zn^{2+} in the system. (G) Estimates for available Zn^{2+} in the purified system in presence and absence of 4 mol. eq. added myristate. "Available Zn^{2+}" was taken as the sum of free and Tris-bound Zn^{2+}. The HSA concentrations used for the calculation was 100 μM. The differential effects on maximum absorbance, clot time and lysis time are from D-F. Statistical significance is indicated by ns where $p>0.05$, * where $p<0.05$, ** where $p<0.01$ and *** where $p<0.001$.

Table S6. Demographic information and the results from plasma analysis for the T2DM and control subjects. All values are presented as mean ± standard deviation. The *P*-values were calculated by Student's *t*-test.

	Controls (n=18)	Patients with T2DM (n=54)	P-values
Age (years)	57.1 ± 8.9	60.9 ± 7.6	0.0814
Sex (% female)	56	13	0.0001
Weight (kg)	70.2 ± 12.9	96.3 ± 17.8	<0.0001
BMI	25.0 ± 3.2	32.6 ± 5.3	<0.0001
Number of individuals who smoke	1	10	-
Number of individuals who have had microvascular events	-	22	-
Number of individuals who have had macrovascular events	-	31	-
Numbers of individuals with familial history of autoimmunity	2	7	-
Numbers of individuals with familial history of Huntington's disease	3	30	-
Diabetes duration (months)	-	139 ± 78	-
Fasting glucose (mM)	4.8 ± 0.5	10.3 ± 4.6	<0.0001
HbA1c (mmol/mol)	37.6 ± 4.3	72.4 ± 22.8	<0.0001
Triglyceride (mM)	0.95 ± 0.28	2.1 ± 2.0	0.0313
Cholesterol (mM)	5.3 ± 0.7	3.8 ± 0.8	<0.0001
LDL (mM)	3.0 ± 0.7	1.9 ± 0.5	<0.0001
HDL (mM)	1.9 ± 0.5	1.1 ± 0.3	<0.0001
Cholesterol/HDL ratio	3.1 ± 0.9	3.7 ± 0.9	0.0198
Urea concentration (mM)	5.7 ± 1.2	6.3 ± 2.4	0.3399
Platelet count (×1000/µL)	258 ± 73[*]	251 ± 53	0.8367
Fibrinogen (g/L)	2.7 ± 0.3[*]	2.7 ± 0.5	0.9005

[*] data only available for 3 samples

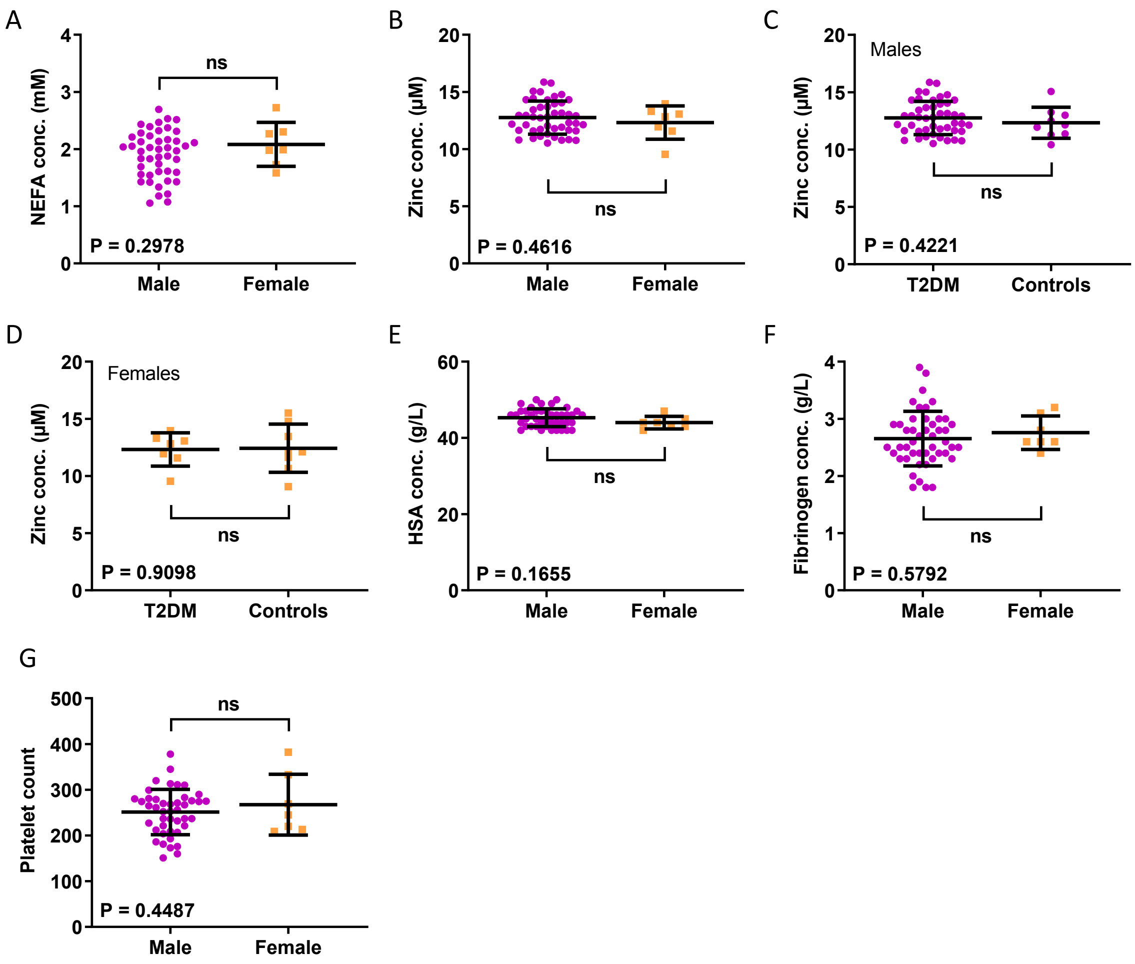

Figure S30. Comparisons of NEFA, zinc, HSA and fibrinogen concentrations and of platelet count between sexes in plasma samples from individuals with T2DM and controls. Differences in (A) plasma NEFA concentrations and (B) total plasma zinc concentrations between sexes in patients with T2DM are shown. Differences in total plasma zinc concentration in individuals with T2DM and controls in (C) males and (D) females are shown. Differences in (E) HSA concentrations, (F) plasma fibrinogen concentrations and (G) platelet count between sexes in patients with T2DM are shown. (A-G) In all cases no statistically significant differences were found between the respective parameters (ns; $p>0.05$). The data are represented as mean ± SD. T-tests were used to analyse the data.

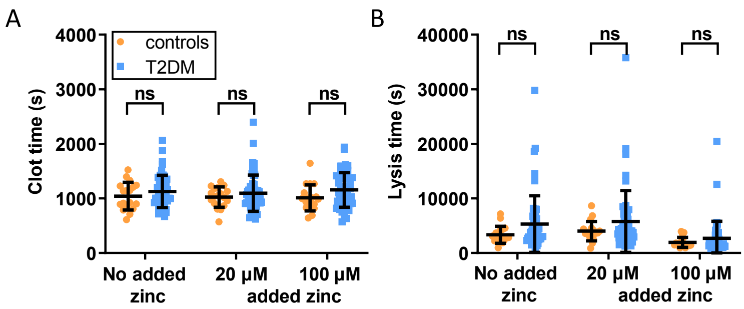

Figure S31. Comparison of the clot formation and lysis parameters in plasma samples from patient with T2DM and controls. Turbidimetric fibrin clotting assays were performed in plasma samples with final concentration of plasma diluted 3-fold in buffer, 7.5 mM $CaCl_2$, 0.03 U/mL thrombin and 0, 20 or 100 µM added $ZnCl_2$ (n=54 for diabetes subjects and n=18 for controls). (A) Clot time was calculated from those experiments. Turbidimetric fibrin clotting and lysis assays were then performed with final concentrations of: plasma diluted 6-fold in buffer, 7.5 mM $CaCl_2$, 0.03 U/mL thrombin, 20.8 ng/mL tPA and 0, 20 or 100 µM added $ZnCl_2$. (B) Lysis time was calculated from those experiments. The data are represented as mean ± SD. Two-way ANOVAs followed by Sidak's multiple comparison tests were used to analyse the data. Lysis time increased significantly in T2DM subjects compared to controls (p=0.0258, two-way ANOVA), as well as in the presence of Zn^{2+} (p=0.0258); however multiple comparison tests showed that individual comparisons between control and T2DM at "no added zinc", "20 µM added zinc" or "100 µM added zinc" individually were not significant. Clot time was not significantly altered in the presence or absence of Zn^{2+} or when comparing the T2DM group to the control group. Statistical significance is indicated with ns where p>0.05.

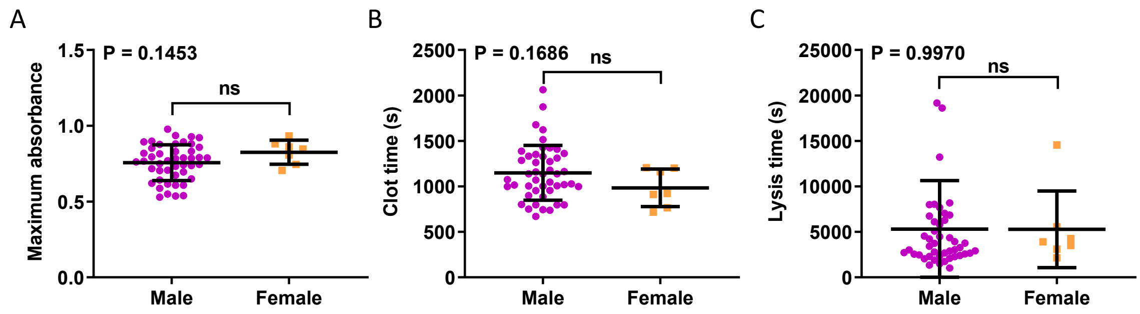

Figure S32. Comparison of clotting parameters between sexes in plasma samples from patients with T2DM and controls. (A) Maximum absorbance, (B) clot time and (C) lysis time. No difference was found. The data are represented as mean ± SD. T-tests were used to analyse the data. Statistical significance is indicated with ns where $p>0.05$.

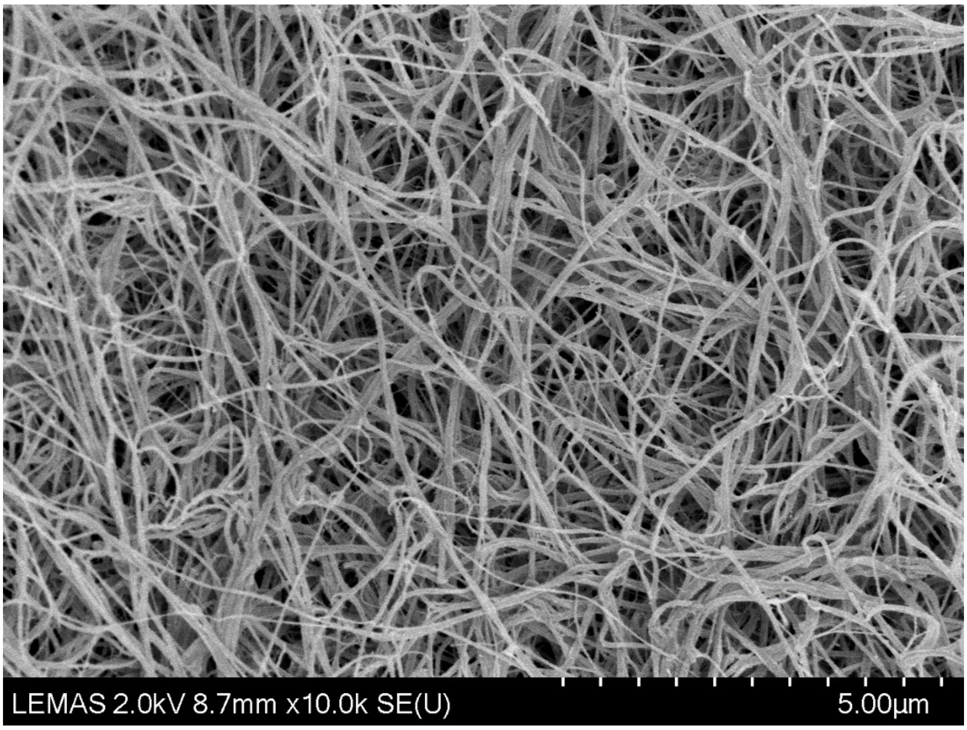

Figure S33. Representative image from SEM experiments. Purified system, no zinc. The image was viewed and photographed at ×10,000 magnification using a SU8230 SEM.

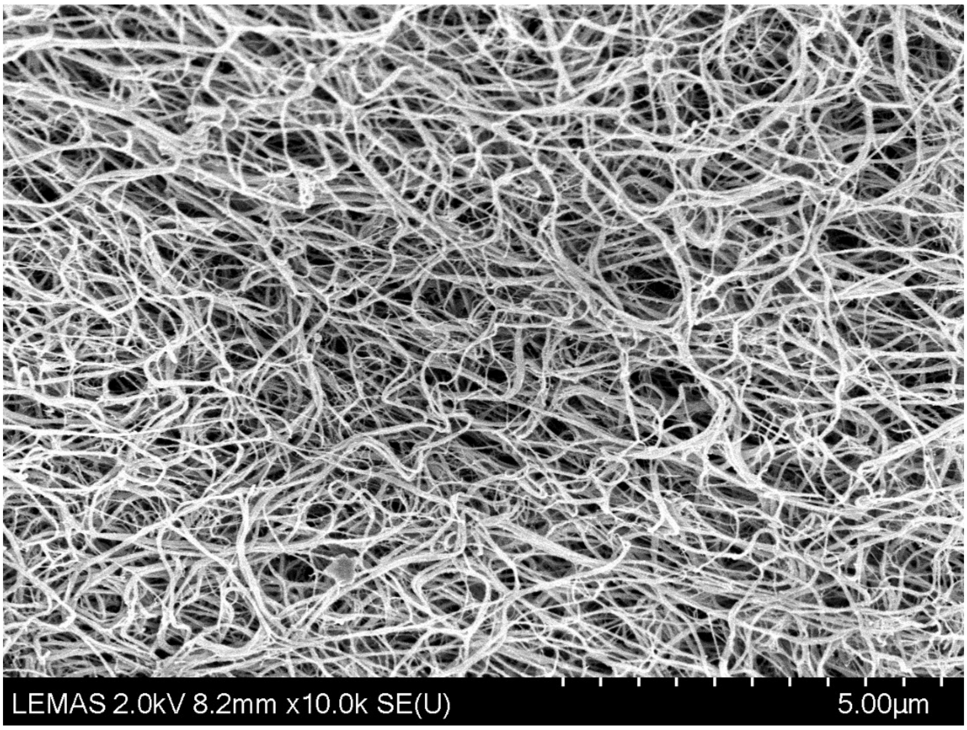

Figure S34. Representative image from SEM experiments. Purified system, 18 µM zinc. The image was viewed and photographed at ×10,000 magnification using a SU8230 SEM.

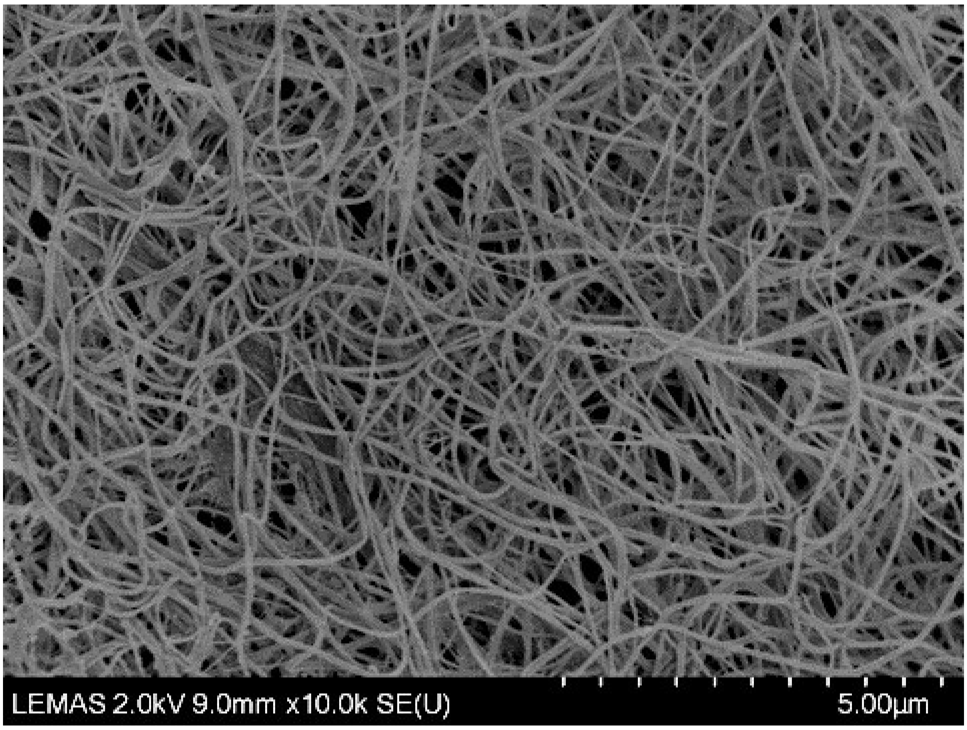

Figure S35. Representative image from SEM experiments. Controls, no zinc. The image was viewed and photographed at ×10,000 magnification using a SU8230 SEM.

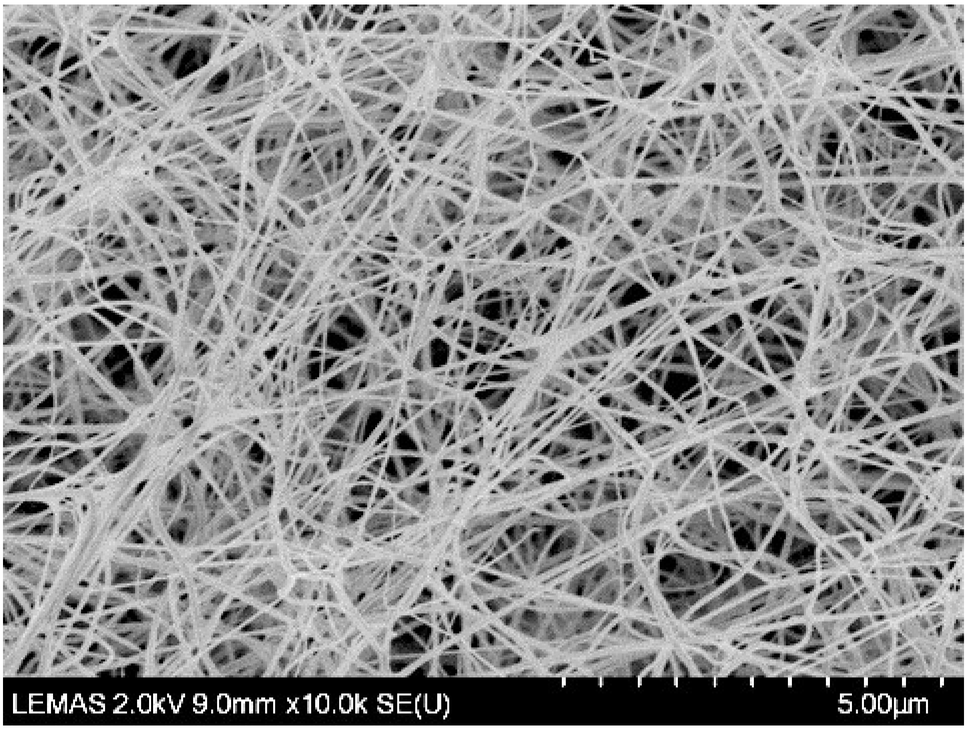

Figure S36. Representative image from SEM experiments. Controls, 50 µM zinc. The image was viewed and photographed at ×10,000 magnification using a SU8230 SEM.

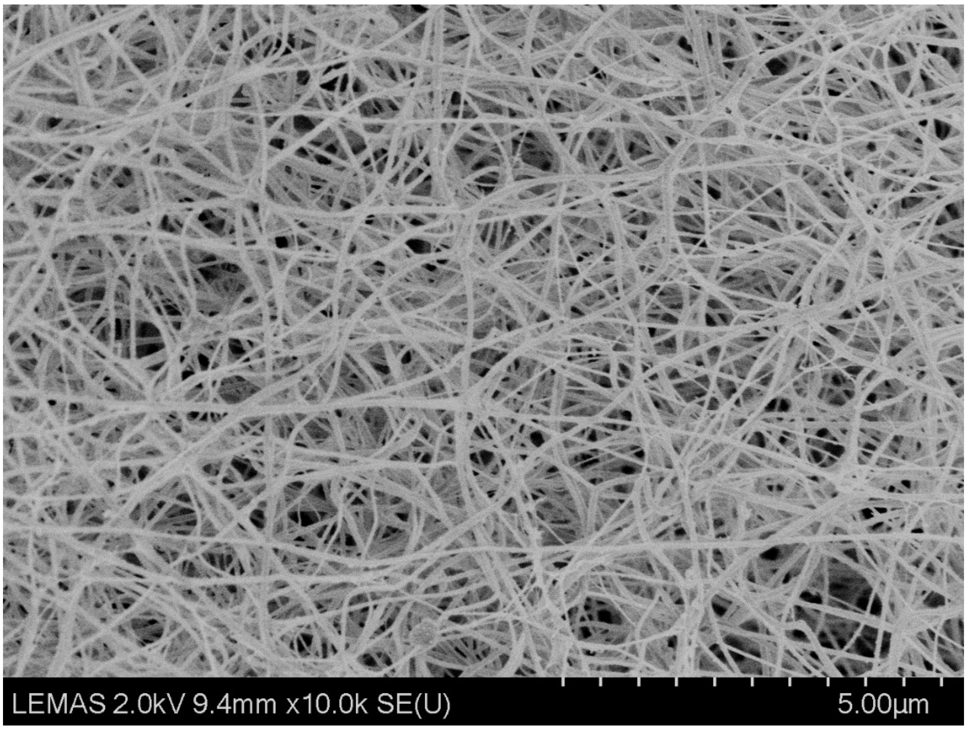

Figure S37. Representative image from SEM experiments. T2DM, no zinc. The image was viewed and photographed at ×10,000 magnification using a SU8230 SEM.

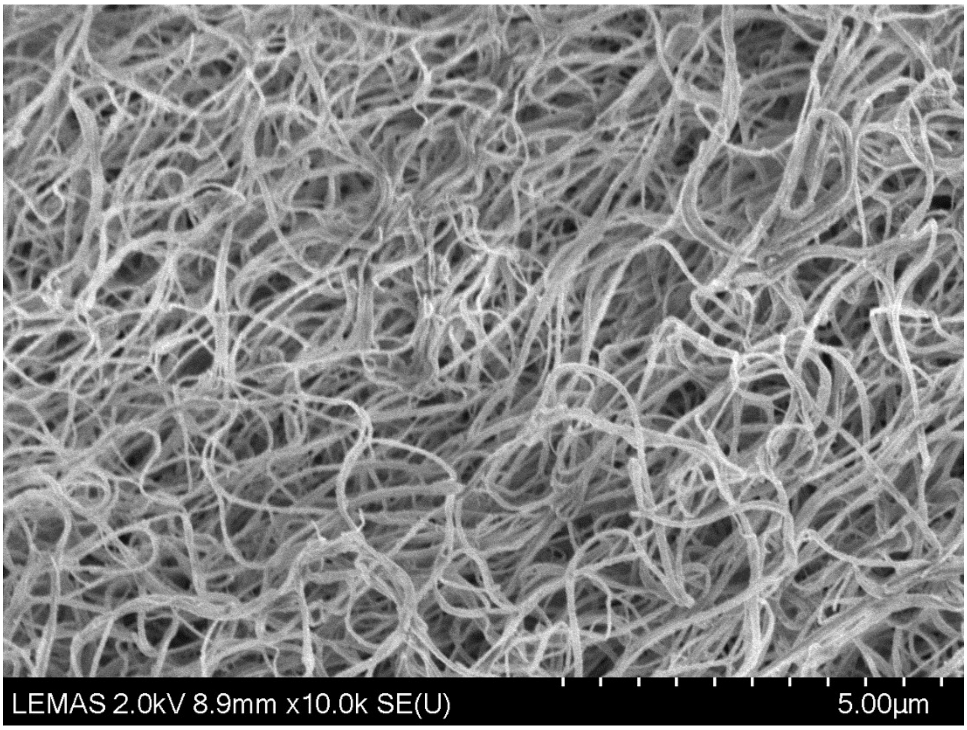

Figure S38. Representative image from SEM experiments. T2DM, 50 µM zinc. The image was viewed and photographed at ×10,000 magnification using a SU8230 SEM.

A

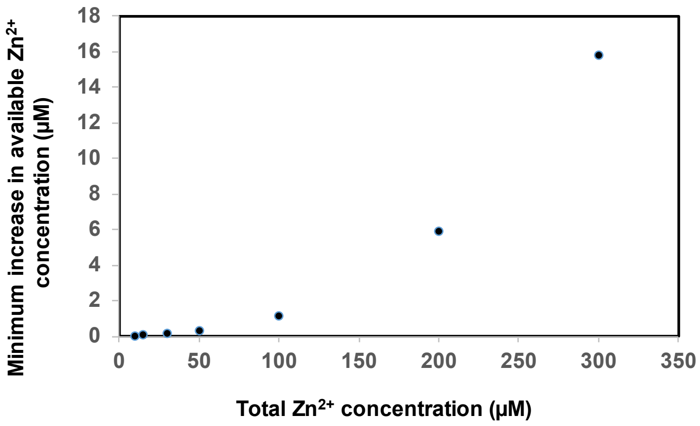

B

Total Zn^{2+} concentration (µM)	Minimum available Zn^{2+} concentration (µM)		
	Plasma	Plasma+NEFA	Difference
**10**	0.02	0.08	0.06
**15**	0.03	0.12	0.09
**30**	0.07	0.26	0.19
**50**	0.12	0.49	0.38
**100**	0.25	1.42	1.17
**200**	0.62	6.54	5.92
**300**	1.20	17.01	15.81

Figure S39. Minimum increase in available Zn^{2+} concentration upon addition of 4 mol. eq. of myristate to 600 mM HSA, in dependence on total Zn^{2+} concentration. Speciation calculations are based on a reduction to 16.3% binding capacity of site A, and also included 150 µM citrate (typical endogenous concentration). These estimates highlight that during platelet activation, the presence of NEFAs might result in sizeable changes of available Zn^{2+} in plasma.
